# Accelerated deciphering of the genetic architecture of agricultural economic traits in pigs using a low-coverage whole-genome sequencing strategy

**DOI:** 10.1093/gigascience/giab048

**Published:** 2021-07-20

**Authors:** Ruifei Yang, Xiaoli Guo, Di Zhu, Cheng Tan, Cheng Bian, Jiangli Ren, Zhuolin Huang, Yiqiang Zhao, Gengyuan Cai, Dewu Liu, Zhenfang Wu, Yuzhe Wang, Ning Li, Xiaoxiang Hu

**Affiliations:** State Key Laboratory of Agrobiotechnology, College of Biological Sciences, China Agricultural University, No. 2 Yuanmingyuan west road, Haidian district, Beijing 100193, China; State Key Laboratory of Agrobiotechnology, College of Biological Sciences, China Agricultural University, No. 2 Yuanmingyuan west road, Haidian district, Beijing 100193, China; State Key Laboratory of Agrobiotechnology, College of Biological Sciences, China Agricultural University, No. 2 Yuanmingyuan west road, Haidian district, Beijing 100193, China; National Engineering Research Center for Breeding Swine Industry, South China Agricultural University, No. 483 Wushan road, Tianhe district, Guangdong 510640, China; State Key Laboratory of Agrobiotechnology, College of Biological Sciences, China Agricultural University, No. 2 Yuanmingyuan west road, Haidian district, Beijing 100193, China; State Key Laboratory of Agrobiotechnology, College of Biological Sciences, China Agricultural University, No. 2 Yuanmingyuan west road, Haidian district, Beijing 100193, China; State Key Laboratory of Agrobiotechnology, College of Biological Sciences, China Agricultural University, No. 2 Yuanmingyuan west road, Haidian district, Beijing 100193, China; State Key Laboratory of Agrobiotechnology, College of Biological Sciences, China Agricultural University, No. 2 Yuanmingyuan west road, Haidian district, Beijing 100193, China; National Engineering Research Center for Breeding Swine Industry, South China Agricultural University, No. 483 Wushan road, Tianhe district, Guangdong 510640, China; National Engineering Research Center for Breeding Swine Industry, South China Agricultural University, No. 483 Wushan road, Tianhe district, Guangdong 510640, China; National Engineering Research Center for Breeding Swine Industry, South China Agricultural University, No. 483 Wushan road, Tianhe district, Guangdong 510640, China; State Key Laboratory of Agrobiotechnology, College of Biological Sciences, China Agricultural University, No. 2 Yuanmingyuan west road, Haidian district, Beijing 100193, China; National Research Facility for Phenotypic and Genotypic Analysis of Model Animals (Beijing), China Agricultural University, No. 2 Yuanmingyuan west road, Haidian district, Beijing 100193, China; State Key Laboratory of Agrobiotechnology, College of Biological Sciences, China Agricultural University, No. 2 Yuanmingyuan west road, Haidian district, Beijing 100193, China; State Key Laboratory of Agrobiotechnology, College of Biological Sciences, China Agricultural University, No. 2 Yuanmingyuan west road, Haidian district, Beijing 100193, China

**Keywords:** low-coverage sequencing, GWAS, genotyping, pig, genetic architecture, agricultural traits

## Abstract

**Background:**

Uncovering the genetic architecture of economic traits in pigs is important for agricultural breeding. However, high-density haplotype reference panels are unavailable in most agricultural species, limiting accurate genotype imputation in large populations. Moreover, the infinitesimal model of quantitative traits implies that weak association signals tend to be spread across most of the genome, further complicating the genetic analysis. Hence, there is a need to develop new methods for sequencing large cohorts without large reference panels.

**Results:**

We describe a Tn5-based highly accurate, cost- and time-efficient, low-coverage sequencing method to obtain 11.3 million whole-genome single-nucleotide polymorphisms in 2,869 Duroc boars at a mean depth of 0.73×. On the basis of these single-nucleotide polymorphisms, a genome-wide association study was performed, resulting in 14 quantitative trait loci (QTLs) for 7 of 21 important agricultural traits in pigs. These QTLs harbour genes, such as *ABCD4* for total teat number and *HMGA1* for back fat thickness, and provided a starting point for further investigation. The inheritance models of the different traits varied greatly. Most follow the minor-polygene model, but this can be attributed to different reasons, such as the shaping of genetic architecture by artificial selection for this population and sufficiently interconnected minor gene regulatory networks.

**Conclusions:**

Genome-wide association study results for 21 important agricultural traits identified 14 QTLs/genes and showed their genetic architectures, providing guidance for genetic improvement harnessing genomic features. The Tn5-based low-coverage sequencing method can be applied to large-scale genome studies for any species without a good reference panel and can be used for agricultural breeding.

## Background

Genome-wide association studies (GWAS) have identified thousands of genetic variants associated with complex traits in humans and agricultural species [1, 2]. The mapping resolution relies on the density of genetic markers that can reveal linkage disequilibrium (LD) patterns in sufficiently large populations [3, 4]. Despite the declining cost of sequencing, it is still expensive for agricultural breeding studies to perform whole-genome sequencing (WGS) of all individuals in a large cohort (thousands of individuals). In many scenarios, imputation-based strategies, which impute low-density panels to higher densities, offer an alternative to systematic genotyping or sequencing [5, 6]. Array-based genotype imputation is widely used in agricultural species [7, 8]. However, the imputation accuracy of this strategy depends crucially on the reference panel sizes and genetic distances between the reference and target populations. Hence, the unavailability of large reference panels and array designs for target populations in agricultural species limits the improvement offered by array-based genotype imputation [9, 10]. Inaccurate imputations influence the results of follow-up population genetic analyses.

Low-coverage sequencing (LCS) of a large cohort has been proposed to be more informative than sequencing fewer individuals at a higher coverage rate [11–13]. Sample sizes and haplotype diversity could be more critical than sequencing depth in determining the genotype accuracy of most segregating sites and increasing the power of association studies. Overall, LCS has been proven to have greater power for trait mapping than the array-based genotyping method in human studies [14]. To date, LCS-based genotype imputation has been used in many studies using various populations and genotyping algorithms [15–19]. In particular, the STITCH imputation algorithm overcomes the barrier of the lack of good reference panels in non-human species and is even applicable in studies with extremely low sequencing depths [15, 20]. This is a promising approach for agricultural animals without large reference panels and can be used in the areas of functional genetic mapping and genomic breeding. However, to date, no reports have been published on this.

Several large-scale WGS projects have been completed [21–25]. These projects were designed to identify the underlying mechanisms that drive hereditary diseases in humans, as well as for use in genomic selection in the breeding of agricultural species [26–28]. The infinitesimal model, which describes the inheritance patterns of quantitative traits, appears to be successful [29, 30]; however, it is unclear how many genes play important roles in driving different kinds of complex traits. In addition, artificial selection provides a driving force for the rapid evolution of agricultural species, which further brings about the fixation of selection regions and differentials in the inheritance model. This process might produce a very different result for the same trait between studies owing to the different genetic backgrounds of the research population. Therefore, care should be taken when determining the GWAS results for a specific population. Such information, which might be helpful for understanding the genetic mechanism of a complex trait, could be informative for further application of genomic selection in animal breeding.

In this study, we developed a new highly accurate, cost- and time-efficient LCS method to obtain high-density single-nucleotide polymorphism (SNP) markers for a large Duroc pig population [31]. By assessing 21 important agricultural traits in commercial pig herds, we performed genome-wide association and fine-mapping analyses with high resolution and compared the results of the inheritance model in depth. We also proved that artificial selection plays a significant role in altering the genetic architecture of agricultural animals, especially for loci that affect economically important traits. The LCS strategy offers a powerful method for further agricultural breeding.

## Data Description

A Tn5-based protocol was used to prepare sequencing libraries of each pig at a low cost (reagent cost: $2.60/library) as described in the Methods section. The libraries were sequenced on the Illumina (PE 150 model, 2 libraries) and the BGI platform (PE 100 model, 28 libraries) (Supplementary Table S1). The results generated by the BGI platform had a smaller number of PCR duplicates (2.23%), a higher number of good index reads (97.10%), and higher genome coverage (98.55%) than the Illumina dataset (10.82% PCR duplicates, 93.64% good index reads, and 98.50% genome coverage). Overall, the total output of the 2,869 boars approached 5.32 TB (terabytes), and most (96.74%) of the reads were successfully mapped to the pig reference genome Sscrofa11.1. Each animal was sequenced at a mean depth of 0.73 ± 0.17×. Moreover, high-depth resequencing (n = 37, selected from the 2,869 boars, mean 15.15×/sample), SNP Array (n = 42, GeneSeek Genomic Profiler Porcine 80K SNP Array, GGP-80) genotyping, and Fluidigm Integrated Fluidic Circuit (IFC) direct genotyping (n = 191 for 16 SNP loci) were performed on the selected Duroc core boars of this population, and the results were used for downstream accuracy evaluation. The 21 associated phenotypes used in this study are shown in Table 1 and Supplementary Fig. S1.

**Table 1:** QTLs: mapping and contribution to heritability

Phenotype	No.	Mean ± SD	Significant threshold^a^	QTL No.	Variance explained (%)^b^	Gene No.^c^
Total teat number (TTN)	2,797	10.73 ± 1.07	4.55	6	8.86	52
Left teat number (LTN)	2,797	5.35 ± 0.66	4.81	2	3.16	14
Right teat number (RTN)	2,797	5.38 ± 0.64	4.79	5	6.03	56
Back fat thickness at 100 kg (BF, mm)	2,796	10.99 ± 2.66	4.67	4	2.40	55
Loin muscle depth at 100 kg (LMD, mm)	2,796	46.15 ± 3.93	5.36	2	1.27	15
Loin muscle area at 100 kg (LMA, mm^2^)	2,795	36.25 ± 3.60		0	0	0
Lean meat percentage at 100 kg (LMP, %)	2,795	54.02 ± 1.58	5.50	1	1.19	48
Time spent eating per day (TPD, min)	2,602	63.02 ± 9.85	6.10	1	1.08	28
Average daily feed intake (ADFI, kg)	2,602	2.00 ± 0.20		0	0	0
Number of visits to feeder per day (NVD)	2,602	7.30 ± 1.83		0	0	0
Time spent eating per visit (TPV, min)	2,602	10.06 ± 2.79		0	0	0
Feed intake rate (FR, g/min)	2,602	32.37 ± 5.19		0	0	0
Feed intake per visit (FPV, kg)	2,602	290.6 ± 75.87		0	0	0
Feed conversion rate (FCR)	2,691	2.19 ± 0.19		0	0	0
Average daily gain (0–30 kg) (ADG30, g)	2,795	354.8 ± 38.72		0	0	0
Age to 30 kg live weight (AGE30, day)	2,796	80.49 ± 8.57		0	0	0
Average daily gain (30–100 kg) (ADG100, g)	2,795	633.8 ± 37.12		0	0	0
Age to 100 kg live weight (AGE100, day)	2,796	155.5 ± 9.20		0	0	0
Body length (BL, cm)	1,844	117.60 ± 2.91		0	0	0
Body height (BH, cm)	1,844	62.19 ± 1.55		0	0	0
Circumference of cannon bone (CC, cm)	1,844	17.81 ± 0.54		0	0	0

a Log_10_(*P*) value when FDR < 0.05.

b Total phenotypic variance explained by QTLs.

c Total gene number included in QTLs.

## Analyses

### Processing pipeline of the low-coverage strategy and accuracy evaluation

**Figure 1:**
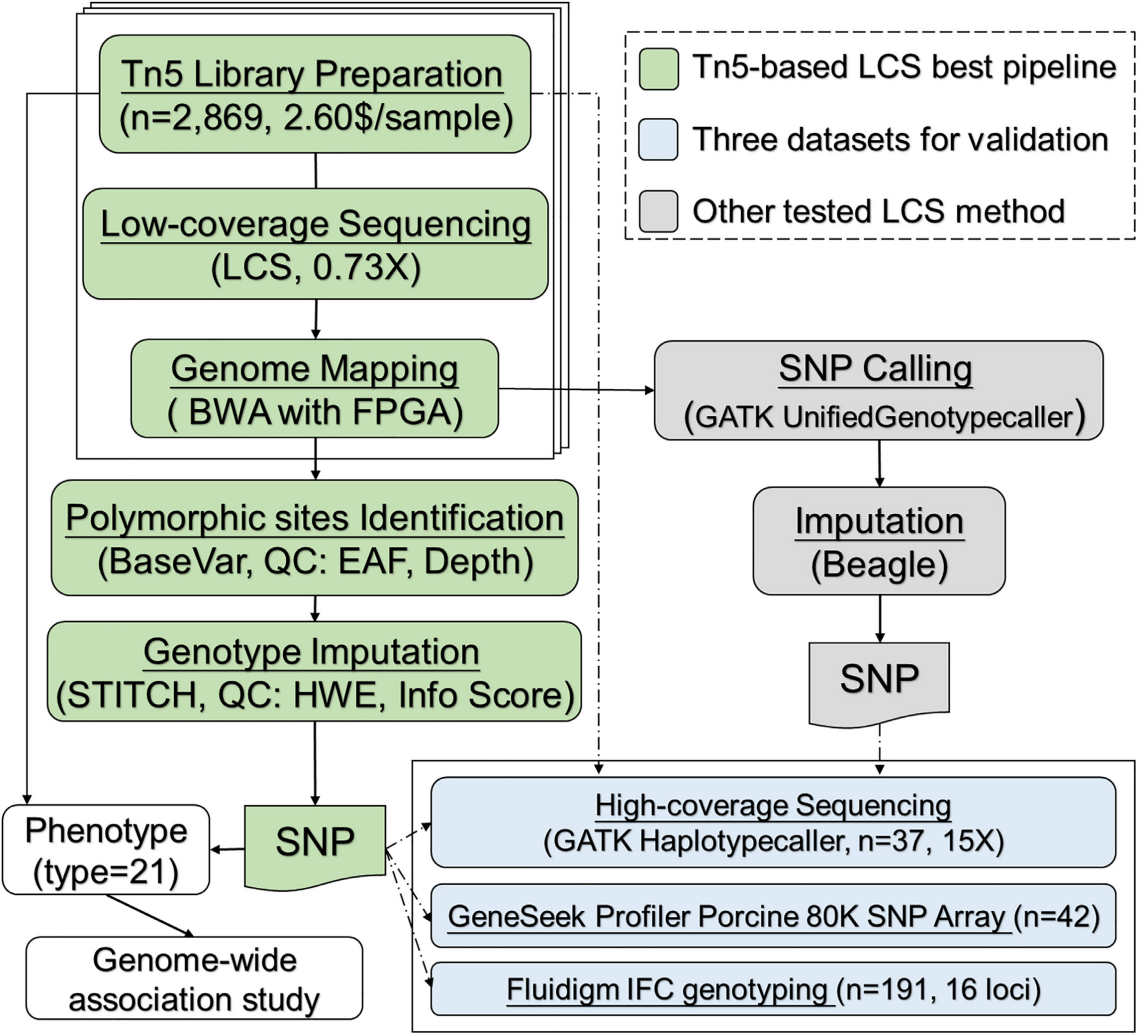
Low-coverage sequencing (LCS) study design. The flow chart summarizes the steps used to identify and impute polymorphic sites, where the green block represents the highly accurate pipeline used for Tn5-based LCS analysis (BaseVar-STITCH). We also generated SNP results using the GATK-Beagle pipeline (grey) and compared them with those obtained using the BaseVar-STITCH method. Three datasets (blue) were used to assess the accuracy of the results. The BaseVar-STITCH pipeline was used in the GWAS presented in this study. BWA: Burrows-Wheeler Aligner; HWE: Hardy-Weinberg equilibrium; QC: quality control.

**Figure 2:**
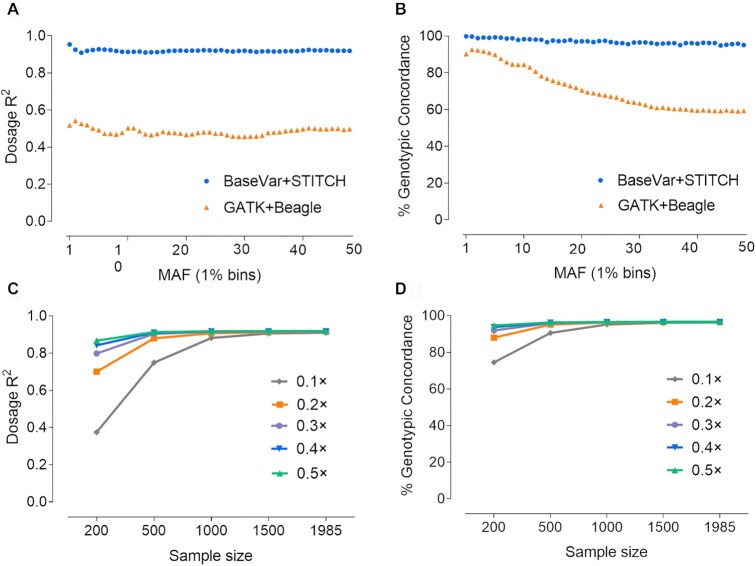
Performance of BaseVar-STITCH on different minor allele frequencies (MAFs) and sample sizes. The validation dataset is the high-coverage sequencing results of 37 individuals genotyped by GATK best practices (HaplotypeCaller model). (**A**) and (**B**) show a comparison of the dosage *R*^2^ and genotypic concordance values (%) between the BaseVar-STITCH for low-coverage sequencing (LCS) (blue) and the GATK-Beagle (orange) pipelines, and (**C**) and (**D**) show the comparison of the dosage *R*^2^ and genotypic concordance values (%) among different sequencing depths.

Traditional standard methods for SNP calling, such as those implemented in GATK and SAMtools, are mainly used in high-depth resequencing methods. However, owing to the low depth of each base, erroneous SNPs and genotypes could be called using such methods, especially for the GATK HaplotypeCaller algorithm (single sample local *de novo* assembly) [32]. Hence, in this study, we mainly applied the BaseVar algorithm [33] to identify polymorphic sites and infer allele frequencies, and STITCH to impute SNPs. We also tested the performance of GATK (UnifiedGenotypeCaller)-Beagle algorithms in LCS data. The high-depth sequencing data and SNP chip (GGP-80) results on SSC18 were used as the gold standard for accuracy evaluation (Fig. 1 and Supplementary Table S2). Correlations (*R*^2^) [34] between genotypes and imputed dosages and genotypic concordance (GC) were calculated to evaluate the genotyping accuracy. The initial screening of SSC18 with BaseVar identified 506,452 and 414,160 bi-allelic candidate polymorphic sites before and after quality control, respectively. These sites were imputed using STITCH, and 322,386 SNPs were retained with a high mean call rate (98.89% ± 0.59%) after quality control (imputation info score > 0.4, Hardy Weinberg equilibrium *P*-value > 1e^−6^). The SNPs detected by BaseVar/STITCH were mostly included (99.32%) in the GATK-Beagle set, which included 570,919 sites and contained 320,199 SNPs overlapping with the BaseVar/STITCH dataset. As a result, a relatively high quality genotype set was acquired with less time consumption when *K* = 10 (the number of founders or ancestral haplotypes, Supplementary Fig. S2). Figure 2 shows that highly accurate genotypes were obtained using the BaseVar-STITCH pipeline compared with the high-depth sequencing result (*R*^2^ = 0.919 and GC = 0.970) across all allele frequencies, which exceeded the method using GATK-Beagle (*R*^2^ = 0.484 and GC = 0.709). Moreover, the BaseVar-STITCH results showed even higher GC concordance and *R*^2^ values compared with the GGP-80 data (*R*^2^ = 0.997 and GC = 0.990). Furthermore, direct genotyping (16 loci, 191 individuals) was carried out using the Fluidigm dynamic array IFC. The mean GC was 0.991 compared with the BaseVar-STITCH data (Supplementary Table S3), which is as high as the aforementioned results. Taken together, these results suggest that BaseVar-STITCH pipeline is a suitable variant discovery and imputation method for the LCS strategy (Fig. 1).

Previous studies have demonstrated that low-depth sequencing of a large number of samples generally provides a better representation of population genetic variations compared to high-depth sequencing of a limited number of individuals. In this study, we examined the consequences of altering the sample size and sequence coverage in this population. For the 0.5× coverage using STITCH, a sample size >500 had little effect on performance. At a 0.1× downsampled coverage, increasing the sample size to 1,985 led to a substantially improved performance (Fig. 2C and D). At 0.2× for 1,000 individuals, it was noteworthy that the results were only marginally poorer (*R*^2^ = 0.908 and GC = 0.962) than using all sequencing data (Fig. 2C and D). In general, the total sequencing depth (population category) for 1 locus >200× was shown to guarantee the credibility of genotyping within the scope of this study, although the results consistently improved as sequencing depth/sample size increased.

### Genetic architecture of the Duroc population

**Figure 3:**
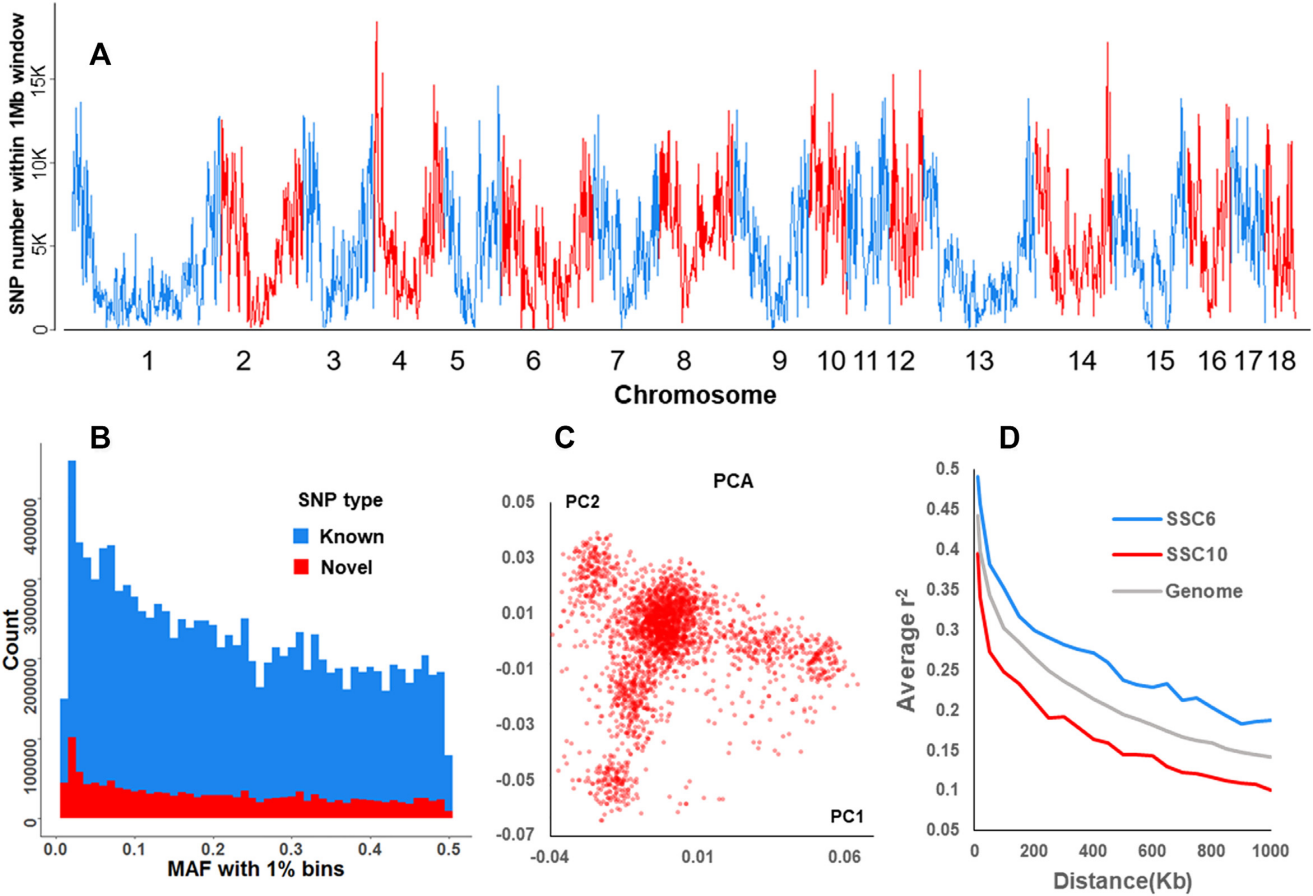
Genetic diversity of the Duroc population. (**A**) The distribution of SNPs in 1-Mb windows across the genome. (**B**) Histogram of allele counts by each 1% MAF bin. Novel (red) and known SNP sets (blue) were defined by comparing them to the pig dbSNP database. (**C**) Principal component 1 and 2 distribution in the Duroc population. (**D**) The extent of linkage disequilibrium (LD), in which the LD on chromosomes 6 (SSC6) and 10 (SSC10) represent the highest and lowest levels across the whole genome, respectively.

After strict parameter filtering in the pipeline (BaseVar-STITCH, Fig. 1), we retained 11,348,460 SNPs for all 2,797 Duroc pigs with high genotype accuracy, and the density corresponded to 1 SNP per 200 bp in the pig genome (Fig. 3A and Supplementary Table S4). Finally, the majority of the identified SNPs were located in intergenic regions (51.98%) and intronic regions (36.85%). The exonic regions contained 1.37% of the SNPs, including 0.14% missense SNPs. Among the discovered SNPs, 1,524,015 (accounting for 13.43% of all SNPs) were novel to the pig dbSNP database (data from NCBI: GCA_000003025.6 in June 2017). Both novel and known variants were found to have very similar minor allele frequency (MAF) distributions across the whole genome, with a mean MAF of 0.225 (Fig. 3B). A principal component analysis of all pigs showed that there was no distinct population stratification (Fig. 3C). The decay of LD with increasing distance was different among the chromosomes, of which the fastest and slowest decay rates occurred for SSC10 and SSC6, respectively. The mean pairwise LD *r*^2^ values decreased to 0.20 at 500 kb and to 0.14 at 1 Mb (Fig. 3D), providing the expected mapping resolution obtainable with this population.

Tajima D and diversity Pi were implemented to analyse selective sweep regions simultaneously, and only windows with an interquartile range of Tajima D and diversity Pi of 1.5-fold in the whole genome were regarded as putative selection regions. In total, 24 putative fixed selective regions harbouring 281 genes were identified (Supplementary Fig. S3). The regions displayed significant overrepresentation of genes involved in the sensory perception of smell (*P* = 6.41e^–10^) (Supplementary Table S5), reflecting the importance of smell when scavenging for food during long periods of environmental adaptation. This result is consistent with a previous study that reported that genes associated with olfaction exhibit fast evolution in pigs [35]. We also observed a significant enrichment of genes involved in the neurological system process (*P* = 8.64e^–5^), hair cycle process (*P* = 0.004), and bone mineralization (*P* = 0.040).

### GWAS and identification of high-resolution mapping of QTLs

**Figure 4:**
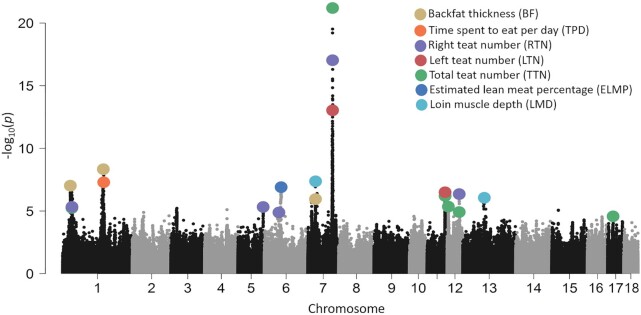
Summary Manhattan plot of 7 phenotypes with significant SNPs. Genome-wide representation of all quantitative trait loci (QTLs) identified in this study. Light and dark grey dots show associations from the 7 measures where ≥1 QTL was detected at the tagging SNP positions (n = 258,662). The most significant SNP positions at each QTL are marked with a colour dot.

The 21 phenotypes used in this study are described in Table 1. There was high correlation between traits of the same type (such as LMD, LMA, and LMP; BH, BL, and CC, Supplementary Table S6). We identified a subset of 258,662 SNPs that tagged all other SNPs with MAF >1% at LD *r*^2^ <0.98 for the first round of GWAS (Supplementary Table S4). Fine-mapping was performed within 10 Mb of the SNPs to reach a 5 genome-wide false discovery rate (FDR) significance threshold of 5%. Overall, we discovered 14 non-overlapping quantitative trait loci (QTLs) for the 7 traits at a significance threshold of 5% (Fig. 4, Table 1, Supplementary Figs S4 and S5). The widths of all QTL intervals ranged from ∼66 kb to ∼3.9 Mb. The intervals of 5 QTLs were >2 Mb in width (Supplementary Table S7). These QTLs were strongly influenced by the local LD levels of this population.

**Figure 5:**
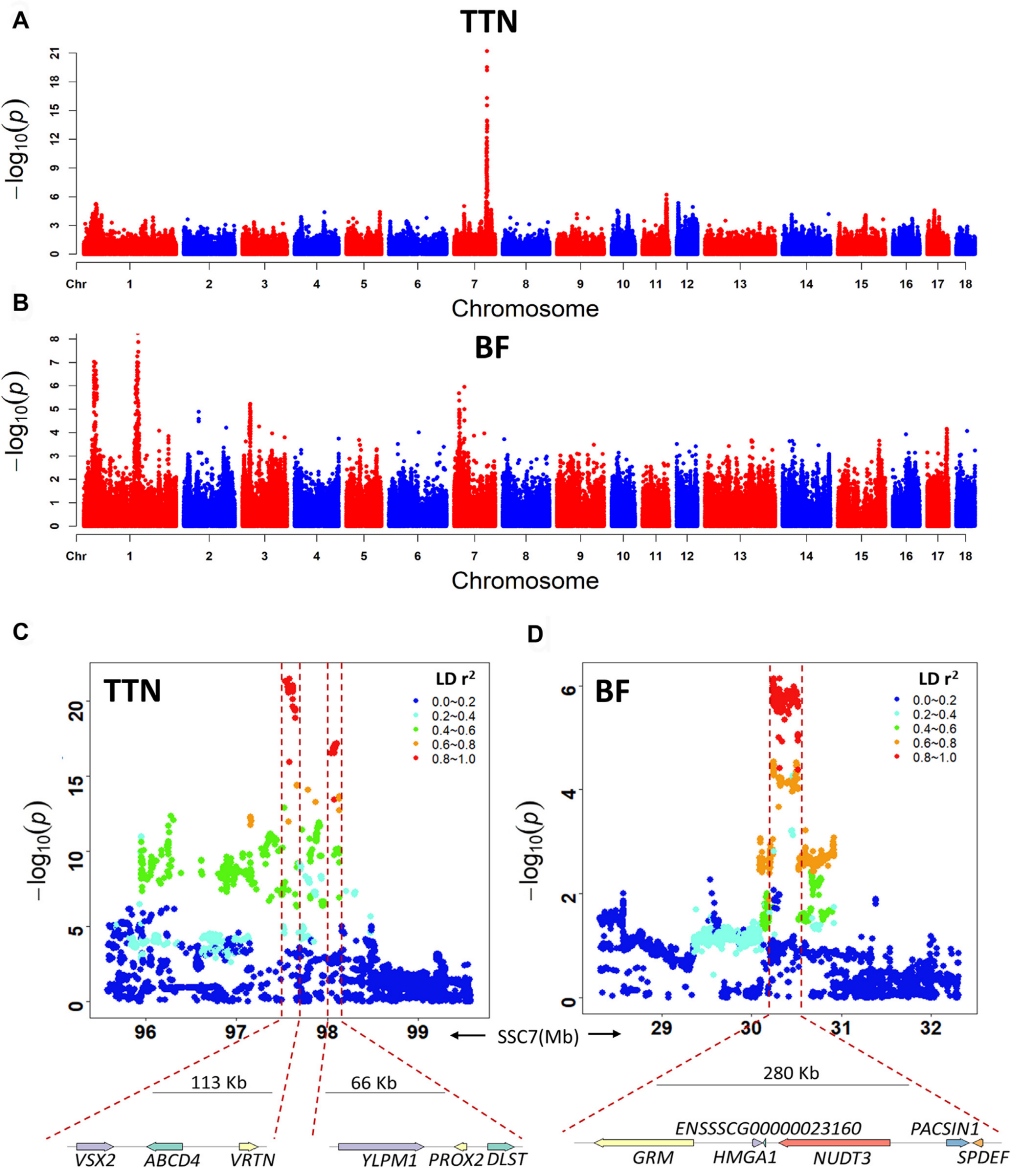
Manhattan plots and fine-mapping of the total teat number (TTN) and back fat thickness (BF). (**A**) and (**B**) Depict the TTN and BF association signals on the whole genome. (**C**) Fine-mapping of the TTN using the entire set of SNPs, in which 2 isolated regions on chromosome 7 with lengths of 113 and 66 kb were detected as QTLs. (**D**) Fine-mapping of BF using the entire set of SNPs. A narrow QTL with a length of 280 kb was detected on chromosome 7. The association genes within QTLs are displayed below.

On average, individual QTLs covered 13 protein-coding genes (ranging from 0 to 48) with a median of 8 genes. The distribution of the number of genes in a QTL is shown in Supplementary Table S7. We first focused on QTLs that could be narrowed because these loci could provide a starting point for functional investigations. Of the 14 non-overlapping loci identified in this study, 7 QTLs could be further narrowed to a small number of genes (1–9 genes) (Fig. 5 and Supplementary Fig. S6). Here, we highlight 2 important QTLs on SSC7.

The QTL on SSC7 with a major effect on the total teat number (TTN) has been widely identified in several commercial breeding lines and hybrids [36–38]. Our GWAS results show a strong QTL for TTN in the same region, explaining most of the phenotypic variance compared with other QTLs (Supplementary Table S7), reflecting the major effect of this locus (Fig. 4). Fine-mapping revealed 2 narrow LD blocks (SSC7:97.56–97.65 and 98.06–98.10 Mb), containing 4 candidate genes (*ABCD4, VRTN, PROX2*, and *DLST*) (Fig. 5 and Supplementary Fig. S6). We noticed that the most significant locus (SSC7: 97,581,669 bp, *P* = 3.29e^–22^) was detected in the region of the *ABCD4* gene, and 1 missense SNP in *ABCD4* had the most severe impact, with the largest decrease in protein stability (Supplementary Table S9), suggesting that *ABCD4* may be the most likely causal gene. In addition, 4 missense variants were discovered in *PROX2*, which was the vertebrate homolog of the homeodomain-containing protein, Prospero, that may be involved in cell fate determination and body plan establishment in *Drosophila melanogaster* [39]. Previous studies have reported that *PROX2* could be the causal gene [31, 40].

For the carcass traits, we identified 6 QTLs (Table 1 and Supplementary Table S7), in which a common narrowed QTL region on SSC7 of 30.24–30.52 Mb was identified to be significantly associated with back fat thickness (BF) and loin muscle depth (LMD) (Fig. 5 and Supplementary Fig. S6). Among the QTLs associated with BF and LMD, the narrowed QTL on SSC7 was found to make the greatest contribution to heritability, indicating that this was the location of the major genes in the region (Table 1 and Fig. 5). In this region (Supplementary Table S7), *HMGA1* is a promising candidate gene associated with growth, carcass, organ weight, and fat metabolism because it has been reported to be involved in a variety of genetic pathways regulating cell growth and differentiation, glucose uptake, and white and brown adipogenesis [41–45].

### Heritability and pattern of QTL effects

**Figure 6:**
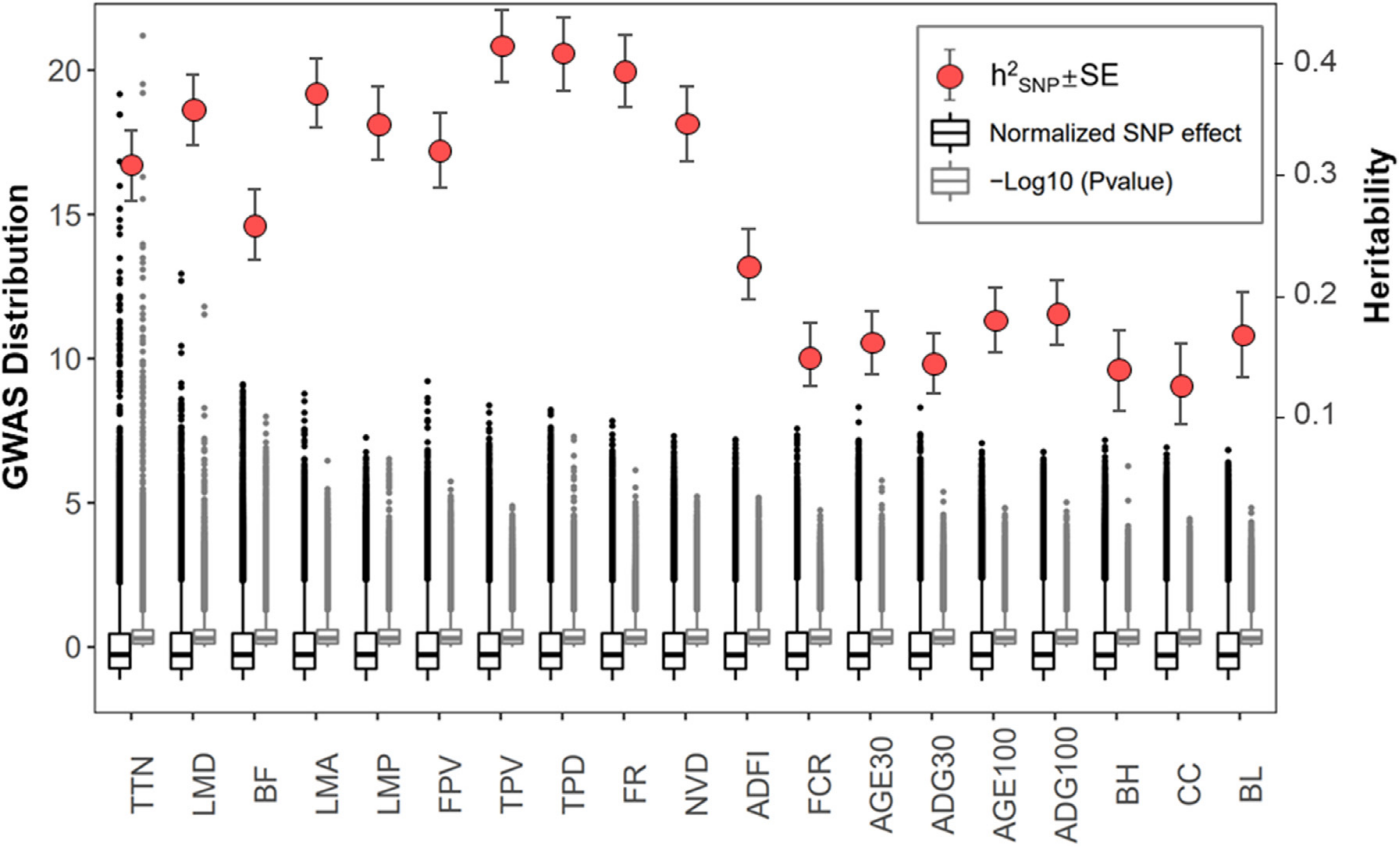
Heritability and SNP significance and normalized effect of 21 traits. The SNP effect was estimated and normalized and is displayed in the black boxplot. The grey boxplot represents the distribution of −log_10_  *P*-values for all SNPs. Red dots represrent heritability estimates, while black lines represent standard error. The boxes represent interquartile range (IQR), the whiskers represent 1.5 times IQR and individual points are outliers.

To assess how much of the heritability can be explained by the detected QTLs, we estimated the effect size of the overall decreased proportion of heritability by using significant SNPs distributed in these QTLs as fixed effects. Seven of the 21 traits (TTN, LTN, RTN, BF, LMD, LMP, and TPD) exhibited medium to high heritability–major QTL effect (1.08–8.86%) profile (Table 1 and Fig. 6). Among them, TTN showed the highest single-QTL effect and the most discrete distribution. The other 6 traits were explained by multiple QTLs, but the total effect was significantly lower than that of TTN. These results showed the differential genetic architecture of the gradual transition from qualitative-like traits to quantitative traits.

Few QTLs were detected for other traits, and most of them could be attributed to the typically small effect sizes of individual mutations, thousands of which contribute to the total observed genetic variation but did not reach the significant level for a typical complex trait (such as body size measurement and feed intake traits). It is noteworthy that the heritability of growth traits, such as the average daily gain 30–100 kg (ADG100) and age to 100 kg daily weight (AGE100) were lower than those of other populations [46, 47] which in turn resulted in no significant QTL. To account for this, we hypothesized that the major QTL effect may be obscured by rare mutations under strong artificial selection. We searched the candidate loci of growth traits in the pig QTL database [48] as well as the corresponding previous reports [46, 49–52]. We identified 51 sites associated with growth traits distributed on 18 chromosomes with low MAF (<0.05) in our population. However, 151 previously reported candidate sites were not identified as polymorphism in this study (Supplementary Table S8). The sequencing depths of these sites exceeded 2,100×, proving that these sites were completely fixed in our population with the same alleles as in the reference genome. This result reflected the long-term artificial selection history for growth traits of this commercial Duroc population and explained the decreased heritability and major QTLs.

## Discussion

To our knowledge, we have generated the largest WGS genotyping dataset for the Duroc population to date, containing 11 million markers from 2,797 pigs. We expanded the candidate causal mutations for multiple pig traits, and demonstrated the efficacy of genetic fine-mapping utilizing LCS in animal populations without reference panels. Furthermore, we compared the heritability and inheritance models for each trait, providing a starting point for functional investigations. Our study indicated that the LCS method could have widespread use in high-resolution GWAS for any genetic or breeding population, or even for applications in genomic prediction.

Our study identified an optimal design, taking into account the imputation algorithm, number of samples, and sequencing depth. The BaseVar-STITCH pipeline allows the GC to be >0.96 when the sample size is 1,000 at a sequencing depth of 0.2× (200× at the population level) without large reference panels. This GC value is significantly higher than that in other studies with small sample sizes with a high sequencing depth or array-based genotype imputation [7, 9]. We also found that genotype accuracy was more sensitive to the sample size than the sequencing depth. Hence, the results demonstrated that low-coverage designs are more powerful than deep sequencing of fewer individuals for animal sequencing studies because a large sample size can cover all local haplotypes of the study population more effectively. This method has high accuracy, even in large-scale human studies with the most complex population structure [33], further showing that a sufficient sample size will ensure that the method has a broad spectrum of applicability in all agricultural species or breeding populations.

Increasing marker density has been proposed to have the potential to improve the power of GWAS and the accuracy of genomic selection for quantitative traits [53]. First, the whole-genome LCS data gave the best accuracy for GWAS because they can capture more recombination events than SNP chips or target sequencing methods such as genotyping by sequencing [31], and most causal or causal-linked mutations that underlie a trait are expected to be included. Second, many studies have reported the impact of WGS data on the accuracy of genomic predictions [53–55]; however, the conclusions have been quite divergent. The limited improvement of the genetic relationship matrices for WGS data compared with the SNP chip is the major reason for the lack of improvement in genomic prediction. In addition, while most researchers may prefer to impute SNP chip genotypes using limited WGS data, some erroneous SNPs may be introduced and further adversely affect the performance of genomic prediction because limited haplotype architecture would be obtained using small-scale WGS data. Our method improved the accuracy of imputation, especially in large studies without a good reference panel and multibreed genomic predictions. Third, significantly improved genomic selection results were observed when SNPs were preselected from the sequenced data with prior information and an optimized genomic prediction method considering genomic features (e.g., GFBLUP [56, 57]). Thus, we could select different useful tag-SNPs for various traits with different genetic architectures using the high-density genetic map built by LCS data to optimize the genomic selection model in the future. Fourth, in practical applications, the haplotype reference panel can accommodate new haplotypes due to recombination at any time, thus solving the issue of a decrease in prediction accuracy over generations. Our data cover the sites of various SNP chips well because the genome coverage exceeds 98.36%, and the method is competitive with arrays in terms of cost and SNP density. In addition, we applied GTX, which is an FPGA-based hardware accelerator platform [58], to perform the alignments, and ∼3,000 alignments were accomplished in 2 days. Then, genotyping and imputation could be achieved on the cluster server or even on a cloud server in a single day, thus resolving the accuracy and timeliness of genomic prediction.

Recent swine breeding has prompted the accumulation of beneficial genetic variations at a more rapid rate, especially for some economically important trait loci [59, 60]. This study used a typical commercial population, which exhibits a high level of LD and number of selective regions under strong artificial selection. Thus, we presented a joint analysis of GWAS and selective sweep of this Duroc population to comprehensively extract more functional genes and genomic features. We detected 136 candidate genes (Supplementary Table S10) in 14 QTLs associated with 7 traits, and highlighted important roles, such as *ABCD4* for TTN and *HMGA1* for BF. A large number of fixed or nearly fixed loci have been found to be associated with ADG, AGE, and FCR, which explained the missing QTL by GWAS, and reflected the growth-related selection index process exactly. We also detected 24 putative fixed selective regions harbouring a series of genes enriched for sensory perception and neurological system processes. It has been widely reported that olfactory receptor genes may not only reflect adaptation to different environments [61] but might also act as a species barrier by affecting mate choice [62]. Several studies have reported an overrepresentation of genes with gene ontology (GO) terms related to neuronal development and neurological regulation [61, 63], which could be related to the complex genetic background of traits such as behaviour and increased tameness.

Fewer QTLs with significant SNPs were detected in feeding behaviour traits and body size measurements than in teat number and carcass traits. These observations are interpreted in a paradigm in which complex traits are driven by an accumulation of weak regulatory effects on the large genes and regulatory pathways [64–66], i.e., “infinitesimal model.” This model motivated us to aggregate hits to identify key pathways and processes. In particular, the feeding behaviour traits exhibited high heritability–few QTL effect profiles. We combined related genes obtained from the top 100 loci from the GWAS of the 6 feed intake traits. Gene-set enrichment analysis based on the obtained 281 genes showed that neural development or neural activity–related functions, such as astrocyte differentiation (*P* = 8.61e^–5^), cognition (*P* = 0.002), learning (*P* = 0.002), and glial cell differentiation (*P* = 0.003), were significantly enriched (Supplementary Fig. S7 and Table S11). The KEGG pathway analysis also showed that the nervous system processes were significantly enriched (Supplementary Fig. S8 and Table S12), including the neurotrophin signaling pathway *(P* = 0.015) (Supplementary Fig. S9) and GABAergic synapse (*P* = 0.021). This finding suggests that pig feeding behaviour involves complex traits that are affected by the regulation of the nervous system, leading to the stimulation of appetite. The current breeding schedule of this commercial population has been successful, especially in terms of improving growth traits. The next stage should focus on the use of genomic selection strategies for “infinitesimal traits” with high heritability but no major QTL, such as feeding behaviour traits.

In conclusion, we developed a Tn5-based, highly accurate, cost- and time-efficient LCS method to obtain whole-genome SNP markers in a large Duroc population. GWAS results for 21 important agricultural traits identified tens of important QTLs/genes and showed their various genetic architectures, providing promising guidance for further genetic improvement harnessing genomic features.

### Potential implications

The present work advances our understanding of the genetic architecture of quantitative traits and suggests a direction for future application of genomic information in pig breeding. We expect that our method could be applied to large-scale genome studies for any species without a good reference panel, especially for agricultural species that have important economic value. The rapid accumulation of data will significantly improve many bottlenecks in current genome research and will combine multi-omics information and artificial intelligence algorithms to contribute to decipher the genetic and regulatory mechanisms behind complex traits.

## Methods

### Animals, phenotyping, and DNA extraction

The Duroc boars used for this study were born from September 2011 to September 2013. All boars were managed on a single nucleus farm in a commercial company, which underwent strong artificial selection for many years. The associated phenotype data used in this study included back fat thickness at 100 kg (BF), loin muscle area at 100 kg (LMA), loin muscle depth at 100 kg (LMD), lean meat percentage at 100 kg (LMP), average daily gain (0–30 kg and 30–100 kg) (ADG30 and ADG100), age to 30 and 100 kg daily weight (AGE30 and AGE100), body length (BL), body height (BH), circumference of cannon bone (CC), feed conversion ratio (FCR), average daily feed intake (ADFI), number of visits to feeder per day (NVD), time spent eating per day (TPD), time spent eating per visit (TPV), feed intake per visit (FPV), feed intake rate (FR), left teat number (LTN), right teat number (RTN), and total teat number (TTN). The phenotype TTN data were acquired from the study of Tan et al. [31]. In detail, the number of left and right teats of each pig were recorded within 48 h after birth, and only normal teats were counted. The TTN in this study was the sum of normal left and right teats. Body weights were recorded at birth and at the beginning (30 ± 5 kg) and the end (100 ± 5 kg) of the experiment. The ADG was calculated as the total weight gain over this time, divided by the number of days. The ages at which the pig reached 30 and 100 kg were recorded as AGE30 and AGE100, respectively. BF, LMD, LMA, and LMP were measured over the last 3–4 ribs using b-ultrasound-scan equipment when the weight of pigs reached 100 ± 5 kg (Aloka SSD-500). Feeding behaviours including the time taken, duration, feed consumption, and weight of each pig were recorded at every visit by the Osborne FIRE Pig Performance Testing System (Osborne, KS, USA). The ADFI of each animal was obtained by dividing the total feed intake during the test by the number of days of the test period. The following feeding behaviour and eating efficiency traits were defined and calculated for each boar: ADFI (kg/day), TPD (min), NVD, TPV ( = TPD/NVD, %), FPV (kg), FR ( = DFI/TPD, g/min), and FCR ( = ADFI/ADG). The phenotypic values nearly all followed a normal distribution (Supplementary Fig. S1).

Genomic DNA was extracted from the ear tissue using a DNeasy Blood & Tissue Kit (Qiagen 69506), assessed using a NanoDrop, and checked in 1% agarose gel. All samples were quantified using a Qubit 2.0 Fluorometer and then diluted to 40 ng/mL in 96-well plates.

### Tn5 Library generation and sequencing

Equal amounts of Tn5ME-A/Tn5MErev and Tn5ME-B/Tn5MErev were incubated at 72°C for 2 minutes and then placed on ice immediately. Tn5 (Karolinska Institute, Sweden) was loaded with Tn5ME-A+rev and Tn5ME-B+rev in 2× Tn5 dialysis buffer at 25°C for 2 h. All linker oligonucleotides were the same as in a previous report [67].

Tagmentation was carried out at 55°C for 10 minutes by mixing 4 μL 5×TAPS-MgCl_2_, 2 μL of dimethylformamide (DMF) (Sigma Aldrich), 1 μL of the Tn5 pre-diluted to 16.5 ng/μL, 50 ng of DNA, and nuclease-free water. The total volume of the reaction was 20 μL. Then, 3.5 μL of 0.2% sodium dodecyl sulfate was added, and Tn5 was inactivated for another 10 min at 55°C.

KAPA HiFi HotStart ReadyMix (Roche) was used for PCR amplification. The primers were designed for MGI sequencers, with the reverse primers containing 96 different index adaptors to distinguish individual libraries. The PCR program was as follows: 9 min at 72°C, 30 sec at 98°C, and then 9 cycles of 30 sec at 98°C, 30 sec at 63°C, followed by 3 min at 72°C. The products were quantified by Qubit Fluorometric Quantitation (Invitrogen). The groups of 96 indexed samples were pooled with equal amounts (Supplementary Table S13).

Size selection was performed using AMPure XP beads (Beckmann), with a left side size selection ratio of 0.55× and a right side size selection ratio of 0.1×. The final libraries were sequenced on 2 lanes of MGISEQ-2000 to generate 2×100 bp paired-end reads or on 1 lane of BGISEQ-500 to generate 2×100 bp paired-end reads.

### Genotype data obtained using high-depth sequencing and SNP chip

We sequenced 37 of the total 2,869 pigs using the HiSeq X Ten system at a high depth of 15.15×. GTX by the Genetalks company, a commercially available FPGA-based hardware accelerator platform, was used in this study for both mapping clean reads to the Sscrofa11.1 reference genome [68] and variant calling. The alignment process was accelerated by FPGA implementation of a parallel seed-and-extend approach based on the Smith–Waterman algorithm, while the variant calling process was accelerated by FPGA implementation of GATK HaplotypeCaller (PairHMM) [69]. GATK multi-sample best practice was used to call and genotype SNPs for the 37 pigs, and the SNPs were hard filtered with a relatively strict option “QD < 10.0 || ReadPosRankSum < -8.0 || FS > 10.0 || MQ<40.0.”

We also selected 42 individuals who were included in the LCS dataset and genotyped using the GeneSeek Genomic Profiler Porcine 80K SNP Array and obtained 68,528 SNPs across the whole genome. The genotypes of the sex chromosomes were excluded from this study, and after quality control (genotype call rate > 0.95), 47,946 SNPs remained. We retained 45,308 SNPs that overlapped with the LCS dataset to evaluate the genotypes from the LCS strategy.

### Low-coverage sequencing data analyses

Sequencing reads from the low-coverage samples were mapped to the Sscrofa11.1 reference genome using GTX-align, which includes a step that involves marking PCR duplicates. The indel realignment and base quality recalibration modules in GATK were applied to realign the reads around indel candidate loci and to recalibrate the base quality. The mean running time from a fastq file to a bam file was ∼3 min for each sample in this study. Variant calling was done using the BaseVar and hard filtered with Estimated allele frequency (EAF) ≥ 0.01 and a depth ≥1.5 times the interquartile range. The detailed BaseVar algorithm that was used to call SNP variants and estimate allele frequency was described in a previous report [33]. We used STITCH to impute genotype probabilities for all individuals. The key parameter K (number of ancestral haplotypes) was decided on the basis of the tests in SSC18. Results were filtered with an imputation info score > 0.4 and a Hardy Weinberg equilibrium *P*-value > 1e^−6^. After quality control, 2,797 individuals with genotype data were obtained. Two validation actions were taken to calculate the accuracy of imputation. The first parameter was GC, which was calculated as the number of correctly imputed genotypes divided by the total number of sites. Another parameter was the allele dosage *R*^2^, which was described in a previous report [34]. The SNPEff program [70] was used to annotate the variants.

### Population genetics analysis

A subset of 258,662 SNPs that tagged all other SNPs with MAF > 1% at LD *r*^2^ < 0.98 and a call rate of >95% were retained for downstream analysis. Principal component analysis clustering analyses were performed using the GCTA software [71]. The mean heterozygosity rate and MAF were obtained using the vcftools program [72]. Tajima D [73] and diversity Pi were implemented to analyse selective sweep regions simultaneously with the window size set to 1 Mb, and only windows with an interquartile range for Tajima D and diversity Pi of 1.5-fold in the whole genome were regarded as putative selection regions. The GO terms were downloaded from the Ensembl website using the BioMart tool [74], and the KEGG pathway was obtained according to the NCBI gene accession number, and both GO and KEGG terms were organism specific (*Sus scrofa*). Finally, annotations of 335,522 GO terms and 6,139 KEGG pathways were retained for enrichment analyses. Both enrichment analyses were performed using the OmicShare tools [75], and the significance was determined by the *P*-value according to the hypergeometric test (*P* < 0.05).

### Genome-wide association and heritability estimation

A mixed linear model approach was used for the genome-wide association analyses based on tagging SNPs, as implemented in the GCTA package [71]. The statistical model included the year and month as discrete covariates. For BF, LMA, LMD, and LMP, the year and season were included as discrete covariates, and the weights at the beginning and end of the test were used as quantitative covariates. To correct for multiple testing across the genome, the FDR correction obtained using FDRtool R package [76] was applied to determine the genome-wide significance threshold (FDR < 0.05). Once a QTL had been mapped using the tagging SNPs and exceeded the FDR threshold, association was recalculated with all imputed SNPs (from the 11.3 M set) in a 20-Mb window around the peak using the same mixed model. The definition of a fine-mapped candidate QTL interval was based on the LD level between the most significant SNP and all flanking sites, where the boundary was verified when the LD was ≤0.8. The SNP effect was estimated using the GREML_CE program in the GVCBLUP package [77], where the result was absoluted and normalized.

Heritability was estimated using a mixed model as follows:

\begin{equation*} {{\bf y}} = {{{\bf X}}_{{\bf b}}}{{\bf b}} + {{\bf Za}} + {{\bf e}} \end{equation*}

with Var(**y**) = **ZA**_a_**Z**′ơ_a_^2^  **+ I**ơ_e_^2^, where **Z** is an incidence matrix allocating phenotypic observations to each animal; **b** is the vector of the fixed year-month effects for BF, LMA, LMD, and LMP that also includes the weights at the beginning and end of the test as covariance; **X_b_**is the incidence matrix for **b; a** is the vector of additive values based on the genotype data; **A**_a_ is a genomic additive relationship matrix; ơ_a_^2^ is the additive variance; and ơ_e_^2^ is the residual variance. Variance components were estimated by genomic restricted maximum likelihood estimation (GREML) using the GREML_CE program in the GVCBLUP package. The additive heritability was defined as *h*_a_^2^ = ơ_a_^2^/(ơ_a_^2^ + ơ_e_^2^). SNP effects were defined by the GREML_CE program and then normalized using R script.

The heritability of the detected QTL was estimated as follows:

\begin{equation*} {{\bf y}} = {{\bf X}}{^\prime_{{\bf b}}}{{\bf b}}^\prime + {{\bf Za}} + {{\bf e}} \end{equation*}

with Var(**y**) = **ZA**_a_**Z′**ơ_a_^2^  **+ I**ơ_e_^2^, where **Z** is an incidence matrix allocating phenotypic observations to each animal; **b′** is the vector of the fixed year-month effects and significant SNPs identified in the QTL region using GWAS analysis for BF, LMA, LMD, and LMP; **b** also includes the weights at the beginning and end of the test as covariance; **X′_b_** is the incidence matrix for **b; a** is the vector of additive values based on the genotype data; **A**_a_ is a genomic additive relationship matrix; ơ_a_^2^ is the additive variance; and ơ_e_^2^ is the residual variance. The QTL heritability was defined as *h*_qtl_^2^ = *h*_a_^2^–ơ_a_^2^/(ơ_a_^2^ + ơ_e_^2^).

### Functional consequence of the missense mutations associated with TN

The effect of the missense SNPs associated with TN on the stability of pig ABCD4, PROX2, and DLST proteins was assessed using I-Mutant adaptation 2.0 [78]. A potential surge or reduction in the DDG was predicted, along with a reliability index, where the lowest and highest reliability levels were 0 and 10, respectively.

### Direct genotyping by Fluidigm IFC technology

Sixteen loci on SSC7 were selected on the basis of the GWAS results, 3 of which were related to BF, while the others were related to TN. Primers for genotyping were designed and ordered on the Fluidigm D3 assay design website (Supplementary Table S13), and 191 out of the total 2,869 pigs were genotyped for each SNP using Fluidigm Dynamic array IFC.

## Data Availability

All of the sequencing raw data in this study have been deposited into NCBI and can be accessed via accession Nos. PRJNA681437 and PRJNA712489. Scripts, VCF files, phenotype information for 7 traits, and other supporting data are available via the GigaScience repository, GigaDB [79]. The individual index information of the LCS dataset is listed in Supplementary Table S13.

## Additional Files

**Supplementary Figure S1:** Phenotypic distribution of 21 traits.

**Supplementary Figure S2:** Dosage *R*^2^ and cost time (minutes) among different K values. Accuracy and cost time of genotyping from K = 5 to K = 25, where the blue and black lines represent the dosage *R*^2^ and cost time (minutes), respectively.

**Supplementary Figure 3:** Purifying selection regions in the whole genome. Purifying selection signals were detected on SSC2, SSC3, SSC6, SSC7, SSC9, and SSC15, where blue and red lines represent –log_10_ Pi and Tajima D, respectively, and the grey regions depict the purifying selected regions.

**Supplementary Figure 4:** Manhattan plots of phenotypes with no significant SNPs. Manhattan plots of ADFT, NVD, TPV, FPV, FR, FCR, BH, BL, CC, ADG100, AGE100, ADG30, AGE30, and LMA, where no significant SNPs were detected in these traits.

**Supplementary Figure 5:** QQ plot of 21 phenotypes.

**Supplementary Figure 6:** Summary plots of fine mapping.

**Supplementary Figure 7:** Distribution of top 100 SNPs based on *P*-value using GWAS analysis.

**Supplementary Figure 8:** GO and KEGG enrichment of genes identified to be associated with feeding behaviour traits.

**Supplementary Figure 9:** Neurotrophin signalling pathway enrichment. The red tangles represent detected pathways in this study, which including Bcl-2, NT3, TrkB, and p75NTF.

**Supplementary Table S1:** LCS data set.

**Supplementary Table S2:** Resequencing Duroc samples list.

**Supplementary Table S3:** Genotypic concordance between BaseVar-STITCH method and direct genotyping by Fluidigm IFC technology.

**Supplementary Table S4:** Number and density of SNPs imputed by STITCH and Tag SNP.

**Supplementary Table S5:** GO enrichment of genes located in the selected regions.

**Supplementary Table S6:** Genetic and phenotypic coefficient of 21 traits.

**Supplementary Table S7:** Summary of detected QTLs.

**Supplementary Table S8:** Summary table of markers identified significantly associated with ADG, AGE, or FCR in previous studies.

**Supplementary Table S9:** Missense SNPs in the narrowed QTL region of TN.

**Supplementary Table S10:** Gathered information of candidate genes.

**Supplementary Table S11:** GO enrichment of genes located in the selected regions.

**Supplementary Table S12:** KEGG enrichment of genes located in the selected regions.

**Supplementary Table S13:** Index sequence for the all LCS samples.

**Supplementary Table S14:** Primers used for Fluidigm IFC genotyping.

giab048_GIGA-D-20-00354_Original_Submission

giab048_GIGA-D-20-00354_R2

giab048_GIGA-D-20-00354_Revision_1

giab048_Response_to_Reviewer_Comments_Original_Submission

giab048_Response_to_Reviewer_Comments_Revision_1

giab048_Reviewer_1_Report_Original_Submission Samuele Bovo, Ph.D. -- 1/7/2021 Reviewed

giab048_Reviewer_1_Report_Revision_1 Samuele Bovo, Ph.D. -- 4/15/2021 Reviewed

giab048_Reviewer_2_Report_Original_Submission Martien Groenen -- 1/18/2021 Reviewed

giab048_Reviewer_2_Report_Revision_1 Martien Groenen -- 4/16/2021 Reviewed

giab048_Supplemental_Figures_and_Tables

## Abbreviations

ADFI: average daily feed intake; ADG30: average daily gain (0–30 kg); ADG100: average daily gain (30–100 kg); AGE30: age to 30 kg live weight; AGE100: age to 100 kg live weight; BF: back fat thickness at 100 kg; BH: body height; BL: body length; bp: base pairs; CC: circumference of cannon bone; FCR: feed conversion rate; FDR: false discovery rate; FPV: feed intake per visit; FR: feed intake rate; GATK: Genome Analysis Toolkit; GC: genotypic concordance; GO: Gene Ontology; GWAS: genome-wide association studies; IFC: Integrated Fluidic Circuit; kb: kilobase pairs; KEGG: Kyoto Encyclopedia of Genes and Genomes; LCS: low-coverage sequencing method; LD: linkage disequilibrium; LMA: loin muscle area at 100 kg; LMD: loin muscle depth at 100 kg; LMP: lean meat percentage at 100 kg; LTN: left teat number; MAF: minor allele frequency: Mb: megabase pairs; NCBI: National Center for Biotechnology Information; NVD: number of visits to feeder per day; QTL: quantitative trait locus; RTN: right teat number; SNP: single-nucleotide polymorphism; TPD: time spent eating per day; TPV: time spent eating per visit; TTN: total teat number; WGS: whole-genome sequencing.

## Competing Interests

The authors declare that they have no competing interests.

## Funding

This study is supported by the financial support of the National Transgenic Grand Project[2016ZX08009003-006], the 948 Program of the Ministry of Agriculture of China (2012-G1(4)), the Science and Technology Innovation Strategy Projects of Guangdong Province [2019B020203002], and the Guangdong Academician Workstation [2011A090700016].

## Authors’ Contributions

X.H., N.L.: conceptualization. X.H., N.L., Y.W., and Z.W.: project administration and supervision. X.H., Y.W., D.Z., X.G., J.R., Z.H., C.B., and R.Y.: methodology, investigation, and formal analysis. R.Y., D.Z., X.G., and Y.W.: data curation and validation. Z.W., G.C., D.L., and C.T.: resources. X.H. and Z.W.: funding acquisition. Y.W. and R.Y.: visualization and original draft preparation. Y.W., X.H., Y.Z., G.C., and D.L.: review and editing.

## References

[1] Visscher PM, Brown MA, McCarthy MI, et al. Five years of GWAS discovery. Am J Hum Genet. 2012;90(1):7–24. 22243964 10.1016/j.ajhg.2011.11.029 PMC3257326

[2] Huang X, Wei X, Sang T, et al. Genome-wide association studies of 14 agronomic traits in rice landraces. Nat Genet. 2010;42(11):961–7. 20972439 10.1038/ng.695

[3] Marchini J, Howie B. Genotype imputation for genome-wide association studies. Nat Rev Genet. 2010;11(7):499–511. 20517342 10.1038/nrg2796

[4] Marchini J, Howie B, Myers S, et al. A new multipoint method for genome-wide association studies by imputation of genotypes. Nat Genet. 2007;39(7):906–13. 17572673 10.1038/ng2088

[5] Howie BN, Donnelly P, Marchini J. A flexible and accurate genotype imputation method for the next generation of genome-wide association studies. PLoS Genet. 2009;5(6):e1000529. 19543373 10.1371/journal.pgen.1000529 PMC2689936

[6] Howie B, Fuchsberger C, Stephens M, et al. Fast and accurate genotype imputation in genome-wide association studies through pre-phasing. Nat Genet. 2012;44(8):955. 22820512 10.1038/ng.2354 PMC3696580

[7] Yan G, Qiao R, Zhang F, et al. Imputation-based whole-genome sequence association study rediscovered the missing QTL for lumbar number in Sutai pigs. Sci Rep. 2017;7(1):615. 28377593 10.1038/s41598-017-00729-0 PMC5429657

[8] Van Binsbergen R, Bink MC, Calus MP, et al. Accuracy of imputation to whole-genome sequence data in Holstein Friesian cattle. Genet Sel Evol. 2014;46(1):41. 25022768 10.1186/1297-9686-46-41 PMC4226983

[9] Van den Berg S, Vandenplas J, van Eeuwijk FA, et al. Imputation to whole-genome sequence using multiple pig populations and its use in genome-wide association studies. Genet Sel Evol. 2019;51:2. 30678638 10.1186/s12711-019-0445-y PMC6346588

[10] Swarts K, Li HH, Navarro JAR, et al. Novel methods to optimize genotypic imputation for low-coverage, next-generation sequence data in crop plants. Plant Genome. 2014;7(3):doi:10.3835/plantgenome2014.05.0023.

[11] Buerkle CA, Gompert Z. Population genomics based on low coverage sequencing: how low should we go?. Mol Ecol. 2013;22(11):3028–35. 23174005 10.1111/mec.12105

[12] Huang L, Wang B, Chen RT, et al. Reveel: large-scale population genotyping using low-coverage sequencing data. Bioinformatics. 2016;32(11):1686–96. 26353840 10.1093/bioinformatics/btv530

[13] Li Y, Sidore C, Kang HM, et al. Low-coverage sequencing: implications for design of complex trait association studies. Genome Res. 2011;21(6):940–51. 21460063 10.1101/gr.117259.110 PMC3106327

[14] Gilly A, Southam L, Suveges D, et al. Very low-depth whole-genome sequencing in complex trait association studies. Bioinformatics. 2019;35(15):2555–61. 30576415 10.1093/bioinformatics/bty1032 PMC6662288

[15] Davies RW, Flint J, Myers S, et al. Rapid genotype imputation from sequence without reference panels. Nat Genet. 2016;48(8):965. 27376236 10.1038/ng.3594 PMC4966640

[16] Ros-Freixedes R, Gonen S, Gorjanc G, et al. A method for allocating low-coverage sequencing resources by targeting haplotypes rather than individuals. Genet Sel Evol. 2017;49(1):78. 29070022 10.1186/s12711-017-0353-y PMC5655873

[17] Fragoso CA, Heffelfinger C, Zhao HY, et al. Imputing genotypes in biallelic populations from low-coverage sequence data. Genetics. 2016;202(2):487. 26715670 10.1534/genetics.115.182071 PMC4788230

[18] Bickhart DM, Hutchison JL, Null DJ, et al. Reducing animal sequencing redundancy by preferentially selecting animals with low-frequency haplotypes. J Dairy Sci. 2016;99(7):5526–34. 27085415 10.3168/jds.2015-10347

[19] Zan Y, Payen T, Lillie M, et al. Genotyping by low-coverage whole-genome sequencing in intercross pedigrees from outbred founders: a cost-efficient approach. Genet Sel Evol. 2019;51(1):44. 31412777 10.1186/s12711-019-0487-1 PMC6694510

[20] Nicod J, Davies RW, Cai N, et al. Genome-wide association of multiple complex traits in outbred mice by ultra-low-coverage sequencing. Nat Genet. 2016;48(8):912–8. 27376238 10.1038/ng.3595 PMC4966644

[21] 1000 Genomes Project Consortium, Abecasis GR, Altshuler D, Auton A, et al. A map of human genome variation from population-scale sequencing. Nature. 2010;467(7319):1061–73. 20981092 10.1038/nature09534 PMC3042601

[22] GenomeAsia100K Consortium. The GenomeAsia 100K Project enables genetic discoveries across Asia. Nature. 2019;576(7785):106–11. 31802016 10.1038/s41586-019-1793-z PMC7054211

[23] Wang Q, Pierce-Hoffman E, Cummings BB, et al. Landscape of multi-nucleotide variants in 125,748 human exomes and 15,708 genomes. Nat Commun. 2020;11(1):2539. 32461613 10.1038/s41467-019-12438-5 PMC7253413

[24] Genome of the Netherlands Consortium. Whole-genome sequence variation, population structure and demographic history of the Dutch population. Nat Genet. 2014;46(8):818–25. 24974849 10.1038/ng.3021

[25] Gudbjartsson DF, Sulem P, Helgason H, et al. Sequence variants from whole genome sequencing a large group of Icelanders. Sci Data. 2015;2:150011. 25977816 10.1038/sdata.2015.11 PMC4413226

[26] Lam HM, Xu X, Liu X, et al. Resequencing of 31 wild and cultivated soybean genomes identifies patterns of genetic diversity and selection. Nat Genet. 2010;42(12):1053–9. 21076406 10.1038/ng.715

[27] Daetwyler HD, Capitan A, Pausch H, et al. Whole-genome sequencing of 234 bulls facilitates mapping of monogenic and complex traits in cattle. Nat Genet. 2014;46(8):858–65. 25017103 10.1038/ng.3034

[28] Hayes BJ, Daetwyler HD. 1000 bull genomes project to map simple and complex genetic traits in cattle: applications and outcomes. Annu Rev Anim Biosci. 2019;7(1):89–102. 30508490 10.1146/annurev-animal-020518-115024

[29] Yang J, Benyamin B, McEvoy BP, et al. Common SNPs explain a large proportion of the heritability for human height. Nat Genet. 2010;42(7):565–9. 20562875 10.1038/ng.608 PMC3232052

[30] Lango Allen H, Estrada K, Lettre G, et al. Hundreds of variants clustered in genomic loci and biological pathways affect human height. Nature. 2010;467(7317):832–8. 20881960 10.1038/nature09410 PMC2955183

[31] Tan C, Wu ZF, Ren JL, et al. Genome-wide association study and accuracy of genomic prediction for teat number in Duroc pigs using genotyping-by-sequencing. Genet Sel Evol. 2017;49(1):35. 28356075 10.1186/s12711-017-0311-8 PMC5371258

[32] Ros-Freixedes R, Battagin M, Johnsson M, et al. Impact of index hopping and bias towards the reference allele on accuracy of genotype calls from low-coverage sequencing. Genet Sel Evol. 2018;50(1):64. 30545283 10.1186/s12711-018-0436-4 PMC6293637

[33] Liu S, Huang S, Chen F, et al. Genomic analyses from non-invasive prenatal testing reveal genetic associations, patterns of viral infections, and Chinese population history. Cell. 2018;175(2):347–59.e14. 30290141 10.1016/j.cell.2018.08.016

[34] Browning BL, Browning SR. A unified approach to genotype imputation and haplotype-phase inference for large data sets of trios and unrelated individuals. Am J Hum Genet. 2009;84(2):210–23. 19200528 10.1016/j.ajhg.2009.01.005 PMC2668004

[35] Paudel Y, Madsen O, Megens HJ, et al. Copy number variation in the speciation of pigs: a possible prominent role for olfactory receptors. BMC Genomics. 2015;16(1):330. 25896665 10.1186/s12864-015-1449-9 PMC4413995

[36] Zhuang Z, Ding R, Peng L, et al. Genome-wide association analyses identify known and novel loci for teat number in Duroc pigs using single-locus and multi-locus models. BMC Genomics. 2020;21(1):344. 32380955 10.1186/s12864-020-6742-6 PMC7204245

[37] Van Son M, Lopes MS, Martell HJ, et al. A QTL for number of teats shows breed specific effects on number of vertebrae in pigs: bridging the gap between molecular and quantitative genetics. Front Genet. 2019;10:272. 30972109 10.3389/fgene.2019.00272 PMC6445065

[38] Moscatelli G, Dall'Olio S, Bovo S, et al. Genome-wide association studies for the number of teats and teat asymmetry patterns in Large White pigs. Anim Genet. 2020;51(4):595–600. 32363597 10.1111/age.12947

[39] Pistocchi A, Bartesaghi S, Cotelli F, et al. Identification and expression pattern of zebrafish prox2 during embryonic development. Dev Dyn. 2008;237(12):3916–20. 19035352 10.1002/dvdy.21798

[40] Ren DR, Ren J, Ruan GF, et al. Mapping and fine mapping of quantitative trait loci for the number of vertebrae in a White Duroc x Chinese Erhualian intercross resource population. Anim Genet. 2012;43(5):545–51. 22497517 10.1111/j.1365-2052.2011.02313.x

[41] Gong H, Xiao S, Li W, et al. Unravelling the genetic loci for growth and carcass traits in Chinese Bamaxiang pigs based on a 1.4 million SNP array. J Anim Breed Genet. 2019;136(1):3–14. 30417949 10.1111/jbg.12365

[42] Liu X, Wang LG, Liang J, et al. Genome-wide association study for certain carcass traits and organ weights in a large White×Minzhu intercross porcine population. J Integr Agr. 2014;13(12):2721–30.

[43] Arce-Cerezo A, Garcia M, Rodriguez-Nuevo A, et al. HMGA1 overexpression in adipose tissue impairs adipogenesis and prevents diet-induced obesity and insulin resistance. Sci Rep. 2015;5(1):14487. 26411793 10.1038/srep14487 PMC4585969

[44] Wang LG, Zhang LC, Yan H, et al. Genome-wide association studies identify the loci for 5 exterior traits in a Large White x Minzhu pig population. PLoS One. 2014;9(8):e103766. 25090094 10.1371/journal.pone.0103766 PMC4121205

[45] Ji JX, Yan GR, Chen D, et al. An association study using imputed whole-genome sequence data identifies novel significant loci for growth-related traits in a Duroc × Erhualian F_2_ population. J Anim Breed Genet. 2019;136(3):217–28. 30869175 10.1111/jbg.12389

[46] Tang Z, Xu J, Yin L, et al. Genome-wide association study reveals candidate genes for growth relevant traits in pigs. Front Genet. 2019;10:302. 31024621 10.3389/fgene.2019.00302 PMC6459934

[47] Hoque MA, Kadowaki H, Shibata T, et al. Genetic parameters for measures of residual feed intake and growth traits in seven generations of Duroc pigs. Livest Sci. 2009;121(1):45–9.

[48] https://www.animalgenome.org/cgi-bin/QTLdb/SS/index. Accessed: 1 June 2020.

[49] Fontanesi L, Schiavo G, Galimberti G, et al. A genomewide association study for average daily gain in Italian Large White pigs. J Anim Sci. 2014;92(4):1385–94. 24663154 10.2527/jas.2013-7059

[50] Silva EF, Lopes MS, Lopes PS, et al. A genome-wide association study for feed efficiency-related traits in a crossbred pig population. Animal. 2019;13(11):2447–56. 31133085 10.1017/S1751731119000910

[51] Qiao R, Gao J, Zhang Z, et al. Genome-wide association analyses reveal significant loci and strong candidate genes for growth and fatness traits in two pig populations. Genet Sel Evol. 2015;47(1):17. 25885760 10.1186/s12711-015-0089-5 PMC4358731

[52] Ding R, Yang M, Wang X, et al. Genetic architecture of feeding behavior and feed efficiency in a Duroc pig population. Front Genet. 2018;9:220. 29971093 10.3389/fgene.2018.00220 PMC6018414

[53] Meuwissen T, Goddard M. Accurate prediction of genetic values for complex traits by whole-genome resequencing. Genetics. 2010;185(2):623–31. 20308278 10.1534/genetics.110.116590 PMC2881142

[54] Zhang C, Kemp RA, Stothard P, et al. Genomic evaluation of feed efficiency component traits in Duroc pigs using 80K, 650K and whole-genome sequence variants. Genet Sel Evol. 2018;50(1):14. 29625549 10.1186/s12711-018-0387-9 PMC5889553

[55] Yan G, Guo T, Xiao S, et al. Imputation-based whole-genome sequence association study reveals constant and novel loci for hematological traits in a large-scale swine F2 resource population. Front Genet. 2018;9:401. 30405681 10.3389/fgene.2018.00401 PMC6204663

[56] Edwards SM, Sorensen IF, Sarup P, et al. Genomic prediction for quantitative traits is improved by mapping variants to gene ontology categories in *Drosophila melanogaster*. Genetics. 2016;203(4):1871–83. 27235308 10.1534/genetics.116.187161 PMC4981283

[57] Xiang R, Berg IVD, MacLeod IM, et al. Quantifying the contribution of sequence variants with regulatory and evolutionary significance to 34 bovine complex traits. Proc Natl Acad Sci U S A. 2019;116(39):19398–408. 31501319 10.1073/pnas.1904159116 PMC6765237

[58] Xing Y, Li G, Wang Z, et al. GTZ: a fast compression and cloud transmission tool optimized for FASTQ files. BMC Bioinformatics. 2017;18(S16):549. 29297296 10.1186/s12859-017-1973-5 PMC5751770

[59] Bosse M, Megens HJ, Frantz LA, et al. Genomic analysis reveals selection for Asian genes in European pigs following human-mediated introgression. Nat Commun. 2014;5(1):4392. 25025832 10.1038/ncomms5392 PMC4225517

[60] Bosse M, Lopes MS, Madsen O, et al. Artificial selection on introduced Asian haplotypes shaped the genetic architecture in European commercial pigs. Proc Biol Sci. 2015;282(1821):20152019. 26702043 10.1098/rspb.2015.2019 PMC4707752

[61] Groenen MA, Archibald AL, Uenishi H, et al. Analyses of pig genomes provide insight into porcine demography and evolution. Nature. 2012;491(7424):393–8. 23151582 10.1038/nature11622 PMC3566564

[62] Hoover KC . Smell with inspiration: the evolutionary significance of olfaction. Am J Phys Anthropol. 2010;143(S51):63–74. 21086527 10.1002/ajpa.21441

[63] Carneiro M, Rubin CJ, Di Palma F, et al. Rabbit genome analysis reveals a polygenic basis for phenotypic change during domestication. Science. 2014;345(6200):1074–9. 25170157 10.1126/science.1253714 PMC5421586

[64] Boyle EA, Li YI, Pritchard JK. An expanded view of complex traits: from polygenic to omnigenic. Cell. 2017;169(7):1177–86. 28622505 10.1016/j.cell.2017.05.038 PMC5536862

[65] Wang Y, Cao X, Luo C, et al. Multiple ancestral haplotypes harboring regulatory mutations cumulatively contribute to a QTL affecting chicken growth traits. Commun Biol. 2020;3(1):472. 32859973 10.1038/s42003-020-01199-3 PMC7455696

[66] Chakravarti A, Turner TN. Revealing rate-limiting steps in complex disease biology: the crucial importance of studying rare, extreme-phenotype families. Bioessays. 2016;38(6):578–86. 27062178 10.1002/bies.201500203

[67] Picelli S, Bjorklund AK, Reinius B, et al. Tn5 transposase and tagmentation procedures for massively scaled sequencing projects. Genome Res. 2014;24(12):2033–40. 25079858 10.1101/gr.177881.114 PMC4248319

[68] ftp://ftp.ensembl.org/pub/release-99/fasta/sus_scrofa/dna/ .

[69] McKenna A, Hanna M, Banks E, et al. The Genome Analysis Toolkit: a MapReduce framework for analyzing next-generation DNA sequencing data. Genome Res. 2010;20(9):1297–303. 20644199 10.1101/gr.107524.110 PMC2928508

[70] Cingolani P, Platts A, Wang LL, et al. A program for annotating and predicting the effects of single nucleotide polymorphisms, SnpEff: SNPs in the genome of *Drosophila melanogaster* strain w1118; iso-2; iso-3. Fly (Austin). 2012;6(2):80–92. 22728672 10.4161/fly.19695 PMC3679285

[71] Yang J, Lee SH, Goddard ME, et al. GCTA: a tool for genome-wide complex trait analysis. Am J Hum Genet. 2011;88(1):76–82. 21167468 10.1016/j.ajhg.2010.11.011 PMC3014363

[72] Danecek P, Auton A, Abecasis G, et al. The variant call format and VCFtools. Bioinformatics. 2011;27(15):2156–8. 21653522 10.1093/bioinformatics/btr330 PMC3137218

[73] Tajima F . Statistical method for testing the neutral mutation hypothesis by DNA polymorphism. Genetics. 1989;123(3):585–95. 2513255 10.1093/genetics/123.3.585 PMC1203831

[74] http://asia.ensembl.org/biomart/martview/. Accessed: 5 May, 2020.

[75] http://www.omicshare.com/tools. Accessed: 6 April, 2020.

[76] Strimmer K . fdrtool: a versatile R package for estimating local and tail area-based false discovery rates. Bioinformatics. 2008;24(12):1461–2. 18441000 10.1093/bioinformatics/btn209

[77] Wang C, Prakapenka D, Wang S, et al. GVCBLUP: a computer package for genomic prediction and variance component estimation of additive and dominance effects. BMC Bioinformatics. 2014;15(1):270. 25107495 10.1186/1471-2105-15-270 PMC4133608

[78] Capriotti E, Calabrese R, Casadio R. Predicting the insurgence of human genetic diseases associated to single point protein mutations with support vector machines and evolutionary information. Bioinformatics. 2006;22(22):2729–34. 16895930 10.1093/bioinformatics/btl423

[79] Yang R, Guo X, Zhu D, et al. Supporting data for “Accelerated deciphering of the genetic architecture of agricultural economic traits in pigs using a low-coverage whole-genome sequencing strategy.”. GigaScience Database. 2021. 10.5524/100894. PMC8290195 34282453

