# Supplementary material for: Accelerated deciphering of the genetic architecture of agricultural economic traits in pigs using a low-coverage whole-genome sequencing strategy
Source: Gigascience. 2021 Jul 20;10(7):giab048. doi: 10.1093/gigascience/giab048 (PMC8290195; doi:10.1093/gigascience/giab048)
Supplement: giab048_GIGA-D-20-00354_Original_Submission [file giab048_giga-d-20-00354_original_submission.pdf]

GigaScience

Accelerated Deciphering of the Genetic Architecture of Agricultural Economic Traits in Pigs Using the Low Coverage Whole-genome Sequencing Strategy

--Manuscript Draft--

Manuscript Number:	GIGA-D-20-00354
Full Title:	Accelerated Deciphering of the Genetic Architecture of Agricultural Economic Traits in Pigs Using the Low Coverage Whole-genome Sequencing Strategy
Article Type:	Research

Funding Information:	
National Transgenic Grand Project (2016ZX08009003-006)	Prof. Xiaoxiang Hu
948 Program of the Ministry of Agriculture of China (2012-G1(4))	Prof. Xiaoxiang Hu
Science and Technology Innovation Strategy Projects of Guangdong Province (2019B020203002)	Prof. Xiaoxiang Hu
Guangdong Academician Workstation (2011A090700016)	Prof. Ning Li

Abstract:

Background: Uncovering the genetic architecture of economic traits in pigs is important for agricultural breeding. Two difficulties limiting the genetic analysis of complex traits are the unavailability of high-density markers for large population in most agricultural species which are lack of good reference panel, and the association signals tend to be spread across most of the genome, i.e., the infinitesimal model of quantitative traits. Findings: Here, we discovered a Tn5-based highly accurate, cost- and time-efficient, low coverage sequencing (LCS) method to obtain whole genome markers and performed whole-genome sequencing on 2,869 Duroc boars at an average depth of 0.73× to identify 11.3 M SNPs. Based on these SNPs, the genome-wide association study (GWAS) detected 14 quantitative trait loci (QTLs) in 7 of 21 important agricultural traits in pigs and provided a starting point for further investigation such as ABCD4 for total teat number and HMGA1 for back fat thickness. The inheritance models of different traits were found to vary greatly. Most obey the minor-polygene model but can be attributed to different reasons, such as the shaping of genetic architecture by artificial selection for this population and sufficiently interconnected minor gene regulatory networks. Conclusions: GWAS results for 21 important agricultural traits identified tens of important QTLs/genes and showed their various genetic architectures, providing promising guidance for genetic improvement harnessing genomic feature. The Tn5-based LCS method can be applied to large-scale genome studies for any species without good reference panel and widely used for agricultural breeding.

Corresponding Author:	Xiaoxiang Hu<br>China Agricultural University<br>CHINA
Corresponding Author Secondary Information:	
Corresponding Author's Institution:	China Agricultural University
Corresponding Author's Secondary Institution:	
First Author:	Ruifei Yang, Ph.D.
First Author Secondary Information:	
Order of Authors:	Ruifei Yang, Ph.D.
	Xiaoli Guo
	Di Zhu
	Cheng Tan, Ph.D.

Powered by Editorial Manager® and ProduXion Manager® from Aries Systems Corporation

	Cheng Bian
	Jiangli Ren
	Zhuolin Huang
	Yiqiang Zhao, Ph.D.
	Gengyuan Cai, Ph.D.
	Dewu Liu, Ph.D.
	Zhenfang Wu, Ph.D.
	Yuzhe Wang, Ph.D.
	Ning Li, Ph.D.
	Xiaoxiang Hu, Ph.D.
**Order of Authors Secondary Information:**	
**Additional Information:**	
**Question**	**Response**
Are you submitting this manuscript to a special series or article collection?	No
**Experimental design and statistics**<br><br>Full details of the experimental design and statistical methods used should be given in the Methods section, as detailed in our Minimum Standards Reporting Checklist. Information essential to interpreting the data presented should be made available in the figure legends.<br><br>Have you included all the information requested in your manuscript?	Yes
**Resources**<br><br>A description of all resources used, including antibodies, cell lines, animals and software tools, with enough information to allow them to be uniquely identified, should be included in the Methods section. Authors are strongly encouraged to cite Research Resource Identifiers (RRIDs) for antibodies, model organisms and tools, where possible.<br><br>Have you included the information requested as detailed in our Minimum Standards Reporting Checklist?	Yes

Powered by Editorial Manager® and ProduXion Manager® from Aries Systems Corporation

**Availability of data and materials**<br><br>All datasets and code on which the conclusions of the paper rely must be either included in your submission or deposited in publicly available repositories (where available and ethically appropriate), referencing such data using a unique identifier in the references and in the "Availability of Data and Materials" section of your manuscript.<br><br>Have you have met the above requirement as detailed in our Minimum Standards Reporting Checklist?	Yes

Manuscript

Click here to access/download;Manuscript;Manuscript-GIGA.docx

Accelerated Deciphering of the Genetic Architecture of Agricultural Economic Traits in Pigs Using the Low Coverage Whole-genome Sequencing Strategy

Ruifei Yang[1,2†], Xiaoli Guo[1†], Di Zhu[1†], Cheng Tan[3], Cheng Bian[1], Jiangli Ren[1], Zhuolin Huang[1], Yiqiang Zhao[1], Gengyuan Cai[3], Dewu Liu[3], Zhenfang Wu[3]*, Yuzhe Wang[1,2]*, Ning Li[1] and Xiaoxiang Hu[1]*

[1]State Key Laboratory of Agrobiotechnology, College of Biological Sciences, China Agricultural University, Beijing, China.

[2]College of Animal Science and Technology, China Agricultural University, Beijing, China.

[3]National Engineering Research Center for Breeding Swine Industry, South China Agricultural University, Guangdong, China.

Correspondence address: State Key Laboratory of Agrobiotechnology, College of Biological Sciences, China Agricultural University, Beijing, 100193, P. R. China. Tel: ++86-010-62733394; E-mail: huxx@cau.edu.cn, yuzhe891@163.com, wzfemail@163.com.

†These authors have contributed equally and should be considered co-first authors

Abstract

Background: Uncovering the genetic architecture of economic traits in pigs is important for agricultural breeding. Two difficulties limiting the genetic analysis of complex traits are the unavailability of high-density markers for large population in most agricultural species which are lack of good reference panel, and the association signals tend to be spread across most of the genome, i.e., the infinitesimal model of quantitative traits. Findings: Here, we discovered a Tn5-based highly accurate, cost- and time-efficient, low coverage sequencing (LCS) method to obtain whole genome markers and performed whole-genome sequencing on 2,869 Duroc boars at an average depth of 0.73× to identify 11.3 M SNPs. Based on these SNPs, the genome-wide association study (GWAS) detected 14 quantitative trait loci (QTLs) in 7 of 21 important agricultural traits in pigs and provided a starting point for further investigation such as *ABCD4* for total teat number and *HMGA1* for back fat thickness. The inheritance models of different traits were found to vary greatly. Most obey the minor-polygene model but can be attributed to different reasons, such as the shaping of genetic architecture by artificial selection for this population and sufficiently interconnected minor gene regulatory networks. Conclusions: GWAS results for 21 important agricultural traits identified tens of important QTLs/genes and showed their various genetic architectures, providing promising guidance for genetic improvement harnessing genomic feature. The Tn5-based LCS method can be applied to large-scale genome studies for any species without good reference panel and widely used for agricultural breeding.

KEYWORDS: Low coverage sequencing; GWAS; Genotyping; Pig; Genetic architecture; Agricultural traits

Introduction

Genome-wide association studies (GWAS) have identified thousands of genetic variants associated with complex traits in humans and agricultural species [1, 2]. The mapping resolution lies on the density of genetic markers which perceive linkage disequilibrium (LD) in sufficiently large populations [3, 4]. Despite the declining cost of sequencing, it is still expensive for agricultural breeding studies to apply whole-genome sequencing to all individuals in a large cohort (thousands of levels). In many scenarios, imputation-based strategies, which impute low-density panels to higher densities, offer an alternative to systematic genotyping or sequencing [5, 6]. To date, array-based genotype imputation has been widely used in agricultural species [7, 8]. The imputation accuracy of this strategy crucially depends on the reference panel sizes and genetic distances between the reference and target populations. However, the unavailability of large reference panels and array designs for target populations in agricultural species limits the improvement of array-based genotype imputation [9, 10]. Inaccurate imputations influence the results of follow-up population genetic analyses.

In terms of recently developed methods, low-coverage sequencing (LCS) of a large cohort has been proposed to be more informative than sequencing fewer individuals at a higher coverage rate [11-13]. Sample sizes and haplotype diversity could be more critical than sequencing depth in determining the genotype accuracy of most segregating sites and increasing the power of association studies. Overall, LCS has been proven to have greater power for trait mapping compared to the array-based genotyping method in human studies [14]. To date, LCS-based genotype imputation has been employed in many studies using various populations and genotyping algorithms [15-19]. In particular, the STITCH imputation algorithm overcomes the barrier of the lack of good reference panels in non-human species and is even applicable in studies with extremely low sequencing depths [15, 20]. This is a promising approach for agricultural animals without large reference panels and can be used in the areas of functional genetic mapping and genomic breeding. However, thus far, no reports on this field have been done.

Several large-scale whole-genome sequencing projects have been completed [21]. These projects were designed to identify the underlying mechanisms that drive hereditary diseases in human as well as for use in genomic selection in the breeding of agricultural species [22-24]. The infinitesimal model, which describe the inheritance patterns of human quantitative traits appears to be successful [25, 26]; however, it is unclear how many genes play important roles in driving different kinds of complex traits. In addition, artificial selection provides a driving force to make agricultural species evolve fast, which further brings about the fixation of selection regions and differentials in the inheritance model. This process might process a very different result for the same trait between studies due to different genetic backgrounds of the research population. Therefore, care should be taken when determining the GWAS result for a specific population. Such information which might be helpful for understanding the genetic mechanism for a complex trait and could be informative for further application of genomic selection in animal breeding.

In this study, we developed a new highly accurate, cost- and time-efficient LCS method to obtain high-density SNP markers for a large Duroc population [27]. By assessing 21 important agricultural traits in commercial pig herds, we performed genome-wide association and fine-mapping analyses with high resolution and compared the results of the inheritance model in depth. We also proved that artificial selection plays a significant role in altering the genetic architecture of agricultural animals, especially for those loci that affect economic traits. The LCS strategy provides a powerful method for further agricultural breeding.

Results

Genome sequencing and data acquisition

A Tn5-based protocol was used to prepare sequencing libraries of each pig at a low cost (reagent cost: $2.60 /library) as described in the Materials and Methods section. The libraries were sequenced on the Illumina (PE 150 model, 2 libraries) and the BGI platform (PE 100 model, 28 libraries) (Supplementary Table S1). The results generated

by the BGI platform had lower PCR duplicates (2.23%), higher good index reads (97.10%), and higher genome coverage (98.55%) than the Illumina dataset (10.82% PCR duplicates, 93.64% good index reads, and 98.50% genome coverage). Overall, the total output of the 2,869 boars approached 5.32 TB, and the majority (96.74%) of reads were successfully mapped to the pig reference genome Sscrofa11.1. Each animal was sequenced at an average of depth of 0.73±0.17×. Moreover, both high depth resequencing (average 15.15×/sample) and SNP Array (GeneSeek Genomic Profiler Porcine 80K SNP Array, GGP-80) genotyping were done on the selected Duroc core boars of this population, and the results were used for downstream accuracy evaluation.

Processing pipeline of the low-coverage strategy and accuracy evaluation

Traditional standard methods for SNP calling, such as those implemented in GATK and Samtools, were mainly used in high-depth resequencing methods. However, due to the low depth of each base, erroneous SNPs and genotypes could be called using such methods, especially for the GATK HaplotypeCaller algorithm (single sample local de novo assembly) [28]. In this study, we applied the BaseVar algorithm [29] to identify polymorphic sites and infer allele frequencies, and we used STITCH [15] to impute SNPs. We first used chromosome 18 (SSC18) to test the BaseVar-STITCH and GATK (UnifiedGenotypeCaller)-Beagle algorithms with genotypes from 1,985 pigs. The 37 verified individuals were genotyped by GATK best practice using HaplotypeCaller for 15.15× sequencing data (Fig. 1 and Supplementary Table S2). Correlations (R^2) [30] between genotypes and imputed dosages and the genotypic concordance (GC) were calculated to evaluate the genotyping accuracy. The initial screening of SSC18 with BaseVar identified 506,452 and 414,160 bi-allelic candidate polymorphic sites before and after quality control, respectively. These sites were imputed using STITCH, and 322,386 SNPs were retained with a high average call rate (98.89% ± 0.59%) after quality control (imputation info score > 0.4 and Hardy Weinberg Equilibrium *P* value > $1e^{-6}$). The SNPs detected by BaseVar/STITCH were mostly included (99.32%) in the GATK-Beagle set, which included 570,919 sites and contained 320,199 SNPs overlapping with the BaseVar/STITCH dataset. As a result, a relatively high-quality

genotype set was acquired with less time consumption when K = 10 (the number of founders or ancestral haplotypes, Fig. S1). Fig. 2 shows that highly accurate genotypes were obtained using the BaseVar-STITCH pipeline (R^2 = 0.919 and GC = 0.970) across all allele frequencies, which excelled far beyond the method using GATK-Beagle (R^2 = 0.484 and GC = 0.709). Moreover, we also compared BaseVar-STITCH results with the genotypes in GGP-80. The results showed even higher GC concordance and R^2 values (R^2 = 0.997 and GC = 0.990) when all 2,797 samples were used, which further validated BaseVar-STITCH with a high level of confidence. Therefore, we conclude that the BaseVar-STITCH pipeline is a suitable variant discovery and imputation method for the LCS strategy (Fig. 1).

Previous studies have demonstrated that sequencing a large number of samples at a low depth generally provides a better representation of population genetic variations compared to sequencing a limited number of individuals at a higher depth. Here, we examined the consequences of altering the sample size and sequence coverage in this population. For the 0.5× coverage using STITCH, a sample size above 500 had little impact on performance, while at an 0.1× down-sampled coverage, increasing the sample size to 1,985 led to a substantially improved performance (Fig. 2C and 2D). At 0.2× for 1,000 individuals, it was noteworthy that the results were only marginally poorer (R^2 = 0.908 and GC = 0.962) than using all sequencing data (Fig. 2C and 2D). In general, the total sequencing depth (population category) for one locus > 200× was shown to guarantee the credibility of genotyping within the scope of this study, although the results did consistently improve as the sequencing depth/sample size increased.

Genetic architecture of the Duroc population

After strict parameter filtering in the pipeline (BaseVar-STITCH, Fig. 1), we retained 11,348,460 SNPs in all 2,797 Duroc pigs with high genotype accuracy, and the density corresponded to 1 SNP per 200 bp in the pig genome (Fig. 3A and Supplementary Table S3). Finally, the majority of identified SNPs were located in intergenic regions (51.98%) and intronic regions (36.85%). The exonic regions contained 1.37% of the SNPs, including 0.14% missense SNPs. Among the discovered SNPs, 1,524,015 (accounting

for 13.43% of all SNPs) were novel to the pig dbSNP database (data from NCBI: GCA_000003025.6 on June, 2017). Both novel and known variants were found to have very similar minor allele frequency distributions across the whole genome and the average minor allele frequency (MAF) was 0.225 (Fig. 3B). A principal component analysis (PCA) of all pigs showed that there was no distinct population stratification (Fig. 3C). The decay of LD with increasing distance was different among the chromosomes, of which the fastest and slowest decay rates occurred for SSC10 and SSC6, respectively. Average pairwise LD r^2 values fell to 0.20 at 500 Kb and to 0.14 at 1 Mb (Fig. 3D), providing an indication of the expected mapping resolution obtainable with this population.

We further studied the high level of LD and found that it could be a consequence of long-term strong natural or artificial selection. Tajima's D and diversity Pi was implemented to analyze selective sweep regions simultaneously and only windows with an interquartile range of Tajima's D and diversity Pi of 1.5-fold in the whole genome were regarded as putative selection regions. In total, 24 putative fixed selective regions harboring 281 genes were obtained (Fig. S2). The regions displayed significant overrepresentation of genes involved in the sensory perception of smell (P = 6.41e^{-10}) (Supplementary Table S4), reflecting the importance of smell when scavenging for food during long periods of environmental adaptation. This result is consistent with a previous study that reported that genes associated with olfaction exhibit fast evolution in pigs. We also observed a significant enrichment of genes involved in the neurological system process (P = 8.64e^{-5}). These genes may be associated with behavior and increased tameness and thus were under selection during early domestication. In addition, the hair cycle process (P = 0.004) and bone mineralization (P = 0.040) were also detected to be significantly enriched, which may represent the phenotypic changes of coat and body composition during pig domestication.

GWAS and identification of high-resolution mapping of QTLs

The 21 associated phenotypes used in this study are shown in Table 1 and Fig. S3. We identified a subset of 258,662 SNPs that tagged all other SNPs with MAF >1% at LD

r^2 <0.98 for the first-round of GWAS (Supplementary Table S3). Fine-mapping was performed within 10 Mb of the SNPs to reach 5% FDR significance threshold genome-wide. Overall, we discovered a total of 14 non-overlapping QTLs for the seven traits at a significance threshold of 5% (Fig. 4, Table 1, Fig. S4, and Fig. S5). The widths of all QTL intervals ranged from ~66 Kb to ~3.9 Mb. The intervals of five QTLs were more than 2 Mb in width (Supplementary Table S5). These QTLs were strongly influenced by the local linkage disequilibrium level of this population.

On average, each QTL covered 13 protein-coding genes (range of 0–48) with a median of eight genes. The distribution of the number of genes in a QTL is shown in Supplementary Table S5. We first focused on QTLs that could be narrowed, since these loci could provide a starting point for functional investigations. Of the 14 non-overlapping loci identified in this study, seven QTLs could be further narrowed to a small number of genes (1 to 9 genes) (Fig. 5 and Fig. S6). Here, we highlight two important QTLs on SSC7.

The QTL on SSC7 with a major effect on the total teat number (TTN) has been widely identified in several commercial breeding lines and hybrids. Our GWAS results show a strong QTL for TTN in the same region, explaining most of the phenotypic variance compared with other QTLs (Supplementary Table S5), reflecting the major effect of this locus. (Fig. 4). Fine-mapping discovered two narrow LD blocks (SSC7:97.56–97.65 Mb and 98.06–98.10 Mb) containing four candidate genes (*ABCD4*, *VRTN*, *PROX2*, and *DLST*) (Fig. 5 and Fig. S6). It is worth noting that four missense variants were discovered in *PROX2*, one of which was the vertebrate homolog of the Drosophila melanogaster homeodomain-containing protein Prospero, which may be involved in the determination of cell fate and the establishment of the body plan [31], and former studies reported that *PROX2* could be the causal gene. Besides, although there is no direct evidence supporting the involvement of *ABCD4* in the development process of the mammary gland, we noticed that the most significant locus (SSC7:97,581,669, $P = 3.29e^{-22}$) was detected in the region of this gene, suggesting that *ABCD4* may be the most likely causal gene.

For the carcass traits, we identified six QTLs (Table 1 and Supplementary Table S5), in which a common narrowed QTL region on SSC7 of 30.24–30.52 Mb was identified to be significantly associated with back fat thickness (BF) and loin muscle depth (LMD) (Fig. 5 and Fig. S6). Among the QTLs associated with BF and LMD, the narrowed QTL on SSC7 was found to make the greatest contribution to the heritability, so this would be the location of the major genes in the region (Table 1 and Fig. 5). In this region (Supplementary Table S5), *HMGA1* is a promising candidate gene associated with growth, carcass, organ weights, and fat metabolism, as it has been reported to be involved in a variety of genetic pathways regulating cell growth and differentiation, glucose uptake, and white and brown adipogenesis [32-36]. *Nudt3* belongs to the Nudix hydrolase family, which is involved in diverse metabolic processes, including the regulation of important signaling nucleotides and their metabolites. It is an obesity-linked gene that is associated with insulin signaling and may be another candidate causal gene of BF and LMD. Other genes, including *PACSIN1* and *SPDEF*, have also been reported to be candidate genes with functions in glutathione metabolism, adipose and muscle tissue development, and lipid metabolism for LMD.

Heritability and pattern of QTL effects

To assess how much of the heritability can be explained by the detected QTLs, we estimated the effect size of the overall decreased proportion of heritability by using significant SNPs distributed in these QTLs as fixed effects. As reported in Table 1, we detected a larger number of contributions to heritability by major QTLs for the teat number (3.16~8.86), which indicates that the teat number is mainly controlled by a small number of loci. We also distributed the effects and significance (-Log_{10} P value) of SNPs for all 21 traits. Again, the result showed that TTN had the most discrete distribution (Fig. 6).

We detected six non-overlapping major QTLs for BF, LMD, and LMP, and the proportion of explained variation by these QTLs reached 1.19–2.40% which is lower than that for the teat number. The results reveal that although major QTLs are associated

with carcass traits, the effect is relatively limited and there could be a larger number of minor gene effects.

Few QTL were detected for other traits, and most of them could be attributed to the typically small effect sizes of individual mutations, thousands of which contribute to the total observed genetic variation for a typical complex trait (such as BH, body length (BL), and cannon bone (CC)). However, two types of interesting genetic architecture have caught our attention. In terms of the first one, previous studies reported that growth traits (such as the average daily gain 30-100 kg (ADG100) and age to 100 kg daily weight (AGE100)) all have medium or high heritability, and several QTLs have been detected. However, low heritability traits (ADG100:0.187, AGE100: 0.181) with no significant QTL were detected in this study. To account for this, we hypothesize that the major QTL effect may be obscured by rare mutations under strong artificial selection. We searched the candidate loci of growth traits in the pig QTL database (https://www.animalgenome.org/cgi-bin/QTLdb/SS/index) as well as corresponding previous reports and identified 51 sites associated with growth traits distributed on 18 chromosomes with a low minor allele frequency (MAF < 0.05) in our population. Moreover, 151 previously-reported candidate sites were not identified as SNPs in this study (Supplementary Table S6). We checked the sequencing depths of these sites, all of which exceeded 2,100×, proving that these sites were completely fixed in our population with the same alleles as in the reference genome. This result reflects the long-term artificial selection history of this commercial Duroc population for growth traits and also explains the lost heritability and major QTLs.

Second, for the feed intake traits (including average daily feed intake (ADFI), number of visits to feeder per day (NVD), time spent eating per day (TPD), time spent eating per visit (TPV), and feed intake per visit (FPV)), the heritability was at a medium or high level (Fig. 6) but we did not obtain any significant QTL (except one QTL for TPD). The results showed the distribution of SNP were scattered across the whole genome (Fig. S7) and the effects was more even (Fig. 6), suggesting that these traits are controlled by the regulatory effect of multiple minor genes and may have highly

complex interactions. In order to clarify the biological functions of these minor candidate genes, we combined related genes obtained from the top 100 loci from the GWAS of the six feed intake traits according to the GWAS analysis. The gene-set enrichment analysis based on the obtained 281 genes showed that neural development or neural activity related functions, such as astrocyte differentiation (P = 8.61e^{-5}), cognition (P = 0.002), learning (P = 0.002), and glial cell differentiation (P = 0.003), were significantly enriched (Fig. S7 and Supplementary Table S7). The KEGG pathway analysis also showed there were significantly enriched nervous system processes (Fig. S8 and Supplementary Table S8), including the neurotrophin signaling pathway (P = 0.015) and the GABAergic synapse (P = 0.021). This result shows that pig feeding behavior involves complex traits that are affected by the regulation of the nervous system, leading to the stimulation of appetite.

Discussion

To our knowledge, we have generated the largest WGS genotyping dataset for the Duroc population so far. It contains 11 million markers from 2,797 pigs. We expanded the candidate causal mutations for multiple pig traits and demonstrated the efficacy of genetic fine-mapping utilizing low-coverage sequencing in animal populations without reference panels. Further, we compared the heritability and inheritance model of each trait, providing a starting point for functional investigations. Our study indicates that the LC method could have widespread usage in high resolution genome-wide association studies for any genetic or breeding population or even for application in genomic prediction.

This study identified an optimal design, taking into account the imputation algorithm, the number of samples, and the sequencing depth. The BaseVar-STITCH pipeline allows the GC to be higher than 0.96 when the sample size is 1000 at a sequencing depth of 0.2× (200× at the population level) without large reference panels. This GC value is significantly higher than that found in other studies with small sample sizes with a high sequencing depth or array-based genotype imputation. We also found

that the genotype accuracy is more sensitive to the sample size than the sequencing depth. In other words, the results demonstrated that low-coverage designs are more powerful than the deep sequencing of fewer individuals for animal sequencing studies, since a large sample size can cover all local haplotypes of the study population more effectively. This method has amazing accuracy, even in large-scale human studies with the most complex population structure [29], which further shows that a sufficient sample size will ensure that the method has a broad spectrum of applicability in all agricultural species or any breeding population. Therefore, using the low-coverage sequencing strategy, we were able to consider both the high-density SNP map and a large population.

Increasing the marker density has been proposed to have the potential to improve the power of GWAS and the accuracy of genomic selection (GS) for quantitative traits [37]. First, the whole-genome low-coverage sequencing data gave the best accuracy for GWAS, since it can catch more recombinations than SNP chips or target sequencing methods such as genotyping by sequencing (GBS), and most causal or causal-linked mutations that underlie a trait are expected to be included. Second, lots of studies have reported the impact of whole genome sequence data on the accuracy of genomic predictions [37-39]; however, the conclusions have been quite divergent. The limited improvement of the genetic relationship matrices for WGS data compared with the SNP chip is the major reason for the lack of improvement in genomic prediction. In addition, most researchers may prefer to impute SNP chip genotypes using limited WGS data; however, some erroneous SNPs may be introduced and further adversely affect the performance of genomic prediction, since limited haplotype architecture would be obtained using small-scale WGS data. Our method improves the accuracy of imputation, especially in a large studies without a good reference panel and multibreed genomic predictions, which will make the application of genome selection wider. Third, significantly improved GS results have been observed when SNPs were preselected from the sequenced data using GWAS and a nonlinear genomic prediction method (*e.g.*, Bayes model [40] or TABLUP [41]). Thus, we could select different useful tag-SNPs

for various traits with different genetic architectures using the high-density genetic map built by LCS data to optimize the genomic selection model in the future. Fourth, in practical application, the haplotype reference panel can accommodate new haplotypes due to recombination at any time, thus solving the issue of a decrease in prediction accuracy over generations. Our data can cover the sites of various SNP chips well because the genome coverage exceeds 98.36%, and it is competitive with arrays in terms of the cost and SNP density. Last, we applied GTX, which is an FPGA-based hardware accelerator platform [42], to do the alignments, and ~3,000 alignments were accomplished in two days. Then, the genotyping and imputation could be achieved on the cluster server or even a cloud server in a single day, thus resolving the accuracy and timeliness issue for genomic prediction.

Previous studies demonstrated that pigs have differentiated into a variety of local populations due to environmental adaptation, and they were domesticated around ~10,000 years ago. Since then, natural and artificial selection have both contributed to the further speciation of pigs [43, 44]. Recent swine breeding has prompted the accumulation of beneficial genetic variations at a more rapid rate, especially for some economic trait loci [45, 46]. The purebred Duroc population studied in this research was selected for meat production mainly due to its growth-related index. A large number of fixed loci have been found to be associated with ADG, AGE, and FCR, which reflects this selection process exactly. We also detected 24 putative fixed selective regions. For example, in these regions, *MC5R* was detected to be a possible candidate gene for fatness in pigs [47], major QTLs for pig growth and carcass traits were identified to be centered in the regions of *OGN* and *ASPN* [48], and *AKIRIN1* was found to be involved in the regulation of muscle development by playing important roles in maintaining the muscle fiber type and regulating skeletal muscle metabolic activity [49]. *CRTC3* encodes a member of the CRTC protein family and plays an important role in energy metabolism [50, 51]. It was found to be associated with lipid accumulation in pigs [51]. In addition, a series of genes enriched for sensory perception and neurological system processes were also detected in selective regions. It has been

widely reported that olfactory receptor genes may not only reflect adaptation to different environments [43] but also might have acted as a species barrier by affecting mate choice [52]. Several studies have reported an overrepresentation of genes with GO (gene ontology) terms related to neuronal development and neurological regulation [43, 53], and this could be related to the complex genetic background of traits such as behavior and increased tameness. It should be noted that the results may be due to a mixture of natural and artificial selection causes. The complex genetic background and single population analyses may limit the precision of exploration of selection signatures exploration, so analyses of population genetics in multiple breeds in a large population and multi-omics may be needed.

In this study, we detected 14 non-overlapping QTLs in 7 of 21 traits (Table 1 and Supplementary Table S5). There were big differences of loci and QTL effects among these traits, which may represent the inheritance models of different traits, including phenotypes that are mainly affected by several major genes (teat number) or multiple minor genes (such as carcass traits). Above all, seven non-overlapped QTLs with narrowed intervals were identified, which emphasizes the potential for identifying new mutations in QTLs using the low-coverage sequencing method. Some candidate genes may reside within these regions. For the teat number, we first focused on the QTL interval on SSC7 which explained most of the phenotypic variance. It should be noted that six missense SNPs were identified to be extremely significant (Supplementary Table S9). We estimated the effects of the variants and found one located in *ABCD4* had the most severe impact with the largest decrease of protein stability. Moreover, the most extremely significant locus was located in the intron region of *ABCD4* ($P = 3.29e^{-22}$). We therefore suggest that *ABCD4* is one of the most promising causal genes affecting the teat number in pigs. For the carcass traits (LMD and BF), several candidate genes were detected in the narrowed QTLs, especially for the QTL with major effects on SSC7, including *Grm4*, *Hmga1*, *NUDT3*, R*PS10*, *PACSIN1*, and *SPDEF*, which have also been widely identified. Moreover, there were three QTLs that had not been identified in previous studies as far as we know: those detected in TTN

(SSC1:34,657,653-36,881,340), LMP (SSC13:83,054,253-84,673,400), and TPD (SSC1:157,891,084-161,827,351). For TTN and LMP, the newly discovered QTLs explained the limited phenotypic variance, indicating the minor effects of these loci. For TPD, we noted that the same QTL was also identified in BF, which suggests that the intervals may contain genes that control appetite. Apart from the QTLs identified in high-resolution discussed above, we also detected several loci, though their intervals could not be narrowed further based on the LD information. Several candidate genes may affect the regulation or development process which may be worth researching further (detail in Supplementary Table S10).

The GWAS results indicate that the Duroc population delivers fewer loci for fewer phenotypes. We conclude that the low yield of QTLs can be predominantly explained by the fixed QTLs for growth traits caused by artificial selection and the infinitesimal model for high heritability but the lack of major QTL traits. This result shows that the breeding of this commercial population has been successful, especially in terms of the improvement of growth traits. The next stage should focus on the use of genomic selection strategies for "infinitesimal traits" with high heritability but no major QTL, such as feeding behavior traits. We note that the feeding behavior traits had high or moderate heritability (Table 1) but a flat SNP distribution compared with TTN (Fig. 6), which [54] suggests that these traits may rely on a highly polygenic and complex genetic architecture. According to the GO and KEGG enrichment analyses, mostly neural activity process related functions or pathways were found to be enriched, especially the neurotrophin signaling pathway (P = 0.015) (Fig. S9). For example, NT3 and TrkB were reported to be involved in the regulation of the nervous system, affecting the stimulation of appetite [54, 55]. In all, we compared the inheritance models of 21 traits, and the results showed the difference between traits mainly affected by a limited number of loci and those affected by multiple loci with a small, widely distributed effect. Moreover, human selection could be a determining factor for the inheritance models of some specific traits in a specific population, making their genetic mechanism more complex. For further application of genomic selection, based on the QTL effect and the

inheritance model, a suitable prediction model should be designed for breeding to improve and optimize the accuracy of genomic prediction in animal breeding.

Conclusions

In conclusion, we discovered a Tn5-based, highly accurate, cost- and time-efficient LCS method to obtain whole genome SNP markers in a large Duroc population. We expect that our method could be applied to large-scale genome studies for any species without a good reference panel. GWAS results for 21 important agricultural traits identified tens of important QTLs/genes and showed their various genetic architectures, providing promising guidance for further genetic improvement harnessing genomic feature.

Methods

Animals, phenotyping, and DNA Extraction

The Duroc boars used for this study were born from September 2011 to September 2013. All boars were managed on a single nucleus farm in a commercial company, which enduring strong artificial selection for many years. The associated phenotype data used in this study included back fat thickness at 100 kg (BF), loin muscle area at 100 kg (LMA), loin muscle depth at 100 kg (LMD), lean meat percentage at 100 kg (LMP), average daily gain (0-30 kg and 30-100 kg) (ADG30 and ADG100), age to 30 kg and 100 kg daily weight (AGE30 and AGE100), body length (BL), body height (BH), circumference of cannon bone (CC), feed conversion ratio (FCR), average daily feed intake (ADFI), number of visits to feeder per day (NVD), time spent to eat per day (TPD), time spent to eat per visit (TPV), feed intake per visit (FPV), feed intake rate (FR), left teat number (LTN), right teat number (RTN), and total teat number (TTN). The phenotype TTN data were acquired from Tan's study [27]. In detail, the number of left and right teats of each pig were recorded within 48 h after birth, and only normal teats were counted. The total teat number in this study was the sum of normal left and right teats. Body weights were recorded at birth and at the beginning (30 ± 5 Kg) and

the end (100 ± 5 Kg) of the experiment. The ADG was calculated as the total weight gain over this time, divided by the number of days. The ages at which the pig reached 30 Kg and 100 Kg were recorded as AGE30 and AGE100 respectively. BF, LMD, LMA, and LMP were measured over the last three to four ribs using b-ultrasound-scan equipment when the weight of pigs reached 100 ± 5 Kg (Aloka SSD-500). Feeding behaviors including the time taken, duration, feed consumption, and weight of each pig were recorded at every visit by the Osborne FIRE Pig Performance Testing System (Kansas, American). The ADFI of each animal was obtained by dividing the total feed intake during the test by the number of days of the test period. The following feeding behavior and eating efficiency traits were defined and calculated for each boar: ADFI (Kg/day), TPD (min), NVD, TPV (= TPD/NVD, %), FPV (Kg), FR (= DFI/TPD, g/min), and FCR (=ADFI/ADG). The phenotypic values nearly all followed a normal distribution (Fig. S3).

Genomic DNA was extracted from the ear tissue using a DNeasy Blood & Tissue Kit (Qiagen 69506), assessed using a NanoDrop, and checked in 1% agarose gel. All samples were quantified using a Qubit 2.0 Fluorometer and then diluted to 40 ng/ml in 96-well plates.

Tn5 Library generation and sequencing

Equal amounts of Tn5ME-A/Tn5MErev and Tn5ME-B/Tn5MErev were incubated at 72 ℃ for 2 minutes and then placed on ice immediately. Tn5 (Karolinska Institute, Sweden) was loaded with Tn5ME-A+rev and Tn5ME-B+rev in 2× Tn5 dialysis buffer at 25 ℃ for 2 h. All linker oligonucleotides were the same as in a previous report [56].

Tagmentation were carried out at 55 ℃ for 10 minutes by mixing 4 μl 5×TAPS-$MgCl_2$, 2 μl of dimethylformamide (DMF) (Sigma Aldrich), 1 μl of the Tn5 pre-diluted to 16.5 ng/μl, 50 ng of DNA, and nuclease-free water. The total volume of the reaction was 20 μl. Then, 3.5 μl of 0.2% SDS was added, and Tn5 was inactivated for another 10 min at 55 ℃.

KAPA HiFi HotStart ReadyMix (Roche) was used for PCR amplification. The primers were designed for MGI sequencers, with the reverse primers containing 96

different index adaptors to distinguish individual libraries. The PCR program was as follows: 9 min at 72 ℃, 30 sec at 98 ℃, and then 9 cycles of 30 sec at 98 ℃, 30 sec at 63 ℃, followed by 3 min at 72 ℃. The products were quantified by Qubit Fluorometric Quantitation (Invitrogen) Then, the groups of 96 indexed samples were pooled with equal amounts.

Size selection was performed using AMPure XP beads (Beckmann), with a left side size selection ratio of 0.55× and a right side size selection ratio of 0.1×. The final libraries were sequenced on 2 lanes of MGISEQ-2000 to generate 2×100 bp paired-end reads or on 1 lane of BGISEQ-500 to generate 2×100 bp paired-end reads.

Genotype data obtained using high depth sequencing and SNP chip

We sequenced 37 out of the total 2,869 pigs using the Hiseq X Ten system at a high depth of 15.15×. GTX by the Genetalks company, a commercially available FPGA-based hardware accelerator platform, was used in this study for both mapping clean reads to the Sscrofa11.1 reference genome (ftp://ftp.ensembl.org/pub/release-99/fasta/sus_scrofa/dna/) and variant calling. The alignment process was accelerated by FPGA implementation of a parallel seed-and-extend approach based on the Smith–Waterman algorithm, while the variant calling process was accelerated by FPGA implementation of GATK HaplotypeCaller (PairHMM) [57]. GATK multi-sample best practice was used to call and genotype SNPs for the 37 pigs, and the SNPs were hard filtered with a relatively strict option “QD < 10.0 || ReadPosRankSum < -8.0 || FS > 10.0 || MQ<40.0”. The average running time from a fastq file to a bam file was about 3 min for each sample in this study.

We also selected 42 individuals who were included in the LCS dataset and genotyped using the GeneSeek Genomic Profiler Porcine 80K SNP Array and obtained 68,528 SNPs across the whole genome. The genotypes of the sex chromosomes were excluded from this study, and after quality control (genotype call rate > 0.95), 47,946 SNPs remained. We retained 45,308 SNPs that overlapped with the LCS dataset to evaluate the genotypes from the LCS strategy.

Low coverage sequencing data analyses

Sequencing reads from the low coverage samples were mapped to the Sscrofa11.1 reference genome using GTX-align, which includes a step that involves marking PCR duplicates. The indel realignment and base quality recalibration modules in GATK were applied to realign the reads around indel candidate loci and to recalibrate the base quality. Variant calling was done using the BaseVar and hard filtered with EAF >= 0.01 and a depth greater than or equal to 1.5 times the interquartile range. The detailed BaseVar algorithm that was used to call SNP variants and estimate allele frequency was described in a previous report [29]. We used STITCH [15] to impute genotype probabilities for all individuals. The key parameter K (number of ancestral haplotypes) was decided based on the tests in SSC18. Results were filtered with an imputation info score > 0.4 and a Hardy Weinberg Equilibrium (HWE) *P* value > $1e^{-6}$. After quality control, 2,797 individuals with genotype data were obtained. Two validation actions were taken to calculate the accuracy of imputation. The first parameter was genotypic concordance (GC), which was calculated as the number of correctly-imputed genotypes divided by the total number of sites. Another parameter was the allele dosage R^2, which was described in a previous report [30]. The SNPEff program [58] was used to annotate the variants.

Population genetics analysis

A subset of 258,662 SNPs that tagged all other SNPs with MAF > 1% at LD $r^2 < 0.98$ and a call rate of >95% were retained for downstream analysis. PCA clustering analyses were performed with GCTA software [59]. The average heterozygosity rate and MAF were obtained using the vcftools program [60]. Tajima's D [61] and diversity Pi was implemented to analyze selective sweep regions simultaneously with the window size set to 1 Mb, and only windows with an interquartile range for Tajima'D and diversity Pi of 1.5 fold in the whole genome were regarded as putative selection regions. Gene Ontology (GO) and Kyoto Encyclopedia of Genes and Genomes (KEGG) pathway enrichment analyses were performed using the OmicShare tools (http://www.omicshare.com/tools).

Genome-wide association and Heritability estimation

A mixed linear model (MLM) approach was used for the genome-wide association analyses, as implemented in the GCTA package [59]. The statistical model included the year and month as discrete covariates. For BF, LMA, LMD, and LMP, the year and season were included as discrete covariates, and the weights at the beginning and end of the test were used as quantitative covariates. To correct for multiple testing across the genome, the FDR correction obtained using FDRtool R package [62] was applied to determine the genome-wide significance threshold (FDR < 0.05). The SNP effect was estimated using the GREML_CE program in the GVCBLUP package [63], where the result was absoluted and normalized.

Heritability was estimated using a mixed model as follows:

$$\mathbf{y} = \mathbf{X_b b} + \mathbf{Za} + \mathbf{e}$$

with Var($\mathbf{y}$) = $\mathbf{ZA_a Z'}\sigma_a^2 + \mathbf{I}\sigma_e^2$, where Z is an incidence matrix allocating phenotypic observations to each animal; **b** is the vector of the fixed year-month effects for BF, LMA, LMD, and LMP that also includes the weights at the beginning and end of the test as covariance; $\mathbf{X_b}$ is the incidence matrix for **b**; **a** is the vector of additive values based on the genotype data; $\mathbf{A}_a$ is a genomic additive relationship matrix; σ_a^2 is the additive variance; and σ_e^2 is the residual variance. Variance components were estimated by genomic restricted maximum likelihood estimation (GREML) using the GREML_CE program in the GVCBLUP package. The additive heritability was defined as: $h_a^2 = \sigma_a^2 / (\sigma_a^2 + \sigma_e^2)$. SNP effects were defined by the GREML_CE program and then normalized using R script.

The heritability of the detected QTL was estimated as follows:

$$\mathbf{y} = \mathbf{X'_b b'} + \mathbf{Za} + \mathbf{e}$$

with Var($\mathbf{y}$) = $\mathbf{ZA_a Z'}\sigma_a^2 + \mathbf{I}\sigma_e^2$, where Z is an incidence matrix allocating phenotypic observations to each animal; **b’** is the vector of the fixed year-month effects and significant SNPs identified in the QTL region using GWAS analysis for BF, LMA, LMD and LMP; **b** also includes the weights at the beginning and end of the test as covariance; $\mathbf{X'_b}$ is the incidence matrix for **b**; **a** is the vector of additive values based on

the genotype data; $\mathbf{A}_a$ is a genomic additive relationship matrix; σ_a^2 is the additive variance; and σ_e^2 is the residual variance. The QTL heritability was defined as $h_{qtl}^2 = h_a^2 - \sigma_a^2 /(\sigma_a^2 + \sigma_e^2)$.

Functional Consequence of the Missense Mutations associated with TN

The effect of the missense SNPs associated with TN on the stability of pig ABCD4, PROX2, and DLST proteins was assessed using I-Mutant adaptation 2.0 [64]. A potential surge or reduction in the DDG was predicted, along with a reliability index (RI), where the lowest and highest reliability levels were 0 and 10, respectively.

Data availability

All of the sequencing raw data in this study have been deposited into NCBI with accession number PRJNA681437, and the variance data as VCF file will be available via GIGADB.

Abbreviations:

LCS: Low coverage sequencing method; GC: genotypic concordance; TTN: Total teat number; LTN: Left teat number; RTN: Right teat number; BF: Back fat thickness at 100 Kg; LMD: Loin muscle depth at 100 Kg; LMA: Loin muscle area at 100 Kg; LMP: Lean meat percentage at 100 Kg; TPD: Time spent to eat per day; ADFI: Average daily feed intake; NVD: Number of visits to feeder per day; TPV: Time spent to eat per visit; FR: Feed intake rate; FPV: Feed intake per visit; FCR: Feed conversion rate; ADG30: Average daily gain (0-30 Kg); AGE30: Age to 30 kg live weight; ADG100: Average daily gain (30-100 Kg); AGE100: Age to 100 kg live weight; BL: Body length; BH: Body height; CC: Circumference of cannon bone.

Competing interests

The authors declare that they have no competing interests.

Funding

This study is supported by the financial support of the National Transgenic Grand Project [2016ZX08009003-006], the 948 Program of the Ministry of Agriculture of China (2012-G1(4)), the Science and Technology Innovation Strategy Projects of Guangdong Province [2019B020203002], and the Guangdong Academician Workstation [2011A090700016].

Authors' contributions

XH, YW, and ZW designed the research. YW and RY led the writing of the paper. RY, DZ and YW analyzed the data and generated the models. XG, JR, ZH, CB and CT contributed sequencing method, reagents, materials, and phenotype. RY, YW, XH, YZ, GC and DL contributed to the interpretation of the results and edited the paper.

Acknowledgements

The authors thank Zhuo Song, Chungen Yi, and Wenjuan Wei for providing the services of FPGA-based hardware accelerator platform and the Batch Compute system in Aliyun cloud. The authors also thank Siyang Liu and Xun Xu for their valuable suggestions on data analyses, and Li Ma and Zhaoliang Liu for improving the manuscript. Part of the analysis was performed on the high-performance computing platform of the State Key Laboratory of Agrobiotechnology.

References

1. Visscher PM, Brown MA, McCarthy MI and Yang J. Five years of GWAS discovery. Am J Hum Genet. 2012;90 1:7-24. doi:10.1016/j.ajhg.2011.11.029.
2. Huang X, Wei X, Sang T, Zhao Q, Feng Q, Zhao Y, et al. Genome-wide association studies of 14 agronomic traits in rice landraces. Nat Genet. 2010;42 11:961-7. doi:10.1038/ng.695.
3. Marchini J and Howie B. Genotype imputation for genome-wide association studies. Nat Rev Genet. 2010;11 7:499-511. doi:10.1038/nrg2796.
4. Marchini J, Howie B, Myers S, McVean G and Donnelly P. A new multipoint

method for genome-wide association studies by imputation of genotypes. Nat Genet. 2007;39 7:906-13. doi:10.1038/ng2088.

5. Howie BN, Donnelly P and Marchini J. A flexible and accurate genotype imputation method for the next generation of genome-wide association studies. PLoS Genet. 2009;5 6:e1000529. doi:10.1371/journal.pgen.1000529.

6. Howie B, Fuchsberger C, Stephens M, Marchini J and Abecasis GR. Fast and accurate genotype imputation in genome-wide association studies through pre-phasing. Nature Genetics. 2012;44 8:955-+. doi:10.1038/ng.2354.

7. Yan G, Qiao R, Zhang F, Xin W, Xiao S, Huang T, et al. Imputation-Based Whole-Genome Sequence Association Study Rediscovered the Missing QTL for Lumbar Number in Sutai Pigs. Sci Rep. 2017;7 1:615. doi:10.1038/s41598-017-00729-0.

8. van Binsbergen R, Bink MC, Calus MP, van Eeuwijk FA, Hayes BJ, Hulsegge I, et al. Accuracy of imputation to whole-genome sequence data in Holstein Friesian cattle. Genet Sel Evol. 2014;46:41. doi:10.1186/1297-9686-46-41.

9. van den Berg S, Vandenplas J, van Eeuwijk FA, Bouwman AC, Lopes MS and Veerkamp RF. Imputation to whole-genome sequence using multiple pig populations and its use in genome-wide association studies. Genetics Selection Evolution. 2019;51 doi:10.1186/s12711-019-0445-y.

10. Swarts K, Li HH, Navarro JAR, An D, Romay MC, Hearne S, et al. Novel Methods to Optimize Genotypic Imputation for Low-Coverage, Next-Generation Sequence Data in Crop Plants. Plant Genome-Us. 2014;7 3 doi:10.3835/plantgenome2014.05.0023.

11. Buerkle CA and Gompert Z. Population genomics based on low coverage sequencing: how low should we go? Mol Ecol. 2013;22 11:3028-35. doi:10.1111/mec.12105.

12. Huang L, Wang B, Chen RT, Bercovici S and Batzoglou S. Reveel: large-scale population genotyping using low-coverage sequencing data. Bioinformatics. 2016;32 11:1686-96. doi:10.1093/bioinformatics/btv530.

13. Li Y, Sidore C, Kang HM, Boehnke M and Abecasis GR. Low-coverage sequencing: implications for design of complex trait association studies. Genome Res. 2011;21 6:940-51. doi:10.1101/gr.117259.110.

14. Gilly A, Southam L, Suveges D, Kuchenbaecker K, Moore R, Melloni GEM, et al. Very low-depth whole-genome sequencing in complex trait association studies. Bioinformatics. 2019;35 15:2555-61. doi:10.1093/bioinformatics/bty1032.

15. Davies RW, Flint J, Myers S and Mott R. Rapid genotype imputation from sequence without reference panels. Nature Genetics. 2016;48 8:965-+. doi:10.1038/ng.3594.

16. Ros-Freixedes R, Gonen S, Gorjanc G and Hickey JM. A method for allocating low-coverage sequencing resources by targeting haplotypes rather than individuals. Genetics Selection Evolution. 2017;49 doi:ARTN 78 10.1186/s12711-017-0353-y.

17. Fragoso CA, Heffelfinger C, Zhao HY and Dellaporta SL. Imputing Genotypes in Biallelic Populations from Low-Coverage Sequence Data. Genetics. 2016;202 2:487-+. doi:10.1534/genetics.115.182071.

18. Bickhart DM, Hutchison JL, Null DJ, VanRaden PM and Cole JB. Reducing animal sequencing redundancy by preferentially selecting animals with low-frequency haplotypes. J Dairy Sci. 2016;99 7:5526-34. doi:10.3168/jds.2015-10347.

19. Zan Y, Payen T, Lillie M, Honaker CF, Siegel PB and Carlborg O. Genotyping by low-coverage whole-genome sequencing in intercross pedigrees from outbred founders: a cost-efficient approach. Genet Sel Evol. 2019;51 1:44. doi:10.1186/s12711-019-0487-1.

20. Nicod J, Davies RW, Cai N, Hassett C, Goodstadt L, Cosgrove C, et al. Genome-wide association of multiple complex traits in outbred mice by ultra-low-coverage sequencing. Nat Genet. 2016;48 8:912-8. doi:10.1038/ng.3595.

21. Genomes Project C, Abecasis GR, Altshuler D, Auton A, Brooks LD, Durbin

RM, et al. A map of human genome variation from population-scale sequencing. Nature. 2010;467 7319:1061-73. doi:10.1038/nature09534.

22. Lam HM, Xu X, Liu X, Chen W, Yang G, Wong FL, et al. Resequencing of 31 wild and cultivated soybean genomes identifies patterns of genetic diversity and selection. Nat Genet. 2010;42 12:1053-9. doi:10.1038/ng.715.

23. Daetwyler HD, Capitan A, Pausch H, Stothard P, van Binsbergen R, Brondum RF, et al. Whole-genome sequencing of 234 bulls facilitates mapping of monogenic and complex traits in cattle. Nat Genet. 2014;46 8:858-65. doi:10.1038/ng.3034.

24. Hayes BJ and Daetwyler HD. 1000 Bull Genomes Project to Map Simple and Complex Genetic Traits in Cattle: Applications and Outcomes. Annu Rev Anim Biosci. 2019;7:89-102. doi:10.1146/annurev-animal-020518-115024.

25. Yang J, Benyamin B, McEvoy BP, Gordon S, Henders AK, Nyholt DR, et al. Common SNPs explain a large proportion of the heritability for human height. Nat Genet. 2010;42 7:565-9. doi:10.1038/ng.608.

26. Lango Allen H, Estrada K, Lettre G, Berndt SI, Weedon MN, Rivadeneira F, et al. Hundreds of variants clustered in genomic loci and biological pathways affect human height. Nature. 2010;467 7317:832-8. doi:10.1038/nature09410.

27. Tan C, Wu ZF, Ren JL, Huang ZL, Liu DW, He XY, et al. Genome-wide association study and accuracy of genomic prediction for teat number in Duroc pigs using genotyping-by-sequencing. Genetics Selection Evolution. 2017;49 doi:ARTN 35 10.1186/s12711-017-0311-8.

28. Ros-Freixedes R, Battagin M, Johnsson M, Gorjanc G, Mileham AJ, Rounsley SD, et al. Impact of index hopping and bias towards the reference allele on accuracy of genotype calls from low-coverage sequencing. Genet Sel Evol. 2018;50 1:64. doi:10.1186/s12711-018-0436-4.

29. Liu S, Huang S, Chen F, Zhao L, Yuan Y, Francis SS, et al. Genomic Analyses from Non-invasive Prenatal Testing Reveal Genetic Associations, Patterns of Viral Infections, and Chinese Population History. Cell. 2018;175 2:347-59 e14.

doi:10.1016/j.cell.2018.08.016.

30. Browning BL and Browning SR. A unified approach to genotype imputation and haplotype-phase inference for large data sets of trios and unrelated individuals. Am J Hum Genet. 2009;84 2:210-23. doi:10.1016/j.ajhg.2009.01.005.

31. Pistocchi A, Bartesaghi S, Cotelli F and Del Giacco L. Identification and expression pattern of zebrafish prox2 during embryonic development. Dev Dyn. 2008;237 12:3916-20. doi:10.1002/dvdy.21798.

32. Gong H, Xiao S, Li W, Huang T, Huang X, Yan G, et al. Unravelling the genetic loci for growth and carcass traits in Chinese Bamaxiang pigs based on a 1.4 million SNP array. J Anim Breed Genet. 2019;136 1:3-14. doi:10.1111/jbg.12365.

33. Liu X, Wang LG, Liang J, Yan H, Zhao KB, Li N, et al. Genome-Wide Association Study for Certain Carcass Traits and Organ Weights in a Large WhitexMinzhu Intercross Porcine Population. J Integr Agr. 2014;13 12:2721-30. doi:10.1016/S2095-3119(14)60787-5.

34. Arce-Cerezo A, Garcia M, Rodriguez-Nuevo A, Crosa-Bonell M, Enguix N, Pero A, et al. HMGA1 overexpression in adipose tissue impairs adipogenesis and prevents diet-induced obesity and insulin resistance. Sci Rep. 2015;5:14487. doi:10.1038/srep14487.

35. Wang LG, Zhang LC, Yan H, Liu X, Li N, Liang J, et al. Genome-Wide Association Studies Identify the Loci for 5 Exterior Traits in a Large White x Minzhu Pig Population. Plos One. 2014;9 8 doi:ARTN e103766 10.1371/journal.pone.0103766.

36. Ji JX, Yan GR, Chen D, Xiao SJ, Gao J and Zhang ZY. An association study using imputed whole-genome sequence data identifies novel significant loci for growth-related traits in a Duroc x Erhualian F-2 population. Journal of Animal Breeding and Genetics. 2019;136 3:217-28. doi:10.1111/jbg.12389.

37. Meuwissen T and Goddard M. Accurate prediction of genetic values for

complex traits by whole-genome resequencing. Genetics. 2010;185 2:623-31. doi:10.1534/genetics.110.116590.

38. Zhang C, Kemp RA, Stothard P, Wang Z, Boddicker N, Krivushin K, et al. Genomic evaluation of feed efficiency component traits in Duroc pigs using 80K, 650K and whole-genome sequence variants. Genet Sel Evol. 2018;50 1:14. doi:10.1186/s12711-018-0387-9.

39. Yan G, Guo T, Xiao S, Zhang F, Xin W, Huang T, et al. Imputation-Based Whole-Genome Sequence Association Study Reveals Constant and Novel Loci for Hematological Traits in a Large-Scale Swine F2 Resource Population. Front Genet. 2018;9:401. doi:10.3389/fgene.2018.00401.

40. Meuwissen TH, Hayes BJ and Goddard ME. Prediction of total genetic value using genome-wide dense marker maps. Genetics. 2001;157 4:1819-29.

41. Zhang Z, Ding X, Liu J, Koning DJD and Zhang Q. Genomic selection for QTL-MAS data using a trait-specific relationship matrix. Bmc Proceedings. 2011;5 Suppl 3 Suppl 3:S15.

42. Xing Y, Li G, Wang Z, Feng B, Song Z and Wu C. GTZ: a fast compression and cloud transmission tool optimized for FASTQ files. BMC Bioinformatics. 2017;18 Suppl 16:549. doi:10.1186/s12859-017-1973-5.

43. Groenen MA, Archibald AL, Uenishi H, Tuggle CK, Takeuchi Y, Rothschild MF, et al. Analyses of pig genomes provide insight into porcine demography and evolution. Nature. 2012;491 7424:393-8. doi:10.1038/nature11622.

44. Rubin CJ, Megens HJ, Martinez Barrio A, Maqbool K, Sayyab S, Schwochow D, et al. Strong signatures of selection in the domestic pig genome. Proc Natl Acad Sci U S A. 2012;109 48:19529-36. doi:10.1073/pnas.1217149109.

45. Bosse M, Megens HJ, Frantz LA, Madsen O, Larson G, Paudel Y, et al. Genomic analysis reveals selection for Asian genes in European pigs following human-mediated introgression. Nat Commun. 2014;5:4392. doi:10.1038/ncomms5392.

46. Bosse M, Lopes MS, Madsen O, Megens HJ, Crooijmans RP, Frantz LA, et al. Artificial selection on introduced Asian haplotypes shaped the genetic

architecture in European commercial pigs. Proc Biol Sci. 2015;282 1821:20152019. doi:10.1098/rspb.2015.2019.

47. Ma Y, Zhang S, Zhang K, Fang C, Xie S, Du X, et al. Genomic Analysis To Identify Signatures of Artificial Selection and Loci Associated with Important Economic Traits in Duroc Pigs. G3 (Bethesda). 2018;8 11:3617-25. doi:10.1534/g3.118.200665.

48. Stratil A, Van Poucke M, Bartenschlager H, Knoll A, Yerle M, Peelman LJ, et al. Porcine OGN and ASPN: mapping, polymorphisms and use for quantitative trait loci identification for growth and carcass traits in a Meishan x Pietrain intercross. Anim Genet. 2006;37 4:415-8. doi:10.1111/j.1365-2052.2006.01480.x.

49. Sun W, Hu S, Hu J, Qiu J, Yang S, Hu B, et al. Akirin1 promotes myoblast differentiation by modulating multiple myoblast differentiation factors. Biosci Rep. 2019;39 3 doi:10.1042/BSR20182152.

50. Song Y, Altarejos J, Goodarzi MO, Inoue H, Guo X, Berdeaux R, et al. CRTC3 links catecholamine signalling to energy balance. Nature. 2010;468 7326:933-9. doi:10.1038/nature09564.

51. Liu J, Nong Q, Wang J, Chen W, Xu Z, You W, et al. Breed difference and regulatory role of CRTC3 in porcine intramuscular adipocyte. Anim Genet. 2020; doi:10.1111/age.12945.

52. Hoover KC. Smell with inspiration: the evolutionary significance of olfaction. Am J Phys Anthropol. 2010;143 Suppl 51:63-74. doi:10.1002/ajpa.21441.

53. Carneiro M, Rubin CJ, Di Palma F, Albert FW, Alfoldi J, Martinez Barrio A, et al. Rabbit genome analysis reveals a polygenic basis for phenotypic change during domestication. Science. 2014;345 6200:1074-9. doi:10.1126/science.1253714.

54. Tsao D, Thomsen HK, Chou J, Stratton J, Hagen M, Loo C, et al. TrkB agonists ameliorate obesity and associated metabolic conditions in mice. Endocrinology. 2008;149 3:1038-48. doi:10.1210/en.2007-1166.

55. Lebrun B, Bariohay B, Moyse E and Jean A. Brain-derived neurotrophic factor (BDNF) and food intake regulation: a minireview. Auton Neurosci. 2006;126-127:30-8. doi:10.1016/j.autneu.2006.02.027.

56. Picelli S, Bjorklund AK, Reinius B, Sagasser S, Winberg G and Sandberg R. Tn5 transposase and tagmentation procedures for massively scaled sequencing projects. Genome Res. 2014;24 12:2033-40. doi:10.1101/gr.177881.114.

57. McKenna A, Hanna M, Banks E, Sivachenko A, Cibulskis K, Kernytsky A, et al. The Genome Analysis Toolkit: a MapReduce framework for analyzing next-generation DNA sequencing data. Genome Res. 2010;20 9:1297-303. doi:10.1101/gr.107524.110.

58. Cingolani P, Platts A, Wang le L, Coon M, Nguyen T, Wang L, et al. A program for annotating and predicting the effects of single nucleotide polymorphisms, SnpEff: SNPs in the genome of Drosophila melanogaster strain w1118; iso-2; iso-3. Fly (Austin). 2012;6 2:80-92. doi:10.4161/fly.19695.

59. Yang J, Lee SH, Goddard ME and Visscher PM. GCTA: a tool for genome-wide complex trait analysis. Am J Hum Genet. 2011;88 1:76-82. doi:10.1016/j.ajhg.2010.11.011.

60. Danecek P, Auton A, Abecasis G, Albers CA, Banks E, DePristo MA, et al. The variant call format and VCFtools. Bioinformatics. 2011;27 15:2156-8. doi:10.1093/bioinformatics/btr330.

61. Tajima F. Statistical method for testing the neutral mutation hypothesis by DNA polymorphism. Genetics. 1989;123 3:585-95.

62. Strimmer K. fdrtool: a versatile R package for estimating local and tail area-based false discovery rates. Bioinformatics. 2008;24 12:1461-2. doi:10.1093/bioinformatics/btn209.

63. Wang C, Prakapenka D, Wang S, Pulugurta S, Runesha HB and Da Y. GVCBLUP: a computer package for genomic prediction and variance component estimation of additive and dominance effects. BMC Bioinformatics. 2014;15:270. doi:10.1186/1471-2105-15-270.

64. Capriotti E, Calabrese R and Casadio R. Predicting the insurgence of human genetic diseases associated to single point protein mutations with support vector machines and evolutionary information. Bioinformatics. 2006;22 22:2729-34. doi:10.1093/bioinformatics/btl423.

Figure legends

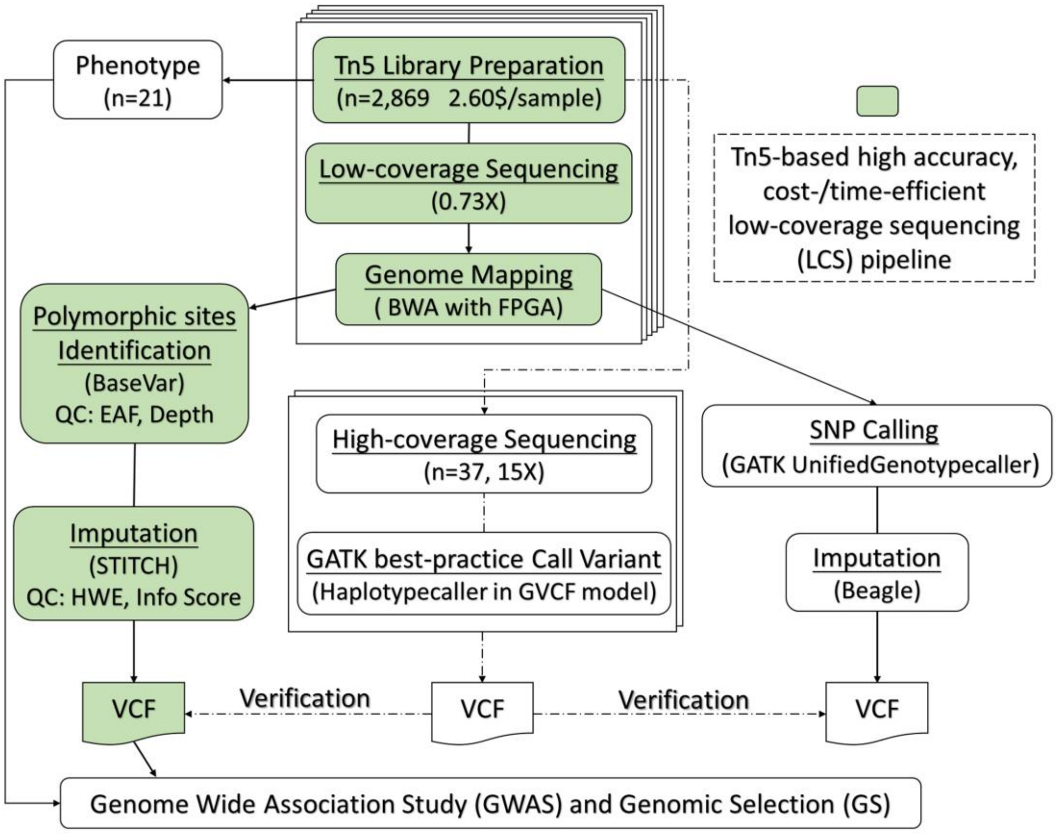

Figure 1 The low coverage sequencing (LCS) study design

The flow chart summarizes the steps used to identify and impute polymorphic sites, where the green block (left) represents the highly accurate pipeline used for the Tn5-based LCS analysis (BaseVar-STITCH). We also generated SNP results using the GATK-Beagle pipeline (right) and compared them with those found with the BaseVar-STITCH method. The data generated from the high-coverage sequencing analyses (middle) were used to assess the accuracy of the above results. The BaseVar-STITCH pipeline was used in the further GWAS presented in this study.

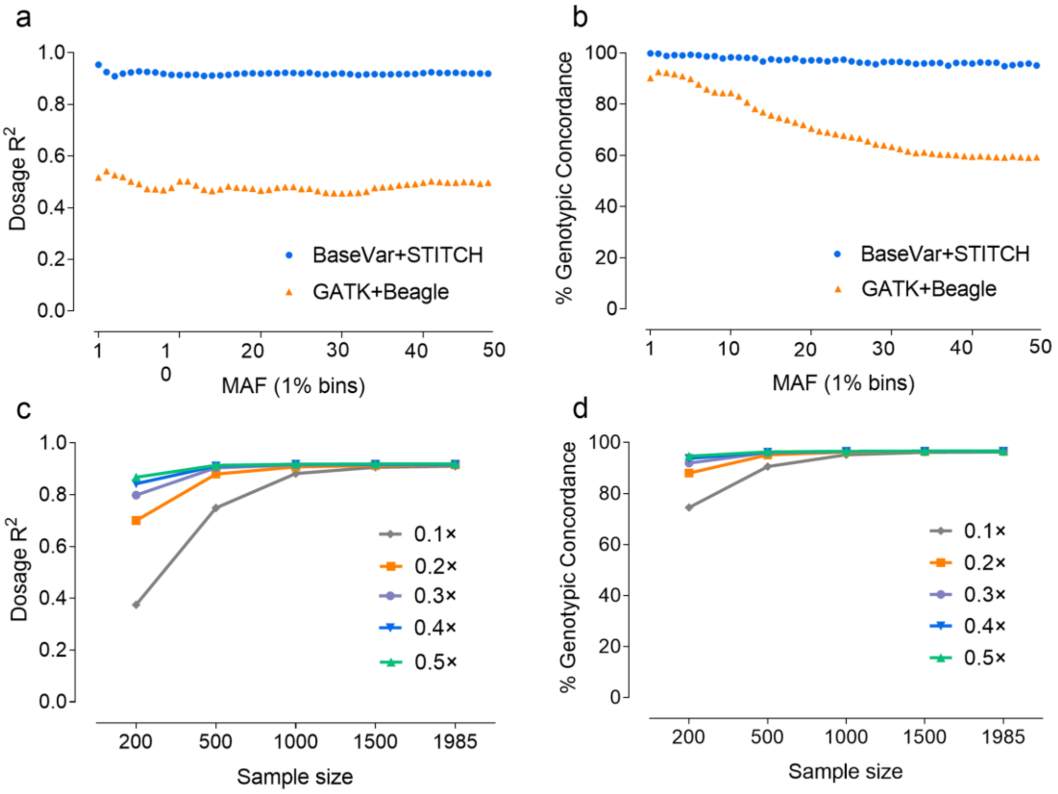

Figure 2 Performance of BaseVar-STITCH on different minor allele frequencies (MAFs) and sample sizes

The validation dataset is the high coverage sequencing results of 37 individuals genotyped by GATK best practices (HaplotypeCaller model). **(a)** and **(b)** show a comparison of the dosage R^2 and genotypic concordance values (%) between the BaseVar-STITCH for low-coverage sequencing (LCS) (blue) and the GATK-Beagle (orange) pipelines, and **(c)** and **(d)** show the comparison of the dosage R^2 and genotypic concordance values (%) among different sequencing depths.

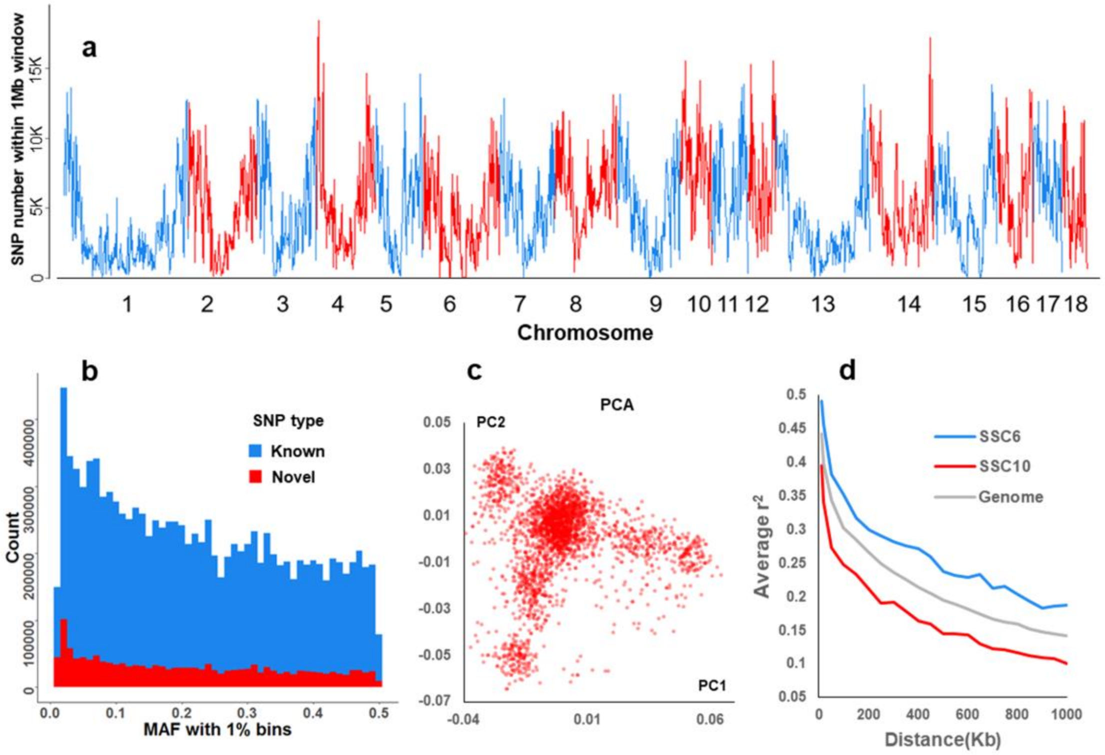

Figure 3 Genetic diversity of the Duroc population

(a) The distribution of SNPs in 1 Mb windows across the genome. **(b)** Histogram of allele counts by each 1% MAF bin. Novel (red) and known SNP sets (blue) were defined by comparing them to the pig dbSNP database. **(c)** Principal component 1 and 2 distribution in the Duroc population. **(d)** The extent of linkage disequilibrium (LD), in which the LD on chromosomes 6 (SSC6) and 10 (SSC10) represent the highest and lowest levels across the whole genome, respectively.

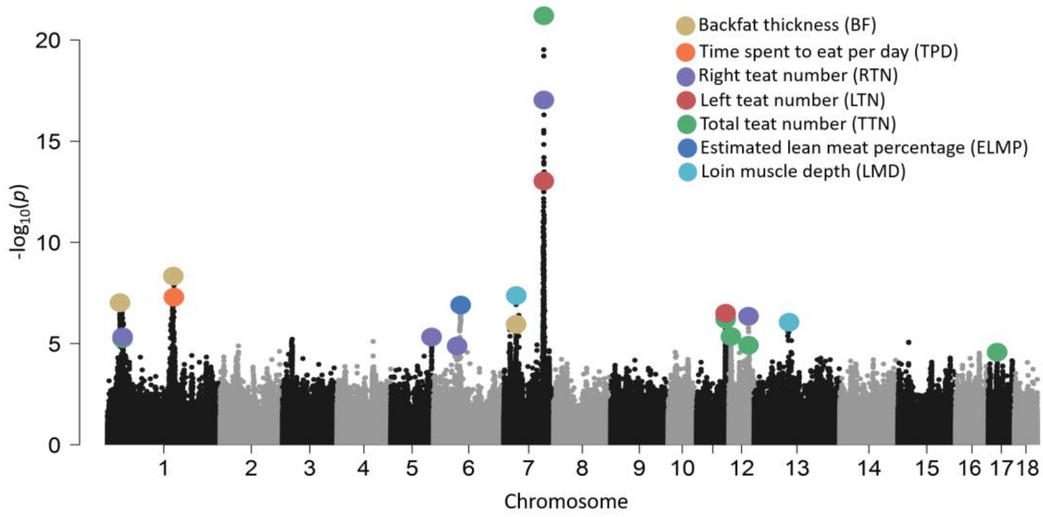

Figure 4 Summary Manhattan plot of seven phenotypes with significant SNPs

Genome-wide representation of all quantitative trait loci (QTLs) identified in this study. Light and dark grey dots show associations from the seven measures where at least one QTL was detected at the tagging SNP positions (n = 258,662). The most significant SNP positions at each QTL are marked with a color dot.

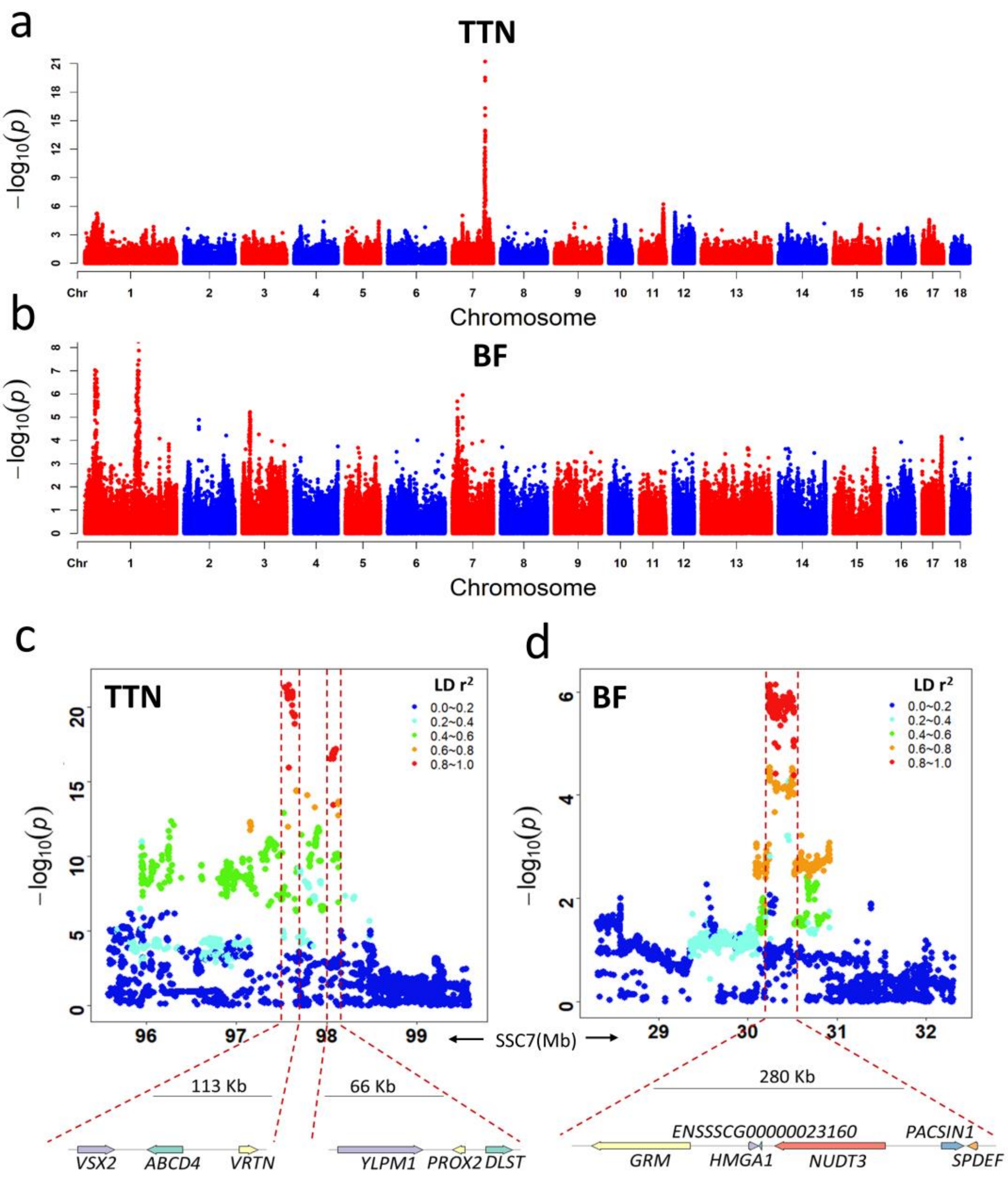

Figure 5 Manhattan plots and fine-mapping of the total teat number (TTN) and back fat thickness (BF)

(a) and (**b)** Depict the TTN and BF association signals on the whole genome. **(c)** Fine-mapping of the TTN using the entire set of SNPs, in which two isolated regions on chromosome 7 with lengths of 113 and 66 Kb were detected as QTLs. **(d)** Fine-mapping of BF using the entire set of SNPs. A narrow QTL with a length of 280 Kb was detected on chromosome 7. The association genes within QTLs are displayed below.

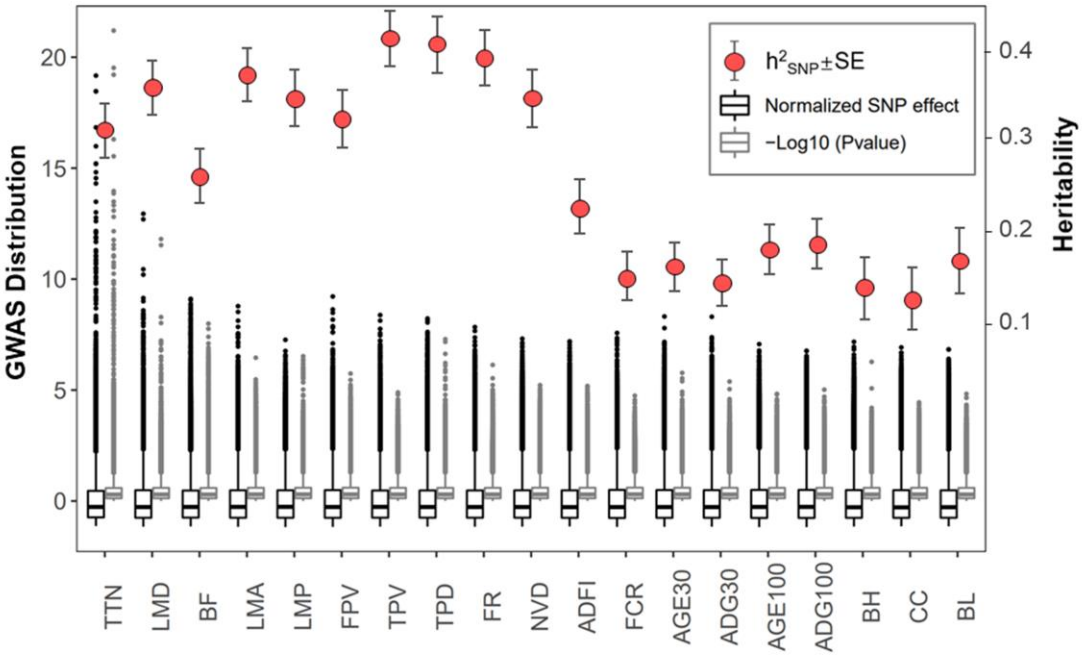

Figure 6 Heritability and SNP significance and normalized effect of 21 traits

The SNP effect was estimated and normalized and is displayed in the black boxplot. The gray boxplot represents the distribution of -$\log_{10} P$ values of all SNPs. Heritability estimates are represented by red dots, and black lines represent standard deviations.

Table 1. QTLs mapping and contribution to heritability

Phenotype	Number	Mean ± standard deviation	Significant threshold[a]	QTL number	Variance explained(%)[b]	Gene number[c]
Total teat number (TTN)	2797	10.73 ± 1.07	4.55	6	8.86	52
Left teat number (LTN)	2797	5.35 ± 0.66	4.81	2	3.16	14
Right teat number (RTN)	2797	5.38 ± 0.64	4.79	5	6.03	56
Back fat thickness at 100 Kg (BF, mm)	2796	10.99 ± 2.66	4.67	4	2.40	55
Loin muscle depth at 100 Kg (LMD, mm)	2796	46.15 ± 3.93	5.36	2	1.27	15
Loin muscle area at 100 Kg (LMA, mm^2)	2795	36.25 ± 3.60	-	0	0	0
Lean meat percentage at 100 Kg (LMP, %)	2795	54.02 ± 1.58	5.50	1	1.19	48
Time spent to eat per day (TPD, min)	2602	63.02 ± 9.85	6.10	1	1.08	28
Average daily feed intake (ADFI, Kg)	2602	2.00 ± 0.20	-	0	0	0
Number of visits to feeder per day (NVD)	2602	7.30 ± 1.83	-	0	0	0
Time spent to eat per visit (TPV, min)	2602	10.06 ± 2.79	-	0	0	0
Feed intake rate (FR, g/min)	2602	32.37 ± 5.19	-	0	0	0
Feed intake per visit (FPV, Kg)	2602	290.6 ± 75.87	-	0	0	0
Feed conversion rate (FCR)	2691	2.19 ± 0.19	-	0	0	0
Average daily gain (0-30 Kg) (ADG30, g)	2795	354.8 ± 38.72	-	0	0	0
Age to 30 kg live weight (AGE30, day)	2796	80.49 ± 8.57	-	0	0	0
Average daily gain (30-100 Kg) (ADG100, g)	2795	633.8 ± 37.12	-	0	0	0
Age to 100 kg live weight (AGE100, day)	2796	155.5 ± 9.20	-	0	0	0
Body length (BL, cm)	1844	117.60 ± 2.91	-	0	0	0
Body height (BH, cm)	1844	62.19 ± 1.55	-	0	0	0
Circumference of cannon bone (CC, cm)	1844	17.81 ± 0.54	-	0	0	0

a. $-\text{Log}_{10}(p)$ value when FDR < 0.05; b. total phenotypic variance explained by QTLs; c. Total gene number included in QTLs.

Additional Files

Supplementary Figure 1 Dosage R^2 and cost time (minute) among different K values

Accuracy and cost time of genotyping from K = 5 to K = 25, where the blue and black lines represent the dosage R^2 and cost time (minute) respectively.

Supplementary Figure 2 Purifying selection regions in the whole genome

Purifying selection signals were detected on SSC2, SSC3, SSC6, SSC7, SSC9 and SSC15, where blue and red lines represent –Log_{10} Pi and Tajima's D respectively, and the grey regions depict the purifying selected regions.

Supplementary Figure 3 Phenotypic distribution of 21 traits

Supplementary Figure 4 Manhattan plots of phenotypes with no significant SNPs

Manhattan plots of ADFT, NVD, TPV, FPV, FR, FCR, BH, BL, CC, ADG100, AGE100, ADG30, AGE30 and LMA, where no significant SNPs were detected in these traits.

Supplementary Figure 5 QQ plot of 21 phenotypes

Supplementary Figure 6 Summary plots of fine mapping

Supplementary Figure 7 Distribution of top 100 SNPs based on *P* value using GWAS analysis

Supplementary Figure 8 GO and KEGG enrichment of genes identified to be associated with feeding behavior traits

Supplementary Figure 9 Neurotrophin signaling pathway enrichment

The red tangles represent detected pathways in this study, which including Bcl-2, NT3, TrkB and p75NTF.

Supplementary Table S1 LC data set

Supplementary Table S2 Resequencing Duroc samples list

Supplementary Table S3 Number and density of SNPs imputed by STITCH and Tag SNP

Supplementary Table S4 GO enrichment of genes located in the selected regions

Supplementary Table S5 Summary of detected QTLs

Supplementary Table S6 Summary table of markers identified significantly associated with ADG, AGE or FCR in previous studies

Supplementary Table S7 GO enrichment of genes located in the selected regions

Supplementary Table S8 KEGG enrichment of genes located in the selected regions

Supplementary Table S9 Missense SNPs in the narrowed QTL region of TN

Supplementary Table S10 Gathered information of candidate genes

 Click here to access/download;Table;Table 1.docx

Table 1 QTLs mapping and contribution to heritability

Phenotype	Number	Mean ± standard deviation	Significant threshold[a]	QTL number	Variance explained(%)[b]	Gene number[c]
Total teat number (TTN)	2797	10.73 ± 1.07	4.55	6	8.86	52
Left teat number (LTN)	2797	5.35 ± 0.66	4.81	2	3.16	14
Right teat number (RTN)	2797	5.38 ± 0.64	4.79	5	6.03	56
Back fat thickness at 100 Kg (BF, mm)	2796	10.99 ± 2.66	4.67	4	2.40	55
Loin muscle depth at 100 Kg (LMD, mm)	2796	46.15 ± 3.93	5.36	2	1.27	15
Loin muscle area at 100 Kg (LMA, mm^2)	2795	36.25 ± 3.60	-	0	0	0
Lean meat percentage at 100 Kg (LMP, %)	2795	54.02 ± 1.58	5.50	1	1.19	48
Time spent to eat per day (TPD, min)	2602	63.02 ± 9.85	6.10	1	1.08	28
Average daily feed intake (ADFI, Kg)	2602	2.00 ± 0.20	-	0	0	0
Number of visits to feeder per day (NVD)	2602	7.30 ± 1.83	-	0	0	0
Time spent to eat per visit (TPV, min)	2602	10.06 ± 2.79	-	0	0	0
Feed intake rate (FR, g/min)	2602	32.37 ± 5.19	-	0	0	0
Feed intake per visit (FPV, Kg)	2602	290.6 ± 75.87	-	0	0	0
Feed conversion rate (FCR)	2691	2.19 ± 0.19	-	0	0	0
Average daily gain (0-30 Kg) (ADG30, g)	2795	354.8 ± 38.72	-	0	0	0
Age to 30 kg live weight (AGE30, day)	2796	80.49 ± 8.57	-	0	0	0
Average daily gain (30-100 Kg) (ADG100, g)	2795	633.8 ± 37.12	-	0	0	0
Age to 100 kg live weight (AGE100, day)	2796	155.5 ± 9.20	-	0	0	0
Body length (BL, cm)	1844	117.60 ± 2.91	-	0	0	0
Body height (BH, cm)	1844	62.19 ± 1.55	-	0	0	0
Circumference of cannon bone (CC, cm)	1844	17.81 ± 0.54	-	0	0	0

Note: a. $-\text{Log}_{10}$ *P* value when FDR < 0.05; b. total phenotypic variance explained by QTLs; c. Total gene number included in QTLs.

Figure 1

Click here to access/download;Figure;Figure 1.tif

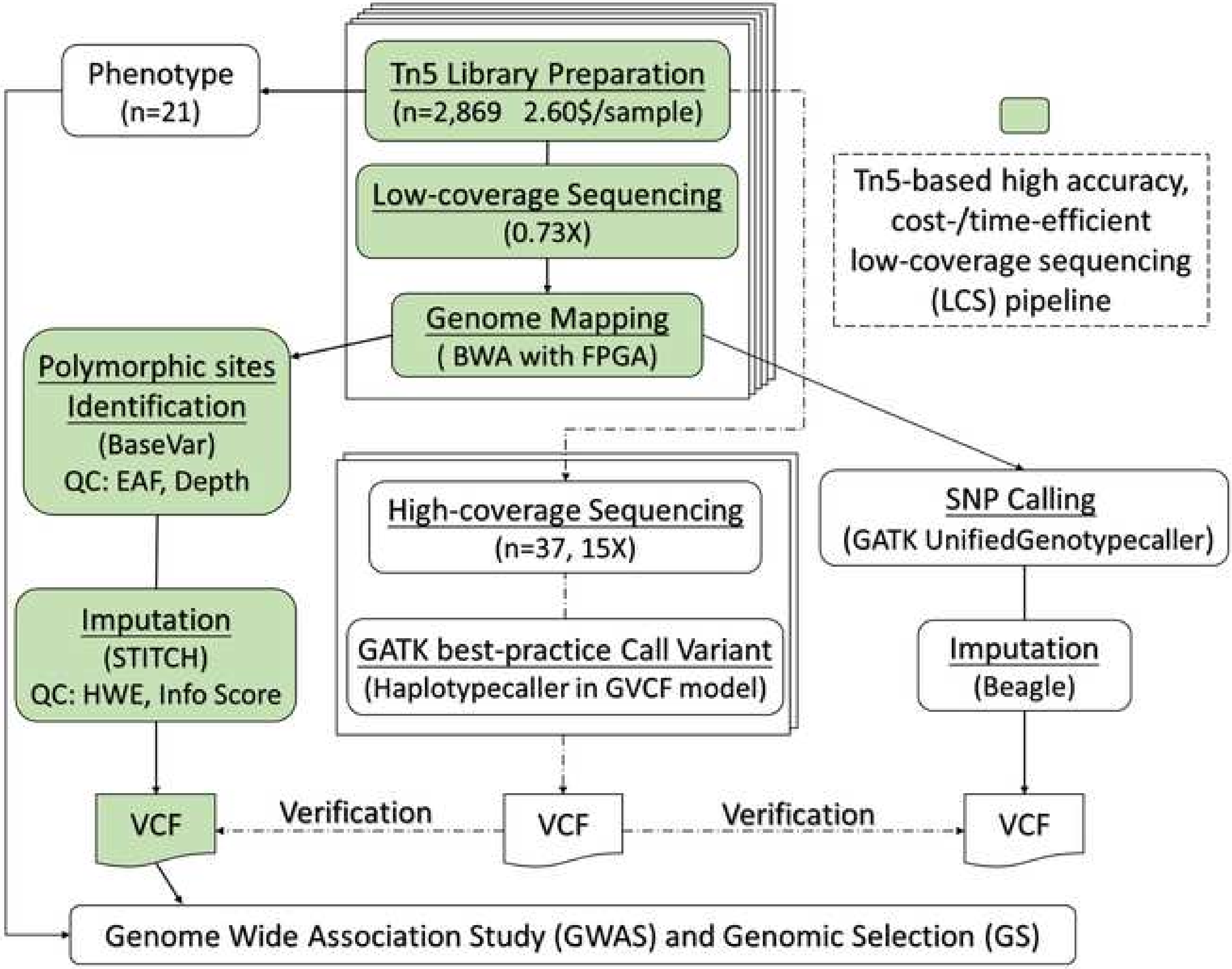

Figure 2

Click here to access/download;Figure;Figure 2.tif

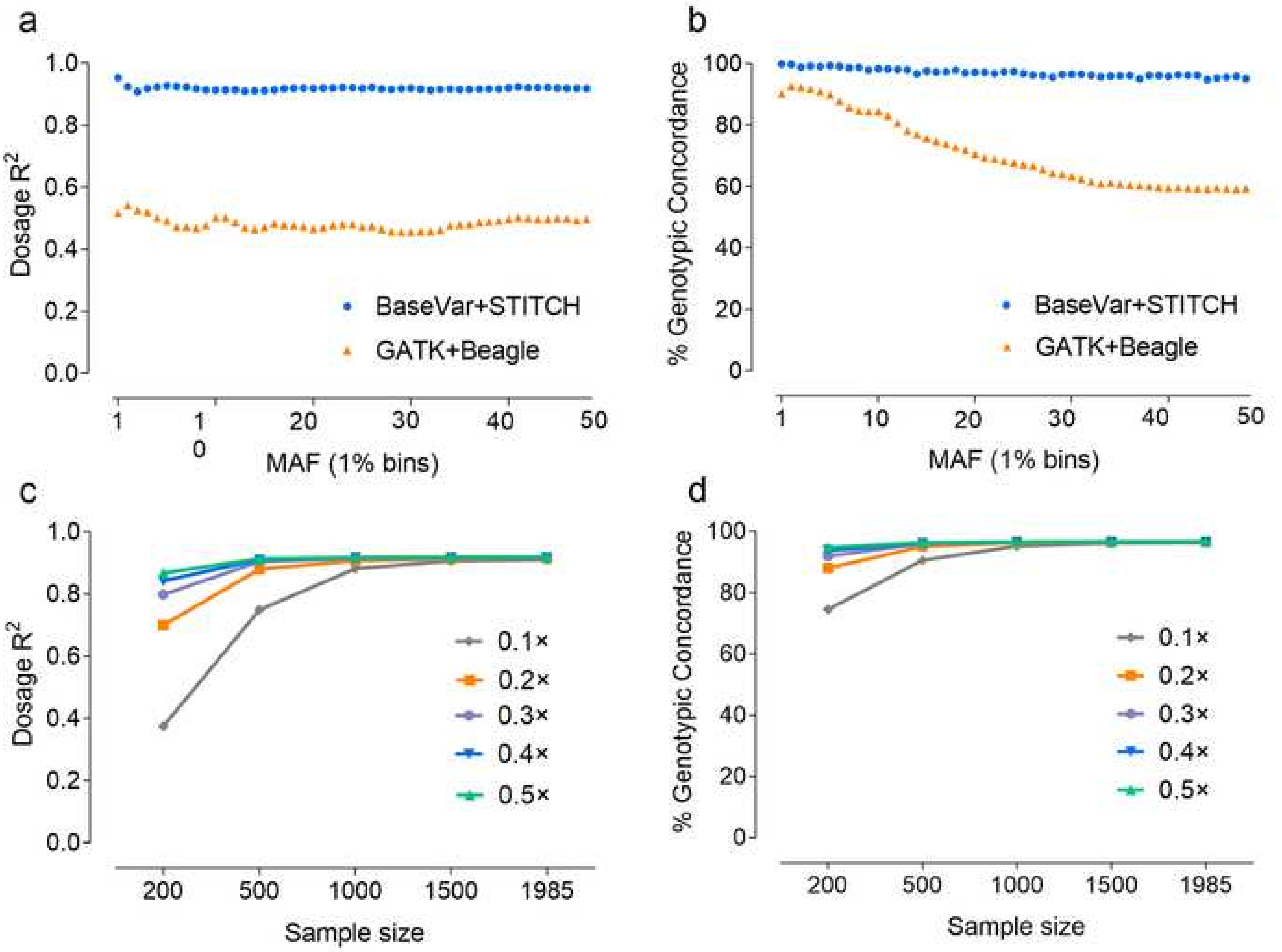

Figure 3

Click here to access/download;Figure;Figure 3.tif

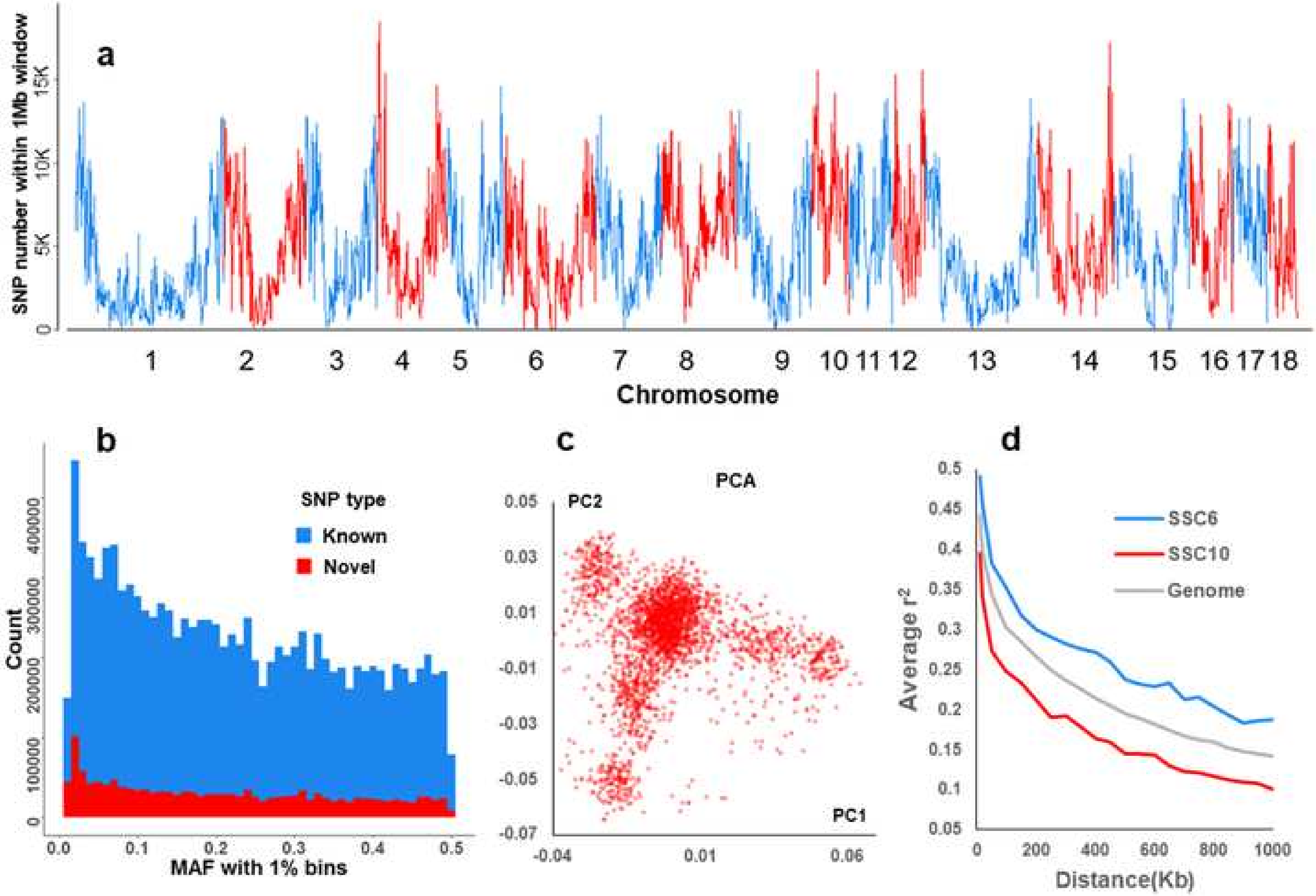

Figure 4

Click here to access/download;Figure;Figure 4.tif

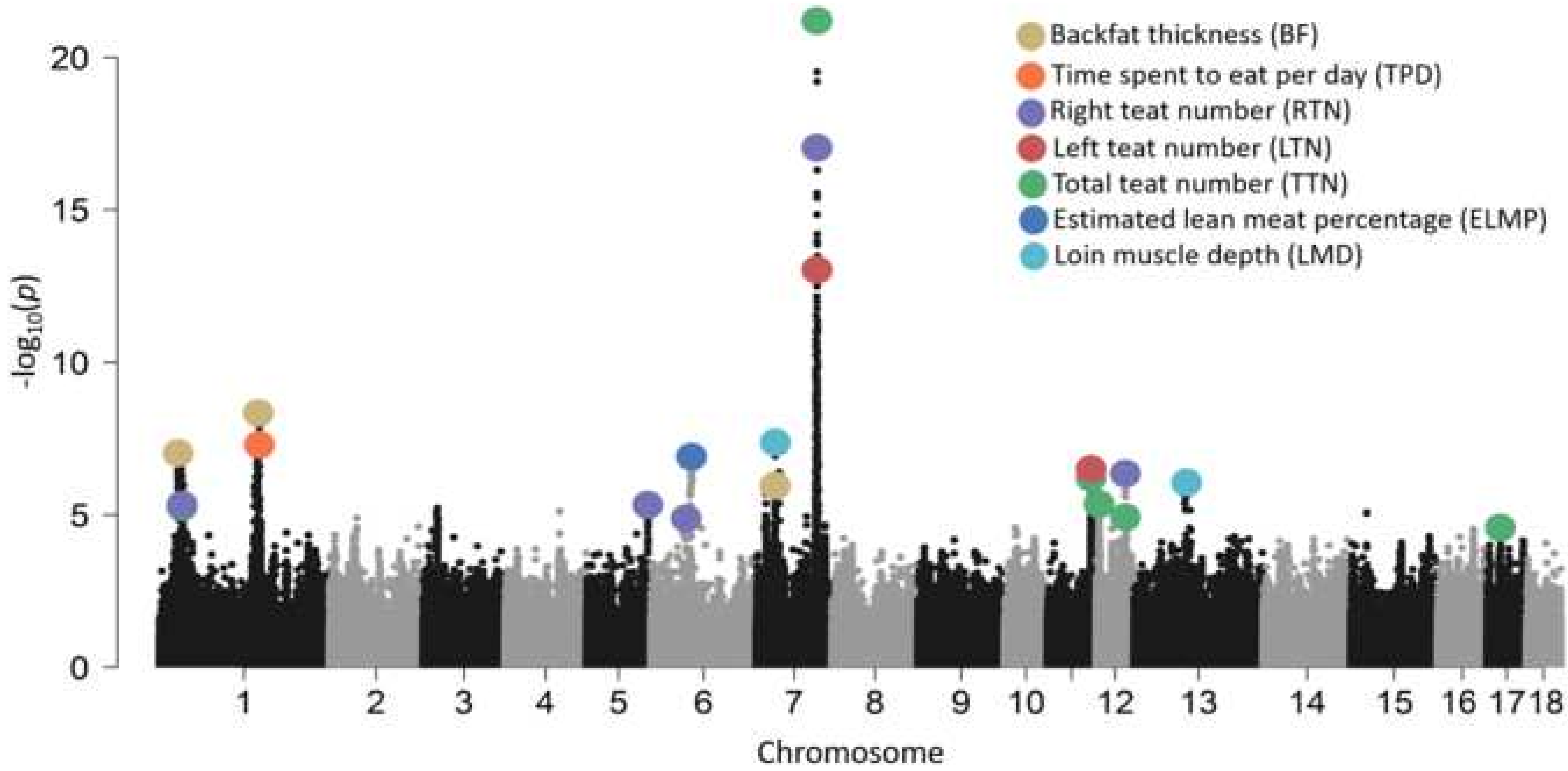

Figure 5

Click here to access/download;Figure;Figure 5.tif

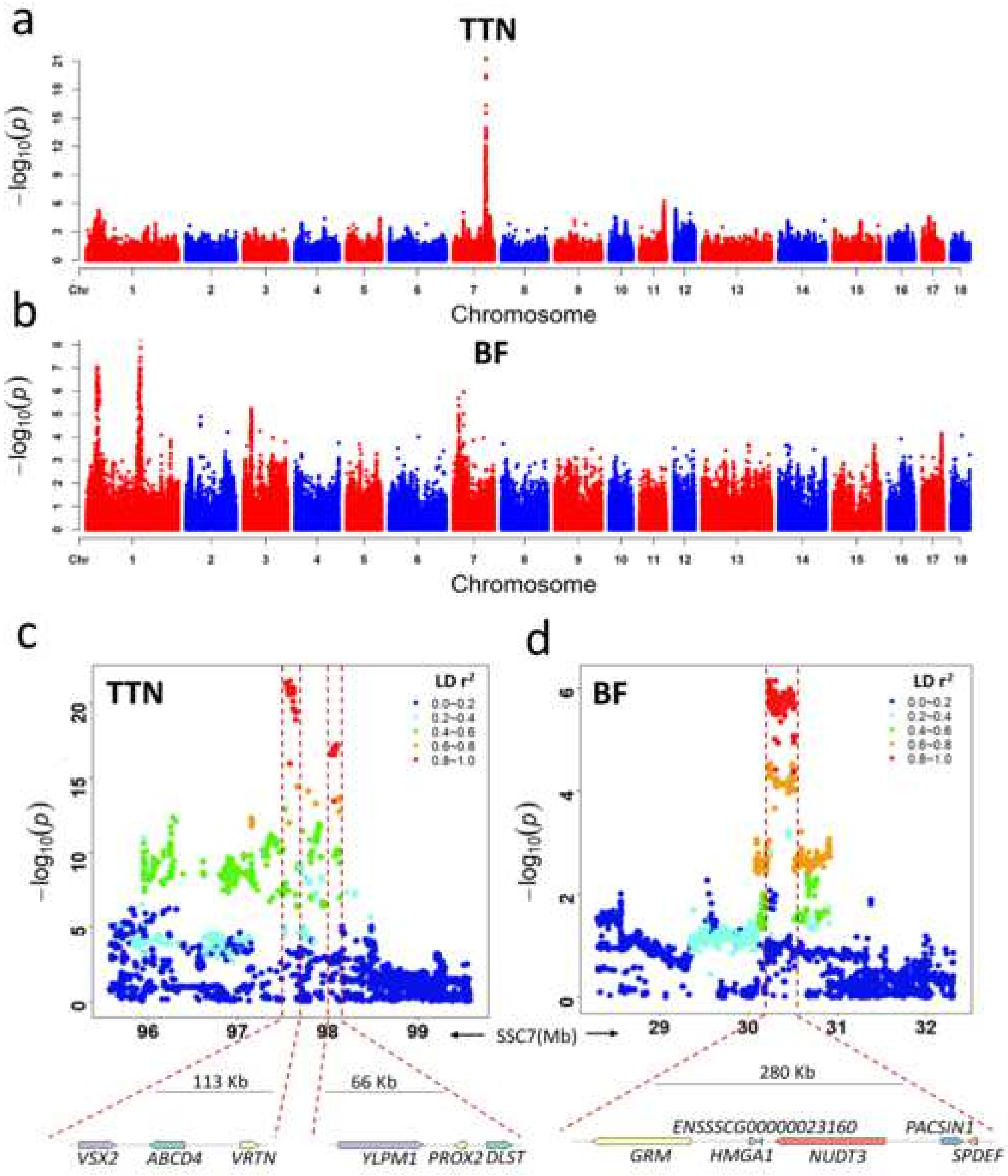

Figure 6

Click here to access/download;Figure;Figure 6.tif

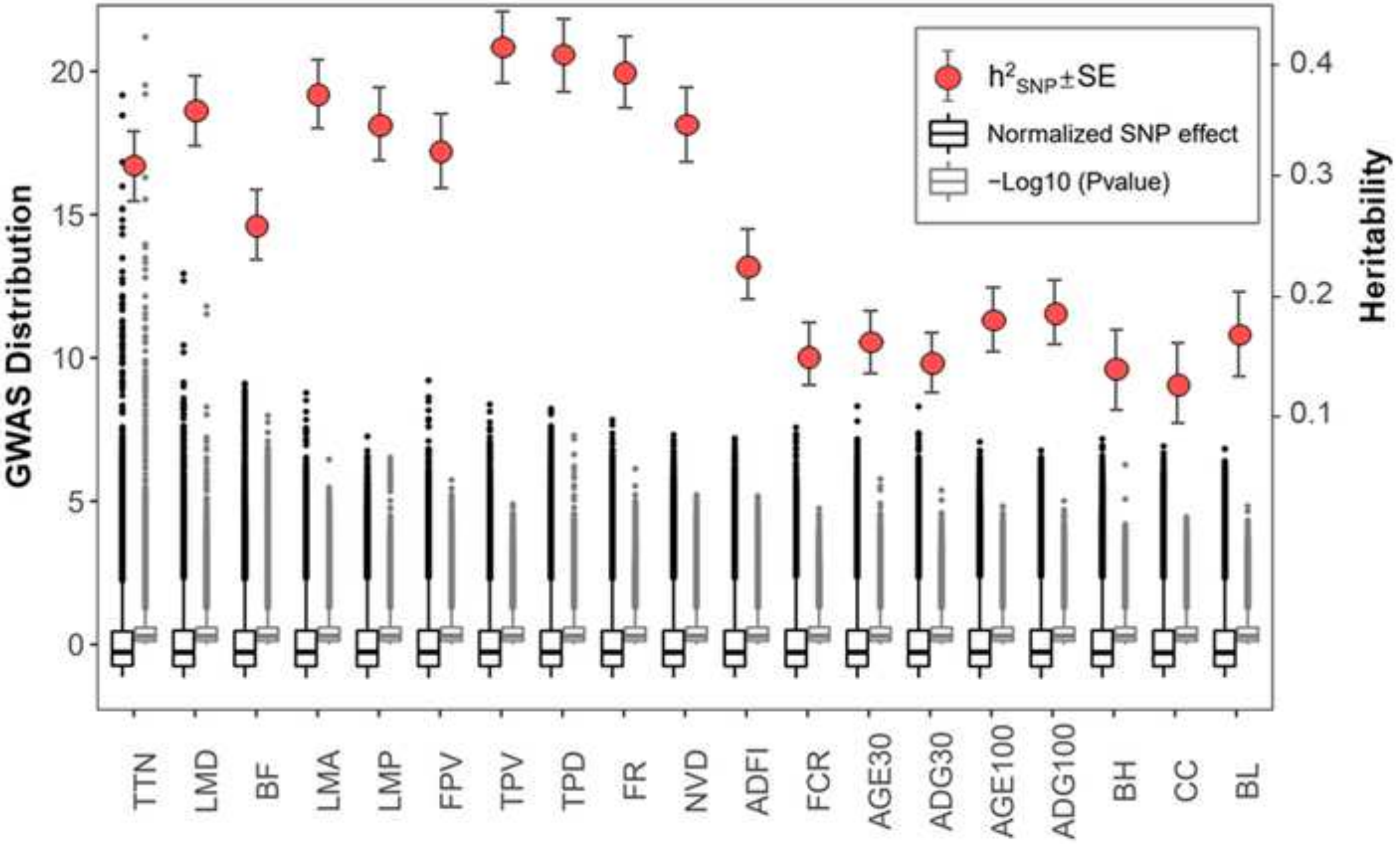

Supplementary Figure S1

Click here to access/download
Supplementary Material
Supplementary Figure 1.tif

Supplementary Figure S2

Click here to access/download
Supplementary Material
Supplementary Figure 2.tif

Supplementary Figure S3

Click here to access/download
Supplementary Material
Supplementary Figure 3.tif

Supplementary Figure S4

Click here to access/download
Supplementary Material
Supplementary Figure 4.tif

Supplementary Figure S5

Click here to access/download
Supplementary Material
Supplementary Figure 5.tif

Supplementary Figure S6

Click here to access/download
Supplementary Material
Supplementary Figure 6.tif

Supplementary Figure S7

Click here to access/download
Supplementary Material
Supplementary Figure 7.tif

Supplementary Figure S8

Click here to access/download
Supplementary Material
Supplementary Figure 8.tif

Supplementary Figure S9

Click here to access/download
Supplementary Material
Supplementary Figure 9.png

Supplementary Table S1

Click here to access/download
Supplementary Material
Supplementary Table S1.xlsx

Supplementary Table S2

Click here to access/download
Supplementary Material
Supplementary Table S2.xlsx

Supplementary Table S3

Click here to access/download
Supplementary Material
Supplementary Table S3.xlsx

Supplementary Table S4

Click here to access/download
Supplementary Material
Supplementary Table S4.xlsx

Supplementary Table S5

Click here to access/download
Supplementary Material
Supplementary Table S5.xlsx

Supplementary Table S6

Click here to access/download
Supplementary Material
Supplementary Table S6.xlsx

Supplementary Table S7

Click here to access/download
Supplementary Material
Supplementary Table S7.xlsx

Supplementary Table S8

Click here to access/download
Supplementary Material
Supplementary Table S8.xlsx

Supplementary Table S9

Click here to access/download
Supplementary Material
Supplementary Table S9.xlsx

Supplementary Table S10

Click here to access/download
Supplementary Material
SupplementaryTable S10.xlsx

Personal Cover

Click here to access/download;Personal Cover;Cover letter-GIGA.docx

Cover Letter

December 01, 2020

Dear Editor,

We would like to resubmit to ***GigaScience*** the modified manuscript entitled "Accelerated Deciphering of the Genetic Architecture of Agricultural Economic Traits in Pigs Using the Low Coverage Whole-genome Sequencing Strategy". We believe that this manuscript will make it interesting to general readers of your journal.

Domestication not only modified the economic important traits but also left a genetic signature that affects both the population diversity and genomic structure of domesticated farm animals. Fully elucidating the phenotypic diversity, revealing the genetic structure of the breeding population is the basis for precision breeding. Large-scale WGS and GWAS strategies had enable us to gain different perspectives which was not possible before. However, high depth sequencing in large cohorts is still prohibitively expensive, to develop a massively parallel low coverage sequencing method has become imperative.

Here, we report a Tn5-based, highly accurate, cost and time-efficient, low coverage sequencing (LCS) approach to perform sequencing on 2,869 Duroc boars at an average depth of 0.73×, which identify 11.3 M SNPs throughout the genome. Base on the whole genome sequencing strategy, the high-resolution genome-wide association study (GWAS) detected 14 candidate quantitative trait loci (QTLs) in 7 of 21 important traits and provided a lot of worth points for further investigation. We also showed that the artificial selection alters genomes that affect important growth traits. Moreover, we explored the different traits with varies genetic architecture in depth, providing guidance for subsequent genetic improvement by genomic selection. The LCS strategy, together with the unprecedented capacity of NGS allows the cost-effective and large-scale genome analysis with industrial-scale efficiency, and we are also confident that it will be a universal strategy to meet the needs for the genomic study and breeding of both animals and plants.

All of the sequencing raw data in this study have been deposited into NCBI with accession number PRJNA681437, the variance data as VCF file will be available via GIGADB. The data will be shared publicly without restrictions in case of acceptance.

We confirm that this manuscript has not been published elsewhere and is not under consideration by another journal, and all authors declare that they have no competing interests.

Thank you for your consideration. We look forward to hearing from you at your earliest convenience.

Xiaoxiang Hu

The State Key Laboratory for Agro-biotechnology, China Agricultural University, Beijing, PRC,100193

Email: huxx@cau.edu.cn
