# Supplementary material for: Accelerated deciphering of the genetic architecture of agricultural economic traits in pigs using a low-coverage whole-genome sequencing strategy
Source: Gigascience. 2021 Jul 20;10(7):giab048. doi: 10.1093/gigascience/giab048 (PMC8290195; doi:10.1093/gigascience/giab048)
Supplement: giab048_GIGA-D-20-00354_R2 [file giab048_giga-d-20-00354_r2.pdf]

GigaScience

Accelerated deciphering of the genetic architecture of agricultural economic traits in pigs using the low coverage whole-genome sequencing strategy

--Manuscript Draft--

**Manuscript Number:**	GIGA-D-20-00354R2
**Full Title:**	Accelerated deciphering of the genetic architecture of agricultural economic traits in pigs using the low coverage whole-genome sequencing strategy
**Article Type:**	Research
**Funding Information:**	National Transgenic Grand Project (2016ZX08009003-006) — Prof. Xiaoxiang Hu<br>948 Program of the Ministry of Agriculture of China (2012-G1(4)) — Prof. Xiaoxiang Hu<br>Science and Technology Innovation Strategy Projects of Guangdong Province (2019B020203002) — Prof. Xiaoxiang Hu<br>Guangdong Academician Workstation (2011A090700016) — Prof. Ning Li
**Abstract:**	Background : Uncovering the genetic architecture of economic traits in pigs is important for agricultural breeding. However, high-density haplotype reference panels are unavailable in most agricultural species, limiting accurate genotype imputation in large populations. Moreover, the infinitesimal model of quantitative traits implies that weak association signals tend to be spread across most of the genome, further complicating the genetic analysis. Hence, there is a need to develop new methods for sequencing large cohorts without large reference panels. Results : We describe a Tn5-based highly accurate, cost- and time-efficient, low coverage sequencing (LCS) method to obtain 11.3 M whole genome SNPs in 2,869 Duroc boars at an average depth of 0.73×. Based on these SNPs, a genome-wide association study (GWAS) was performed resulting in 14 quantitative trait loci (QTLs) for seven of 21 important agricultural traits in pigs. These QTLs harbour genes, such as ABCD4 for total teat number and HMGA1 for back fat thickness and provided a starting point for further investigation. The inheritance models of the different traits varied greatly. Most follow the minor-polygene model, but this can be attributed to different reasons, such as the shaping of genetic architecture by artificial selection for this population and sufficiently interconnected minor gene regulatory networks. Conclusions : GWAS results for 21 important agricultural traits identified 14 QTLs/genes and showed their various genetic architectures, providing promising guidance for genetic improvement harnessing genomic features. The Tn5-based LCS method can be applied to large-scale genome studies for any species without good reference panel and can be widely used for agricultural breeding.
**Corresponding Author:**	Xiaoxiang Hu<br>China Agricultural University<br>CHINA
**Corresponding Author Secondary Information:**	
**Corresponding Author's Institution:**	China Agricultural University
**Corresponding Author's Secondary Institution:**	
**First Author:**	Ruifei Yang, Ph.D.
**First Author Secondary Information:**	
**Order of Authors:**	Ruifei Yang, Ph.D.<br>Xiaoli Guo<br>Di Zhu

Powered by Editorial Manager® and ProduXion Manager® from Aries Systems Corporation

	Cheng Tan, Ph.D.
	Cheng Bian
	Jiangli Ren
	Zhuolin Huang
	Yiqiang Zhao, Ph.D.
	Gengyuan Cai, Ph.D.
	Dewu Liu, Ph.D.
	Zhenfang Wu, Ph.D.
	Yuzhe Wang, Ph.D.
	Ning Li, Ph.D.
	Xiaoxiang Hu, Ph.D.
**Order of Authors Secondary Information:**	
**Response to Reviewers:**	Dear editor and reviewers,<br><br>We would like to sincerely thank your helpful comments. We hereby re-submit a revised version of our manuscript entitled "Accelerated deciphering of the genetic architecture of agricultural economic traits in pigs using the low coverage whole-genome sequencing strategy" for publication in GigaScience.<br><br>We have addressed all comments of editor and reviewer2. For clarity, here we answered the questions point by point. If you have any questions, please just let me know. I am greatly looking forward to your response.<br><br>Sincerely,<br>Xiaoxiang Hu, Professor, Ph.D.<br>College of Biological Sciences<br>China Agricultural University<br>No.2 Yuanmingyuan West Road, Beijing 100193, P. R. China<br>Phone: +86-10-62733394<br>E-mail: huxx@cau.edu.cn<br><br><br>The response to comments from Editor<br>1.Please note that you can indicate a maximum of two "corresponding authors" (but you can also indicate a role for a "senior author", if you wish).<br>Response: The two corresponding authors are Dr. Yuzhe Wang and Dr. Zhenfang Wu. Professor Xiaoxiang Hu as the senior author of this article.<br><br>2. Our data curators will contact you shortly to prepare the supporting data for release via GigaDB. Please include a citation to your upcoming GigaDB dataset (including the DOI link) to your reference list, and cite this in the data availability section and elsewhere in the manuscript, where appropriate.<br>Response: This GigaDB dataset has been cited as "An archival copy of the code and supporting data is available via the GigaScience repository, GigaDB [75]."<br><br>The response to comments from Reviewer 2<br><br>Page 2, line 8: Change "We described a" to "We describe a"<br>Response: This has been corrected.<br><br>Page 2, lines 10-13: I suggest changing this long sentence to the following two sentences: "Based on these SNPs, a genome-wide association study (GWAS) was performed resulting in 14 quantitative trait loci (QTLs) for seven of 21 important agricultural traits in pigs. These QTLs harbour genes, such as ABCD4 for total teat number and HMGA1 for back fat thickness and provided a starting point for further investigation."<br>Response: This has been corrected.

	Page 4, line 28: I suggest changing this sentence to: "The results generated by the BGI platform had a smaller number of PCR duplicates (2.23%), a higher number of index reads (97.10%),……."<br>Response: This has been corrected.<br><br>Page 5, line 3: Insert "the" between "and" and "majority"<br>Response: This has been corrected.<br><br>Page 6, line 12: "Fluidigm" instead of "Fludigm"<br>Response: This has been corrected.<br><br>Page 7, line 2; Replace "in all" by "for all" or by "using all"<br>Response: This has been corrected.<br><br>Page 7, line 17-18: "We further studied the high level of LD, and found that it could be a consequence of long-term strong natural or artificial selection." I don't agree with this statement. The high LD is the result of the small effective population size used for these breeding lines.<br>Response: We have deleted this sentence.<br><br>Page 8, line 13: Replace "each" by "individual"<br>Response: This has been corrected.<br><br>Page 8, line 15: "We first focused on QTLs that could be narrowed". The authors do not show how this is done. Based on what information could these QTL be narrowed?<br>Response: We have added detailed analysis methods and parameters in the method-"Genome-wide association and Heritability estimation" section: "Once a QTL had been mapped using the tagging SNPs and exceeded the FDR threshold, association was re-calculated with all imputed SNPs (from the 11.3 M set) in a 20 Mb window around the peak using the same mixed model. The definition of a fine-mapped candidate QTL interval was based on the linkage disequilibrium (LD) level between the most significant SNP and all flanking sites, where the boundary was verified when the LD was no more than 0.8".<br><br>Page 10, line 3: Change "which in turn led to the result of no significant QTL." To "which in turn resulted in no significant QTL."<br>Response: This has been corrected.
**Additional Information:**	
**Question**	**Response**
Are you submitting this manuscript to a special series or article collection?	No
**Experimental design and statistics**<br><br>Full details of the experimental design and statistical methods used should be given in the Methods section, as detailed in our Minimum Standards Reporting Checklist. Information essential to interpreting the data presented should be made available in the figure legends.<br><br>Have you included all the information requested in your manuscript?	Yes

**Resources**<br><br>A description of all resources used, including antibodies, cell lines, animals and software tools, with enough information to allow them to be uniquely identified, should be included in the Methods section. Authors are strongly encouraged to cite Research Resource Identifiers (RRIDs) for antibodies, model organisms and tools, where possible.<br><br>Have you included the information requested as detailed in our Minimum Standards Reporting Checklist?	Yes
**Availability of data and materials**<br><br>All datasets and code on which the conclusions of the paper rely must be either included in your submission or deposited in publicly available repositories (where available and ethically appropriate), referencing such data using a unique identifier in the references and in the “Availability of Data and Materials” section of your manuscript.<br><br>Have you have met the above requirement as detailed in our Minimum Standards Reporting Checklist?	Yes

Manuscript

Click here to access/download;Manuscript;Manuscript-GIGA-0520-forproduction.docx

Accelerated deciphering of the genetic architecture of agricultural economic traits in pigs using the low coverage whole-genome sequencing strategy

Ruifei Yang[1†], Xiaoli Guo[1†], Di Zhu[1†], Cheng Tan[3], Cheng Bian[1], Jiangli Ren[1], Zhuolin Huang[1], Yiqiang Zhao[1], Gengyuan Cai[3], Dewu Liu[3], Zhenfang Wu[3]*, Yuzhe Wang[1,2]*, Ning Li[1] and Xiaoxiang Hu[1#]

[1]State Key Laboratory of Agrobiotechnology, College of Biological Sciences, China Agricultural University, Beijing, China.

[2]National Research Facility for Phenotypic and Genotypic Analysis of Model Animals (Beijing), China Agricultural University, Beijing, China.

[3]National Engineering Research Center for Breeding Swine Industry, South China Agricultural University, Guangdong, China.

*Correspondence address: State Key Laboratory of Agrobiotechnology, College of Biological Sciences, China Agricultural University, Beijing, 100193, P. R. China. Tel: ++86-010-62733394; E-mail: yuzhe891@cau.edu.cn, wzfemail@163.com.

†These authors have contributed equally and should be considered co-first authors.

Senior author. E-mail: huxx@cau.edu.cn

Abstract

Background: Uncovering the genetic architecture of economic traits in pigs is important for agricultural breeding. However, high-density haplotype reference panels are unavailable in most agricultural species, limiting accurate genotype imputation in large populations. Moreover, the infinitesimal model of quantitative traits implies that weak association signals tend to be spread across most of the genome, further complicating the genetic analysis. Hence, there is a need to develop new methods for sequencing large cohorts without large reference panels.

Results: We describe a Tn5-based highly accurate, cost- and time-efficient, low coverage sequencing (LCS) method to obtain 11.3 M whole genome SNPs in 2,869 Duroc boars at an average depth of 0.73×. Based on these SNPs, a genome-wide association study (GWAS) was performed resulting in 14 quantitative trait loci (QTLs) for seven of 21 important agricultural traits in pigs. These QTLs harbour genes, such as *ABCD4* for total teat number and *HMGA1* for back fat thickness and provided a starting point for further investigation. The inheritance models of the different traits varied greatly. Most follow the minor-polygene model, but this can be attributed to different reasons, such as the shaping of genetic architecture by artificial selection for this population and sufficiently interconnected minor gene regulatory networks.

Conclusions: GWAS results for 21 important agricultural traits identified 14 QTLs/genes and showed their various genetic architectures, providing promising guidance for genetic improvement harnessing genomic features. The Tn5-based LCS method can be applied to large-scale genome studies for any species without good reference panel and can be widely used for agricultural breeding.

Keywords: Low coverage sequencing; GWAS; genotyping; pig; genetic architecture; agricultural traits

Background

Genome-wide association studies (GWAS) have identified thousands of genetic variants associated with complex traits in humans and agricultural species [1, 2]. The mapping resolution relies on the density of genetic markers that can reveal linkage disequilibrium (LD) patterns in sufficiently large populations [3, 4]. Despite the declining cost of sequencing, it is still expensive for agricultural breeding studies to perform whole-genome sequencing of all individuals in a large cohort (thousands of individuals). In many scenarios, imputation-based strategies, which impute low-density panels to higher densities, offer an alternative to systematic genotyping or sequencing [5, 6]. Array-based genotype imputation is widely used in agricultural species [7, 8]. However, the imputation accuracy of this strategy depends crucially on the reference panel sizes and genetic distances between the reference and target populations. Hence, the unavailability of large reference panels and array designs for target populations in agricultural species limits the improvement offered by array-based genotype imputation [9, 10]. Inaccurate imputations influence the results of follow-up population genetic analyses.

Low-coverage sequencing (LCS) of a large cohort has been proposed to be more informative than sequencing fewer individuals at a higher coverage rate [11-13]. Sample sizes and haplotype diversity could be more critical than sequencing depth in determining the genotype accuracy of most segregating sites and increasing the power of association studies. Overall, LCS has been proven to have greater power for trait mapping than the array-based genotyping method in human studies [14]. To date, LCS-based genotype imputation has been employed in many studies using various populations and genotyping algorithms [15-19]. In particular, the STITCH imputation algorithm overcomes the barrier of the lack of good reference panels in non-human species and is even applicable in studies with extremely low sequencing depths [15, 20]. This is a promising approach for agricultural animals without large reference panels

and can be used in the areas of functional genetic mapping and genomic breeding. However, to date, no reports have been published on this.

Several large-scale whole-genome sequencing projects have been completed [21-25]. These projects were designed to identify the underlying mechanisms that drive hereditary diseases in humans, as well as for use in genomic selection in the breeding of agricultural species [26-28]. The infinitesimal model, which describes the inheritance patterns of quantitative traits appears to be successful [29, 30]; however, it is unclear how many genes play important roles in driving different kinds of complex traits. In addition, artificial selection provides a driving force for the rapid evolution of agricultural species, which further brings about the fixation of selection regions and differentials in the inheritance model. This process might produce a very different result for the same trait between studies due to the different genetic backgrounds of the research population. Therefore, care should be taken when determining the GWAS results for a specific population. Such information, which might be helpful for understanding the genetic mechanism of a complex trait, could be informative for further application of genomic selection in animal breeding.

In this study, we developed a new highly accurate, cost- and time-efficient LCS method to obtain high-density SNP markers for a large Duroc pig population [31]. By assessing 21 important agricultural traits in commercial pig herds, we performed genome-wide association and fine-mapping analyses with high resolution and compared the results of the inheritance model in depth. We also proved that artificial selection plays a significant role in altering the genetic architecture of agricultural animals, especially for loci that affect economic traits. The LCS strategy offers a powerful method for further agricultural breeding.

Data Description

A Tn5-based protocol was used to prepare sequencing libraries of each pig at a low cost (reagent cost: $2.60 /library) as described in the Materials and Methods section. The libraries were sequenced on the Illumina (PE 150 model, two libraries) and the BGI

platform (PE 100 model, 28 libraries) (Supplementary Table S1). The results generated by the BGI platform had a smaller number of PCR duplicates (2.23%), a higher number of good index reads (97.10%), and higher genome coverage (98.55%) than the Illumina dataset (10.82% PCR duplicates, 93.64% good index reads, and 98.50% genome coverage). Overall, the total output of the 2,869 boars approached 5.32 TB, and the majority (96.74%) of the reads were successfully mapped to the pig reference genome Sscrofa11.1. Each animal was sequenced at an average depth of 0.73 ± 0.17×. Moreover, high depth resequencing (n=37, selected from the 2,869 boars, average 15.15×/sample), SNP Array (n=42, GeneSeek Genomic Profiler Porcine 80K SNP Array, GGP-80) genotyping and Fluidigm IFC direct genotyping (n=191 for 16 SNP loci) were performed on the selected Duroc core boars of this population, and the results were used for downstream accuracy evaluation. The 21 associated phenotypes used in this study are shown in Table 1 and Fig. S1.

Analyses

Processing pipeline of the low-coverage strategy and accuracy evaluation

Traditional standard methods for SNP calling, such as those implemented in GATK and SAMtools, are mainly used in high-depth resequencing methods. However, due to the low depth of each base, erroneous SNPs and genotypes could be called using such methods, especially for the GATK HaplotypeCaller algorithm (single sample local de novo assembly) [32]. Hence, in this study, we mainly applied the BaseVar algorithm [33] to identify polymorphic sites and infer allele frequencies, and STITCH to impute SNPs. We also tested the performance of GATK (UnifiedGenotypeCaller)-Beagle algorithms in LCS data. The high-depth sequencing data and SNP chip (GGP-80) results on SSC18 were used as the gold standard for accuracy evaluation (Fig. 1 and Supplementary Table S2). Correlations (R^2) [34] between genotypes and imputed dosages and genotypic concordance (GC) were calculated to evaluate the genotyping accuracy. The initial screening of SSC18 with BaseVar identified 506,452 and 414,160 bi-allelic candidate polymorphic sites before and after quality control, respectively.

These sites were imputed using STITCH, and 322,386 SNPs were retained with a high average call rate (98.89% ± 0.59%) after quality control (imputation info score > 0.4, Hardy Weinberg Equilibrium *P* value > $1e^{-6}$). The SNPs detected by BaseVar/STITCH were mostly included (99.32%) in the GATK-Beagle set, which included 570,919 sites and contained 320,199 SNPs overlapping with the BaseVar/STITCH dataset. As a result, a relatively high-quality genotype set was acquired with less time consumption when K = 10 (the number of founders or ancestral haplotypes, Fig. S2). Fig. 2 shows that highly accurate genotypes were obtained using the BaseVar-STITCH pipeline compared with the high-depth sequencing result (R^2 = 0.919 and GC = 0.970) across all allele frequencies, which exceeded the method using GATK-Beagle (R^2 = 0.484 and GC = 0.709). Moreover, the BaseVar-STITCH results showed even higher GC concordance and R^2 values compared with the GGP-80 data (R^2 = 0.997 and GC = 0.990). Furthermore, direct genotyping (16 loci, 191 individuals) was carried out using the Fluidigm dynamic array IFC. The average GC was 0.991 compared with the BaseVar-STITCH data (Supplementary Table S3), which is as high as the aforementioned results. Taken together, these results suggest that BaseVar-STITCH pipeline is a suitable variant discovery and imputation method for the LCS strategy (Fig. 1).

Previous studies have demonstrated that low-depth sequencing of a large number of samples generally provides a better representation of population genetic variations compared to high-depth sequencing of a limited number of individuals. In this study, we examined the consequences of altering the sample size and sequence coverage in this population. For the 0.5× coverage using STITCH, a sample size above 500 had little impact on performance. At a 0.1× downsampled coverage, increasing the sample size to 1,985 led to a substantially improved performance (Fig. 2C and 2D). At 0.2× for 1,000 individuals, it was noteworthy that the results were only marginally poorer (R^2 = 0.908 and GC = 0.962) than using all sequencing data (Fig. 2C and 2D). In general, the total sequencing depth (population category) for one locus > 200× was shown to

guarantee the credibility of genotyping within the scope of this study, although the results consistently improved as sequencing depth/sample size increased.

Genetic architecture of the Duroc population

After strict parameter filtering in the pipeline (BaseVar-STITCH, Fig. 1), we retained 11,348,460 SNPs for all 2,797 Duroc pigs with high genotype accuracy, and the density corresponded to one SNP per 200 bp in the pig genome (Fig. 3A and Supplementary Table S4). Finally, the majority of the identified SNPs were located in intergenic regions (51.98%) and intronic regions (36.85%). The exonic regions contained 1.37% of the SNPs, including 0.14% missense SNPs. Among the discovered SNPs, 1,524,015 (accounting for 13.43% of all SNPs) were novel to the pig dbSNP database (data from NCBI: GCA_000003025.6 in June 2017). Both novel and known variants were found to have very similar minor allele frequency distributions across the whole genome, with an average minor allele frequency (MAF) of 0.225 (Fig. 3B). A principal component analysis (PCA) of all pigs showed that there was no distinct population stratification (Fig. 3C). The decay of LD with increasing distance was different among the chromosomes, of which the fastest and slowest decay rates occurred for SSC10 and SSC6, respectively. The average pairwise LD r^2 values fell to 0.20 at 500 Kb and to 0.14 at 1 Mb (Fig. 3D), providing the expected mapping resolution obtainable with this population.

. Tajima's D and diversity Pi were implemented to analyse selective sweep regions simultaneously, and only windows with an interquartile range of Tajima's D and diversity Pi of 1.5-fold in the whole genome were regarded as putative selection regions. In total, 24 putative fixed selective regions harbouring 281 genes were identified (Fig. S3). The regions displayed significant overrepresentation of genes involved in the sensory perception of smell (P = 6.41e^{-10}) (Supplementary Table S5), reflecting the importance of smell when scavenging for food during long periods of environmental adaptation. This result is consistent with a previous study that reported that genes associated with olfaction exhibit fast evolution in pigs [35]. We also observed a

significant enrichment of genes involved in the neurological system process (*P* = 8.64e^{-5}), hair cycle process (*P* = 0.004), and bone mineralisation (*P* = 0.040).

GWAS and identification of high-resolution mapping of QTLs

The 21 phenotypes used in this study are shown in Table 1. There was high correlation between traits of the same type (such as LMD, LMA and LMP; BH, BL and CC, Supplementary Table S6). We identified a subset of 258,662 SNPs that tagged all other SNPs with MAF >1% at LD r^2 <0.98 for the first round of GWAS (Supplementary Table S4). Fine-mapping was performed within 10 Mb of the SNPs to reach 5 genome-wide false discovery rate (FDR) significance threshold of 5%. Overall, we discovered 14 non-overlapping QTLs for the seven traits at a significance threshold of 5% (Fig. 4, Table 1, Fig. S4, and Fig. S5). The widths of all QTL intervals ranged from ~66 Kb to ~3.9 Mb. The intervals of five QTLs were more than 2 Mb in width (Supplementary Table S7). These QTLs were strongly influenced by the local LD levels of this population.

On average, individual QTL covered 13 protein-coding genes (ranging from zero–48) with a median of eight genes. The distribution of the number of genes in a QTL is shown in Supplementary Table S7. We first focused on QTLs that could be narrowed, since these loci could provide a starting point for functional investigations. Of the 14 non-overlapping loci identified in this study, seven QTLs could be further narrowed to a small number of genes (one to nine genes) (Fig. 5 and Fig. S6). Here, we highlight two important QTLs on SSC7.

The QTL on SSC7 with a major effect on the total teat number (TTN) has been widely identified in several commercial breeding lines and hybrids [36-38]. Our GWAS results show a strong QTL for TTN in the same region, explaining most of the phenotypic variance compared with other QTLs (Supplementary Table S7), reflecting the major effect of this locus. (Fig. 4). Fine-mapping revealed two narrow LD blocks (SSC7:97.56–97.65 Mb and 98.06–98.10 Mb), containing four candidate genes (*ABCD4*, *VRTN*, *PROX2*, and *DLST*) (Fig. 5 and Fig. S6). We noticed that the most significant locus (SSC7:97,581,669, *P* = 3.29e^{-22}) was detected in the region of *ABCD4*

gene, and one missense SNP in *ABCD4* had the most severe impact with the largest decrease in protein stability (Supplementary Table S9), suggesting that *ABCD4* may be the most likely causal gene. In addition, four missense variants were discovered in *PROX2*, which was the vertebrate homolog of the homeodomain-containing protein, Prospero, that may be involved in cell fate determination and body plan establishment in *Drosophila melanogaster*[39]. Previous studies have reported that *PROX2* could be the causal gene [31, 40].

For the carcass traits, we identified six QTLs (Table 1 and Supplementary Table S7), in which a common narrowed QTL region on SSC7 of 30.24–30.52 Mb was identified to be significantly associated with back fat thickness (BF) and loin muscle depth (LMD) (Fig. 5 and Fig. S6). Among the QTLs associated with BF and LMD, the narrowed QTL on SSC7 was found to make the greatest contribution to heritability, indicating that this was the location of the major genes in the region (Table 1 and Fig. 5). In this region (Supplementary Table S7), *HMGA1* is a promising candidate gene associated with growth, carcass, organ weight, and fat metabolism, as it has been reported to be involved in a variety of genetic pathways regulating cell growth and differentiation, glucose uptake, and white and brown adipogenesis [41-45].

Heritability and pattern of QTL effects

To assess how much of the heritability can be explained by the detected QTLs, we estimated the effect size of the overall decreased proportion of heritability by using significant SNPs distributed in these QTLs as fixed effects. Seven of the 21 traits (TTN, LTN, RTN, BF, LMD, LMP, and TPD) exhibited medium to high heritability-major QTL effect (1.08 to 8.86%) profile (Table 1 and Figure 6). Among them, TTN showed the highest single-QTL effect and the most discrete distribution. The other six traits were explained by multiple QTLs, but the total effect was significantly lower than that of TTN. These results showed the differential genetic architecture of the gradual transition from qualitative-like traits to quantitative traits.

Few QTLs were detected for other traits, and most of them could be attributed to the typically small effect sizes of individual mutations, thousands of which contribute

to the total observed genetic variation but did not reach the significant level for a typical complex trait (such as body size measurement and feed intake traits). It is noteworthy that the heritability of growth traits, such as the average daily gain 30-100 kg (ADG100) and age to 100 kg daily weight (AGE100) were lower than those of other populations [46, 47] which in turn resulted in no significant QTL. To account for this, we hypothesized that the major QTL effect may be obscured by rare mutations under strong artificial selection. We searched the candidate loci of growth traits in the pig QTL database (https://www.animalgenome.org/cgi-bin/QTLdb/SS/index) as well as the corresponding previous reports [46, 48-51].We identified 51 sites associated with growth traits distributed on 18 chromosomes with low MAF (< 0.05) in our population. However, 151 previously-reported candidate sites were not identified as polymorphism in this study (Supplementary Table S8). The sequencing depths of these sites exceeded 2,100×, proving that these sites were completely fixed in our population with the same alleles as in the reference genome. This result reflected the long-term artificial selection history for growth traits of this commercial Duroc population, and explained the decreased heritability and major QTLs.

Discussion

To our knowledge, we have generated the largest whole genome sequencing (WGS) genotyping dataset for the Duroc population to date, containing 11 million markers from 2,797 pigs. We expanded the candidate causal mutations for multiple pig traits, and demonstrated the efficacy of genetic fine-mapping utilizing low-coverage sequencing in animal populations without reference panels. Further, we compared the heritability and inheritance models for each trait, providing a starting point for functional investigations. Our study indicated that the LCS method could have widespread usage in high-resolution GWAS for any genetic or breeding population, or even for applications in genomic prediction.

Our study identified an optimal design, taking into account the imputation algorithm, number of samples, and sequencing depth. The BaseVar-STITCH pipeline allows the

GC to be higher than 0.96 when the sample size is 1000 at a sequencing depth of 0.2× (200× at the population level) without large reference panels. This GC value is significantly higher than that in other studies with small sample sizes with a high sequencing depth or array-based genotype imputation [7, 9] We also found that genotype accuracy was more sensitive to the sample size than the sequencing depth. Hence, the results demonstrated that low-coverage designs are more powerful than deep sequencing of fewer individuals for animal sequencing studies, since a large sample size can cover all local haplotypes of the study population more effectively. This method has high accuracy, even in large-scale human studies with the most complex population structure [33], further showing that a sufficient sample size will ensure that the method has a broad spectrum of applicability in all agricultural species or breeding populations.

Increasing marker density has been proposed to have the potential to improve the power of GWAS and the accuracy of genomic selection (GS) for quantitative traits [52]. First, the whole-genome LCS data gave the best accuracy for GWAS, as it can capture more recombination events than SNP chips or target sequencing methods such as genotyping by sequencing (GBS) [31], and most causal or causal-linked mutations that underlie a trait are expected to be included. Second, many studies have reported the impact of WGS data on the accuracy of genomic predictions [52-54]; however, the conclusions have been quite divergent. The limited improvement of the genetic relationship matrices for WGS data compared with the SNP chip is the major reason for the lack of improvement in genomic prediction. In addition, while most researchers may prefer to impute SNP chip genotypes using limited WGS data, some erroneous SNPs may be introduced and further adversely affect the performance of genomic prediction, since limited haplotype architecture would be obtained using small-scale WGS data. Our method improved the accuracy of imputation, especially in large studies without a good reference panel and multibreed genomic predictions, widening make the application of genome selection. Third, significantly improved GS results were observed when SNPs were preselected from the sequenced data with prior information

and an optimized genomic prediction method considering genomic features (*e.g.* GFBLUP [55, 56]. Thus, we could select different useful tag-SNPs for various traits with different genetic architectures using the high-density genetic map built by LCS data to optimize the genomic selection model in the future. Fourth, in practical applications, the haplotype reference panel can accommodate new haplotypes due to recombination at any time, thus solving the issue of a decrease in prediction accuracy over generations. Our data cover the sites of various SNP chips well because the genome coverage exceeds 98.36%, and it is competitive with arrays in terms of cost and SNP density. In addition, we applied GTX, which is an FPGA-based hardware accelerator platform [57], to perform the alignments, and ~3,000 alignments were accomplished in two days. Then, genotyping and imputation could be achieved on the cluster server or even on a cloud server in a single day, thus resolving the accuracy and timeliness of genomic prediction.

Recent swine breeding has prompted the accumulation of beneficial genetic variations at a more rapid rate, especially for some economic trait loci [58, 59]. This study used a typical commercial population, that exhibits a high level of LD and number of selective regions under strong artificial selection. Thus, we presented a joint analysis of GWAS and selective sweep of this Duroc population to comprehensively extract more functional genes and genomic features. We detected 136 candidate genes (Supplementary Table S10) in 14 QTLs associated with seven traits, and highlighted important roles, such as *ABCD4* for total teat number and *HMGA1* for back fat thickness. A large number of fixed or nearly-fixed loci have been found to be associated with ADG, AGE, and FCR, which explained the missing QTL by GWAS, and reflected the growth-related selection index process exactly. We also detected 24 putative fixed selective regions harbouring a series of genes enriched for sensory perception and neurological system processes. It has been widely reported that olfactory receptor genes may not only reflect adaptation to different environments [60] but might also act as a species barrier by affecting mate choice [61]. Several studies have reported an overrepresentation of genes with gene ontology (GO) terms related to neuronal

development and neurological regulation [60, 62], which could be related to the complex genetic background of traits such as behaviour and increased tameness.

Fewer QTLs with significant SNPs were detected in feeding behaviour traits and body size measurements than in teat number and carcass traits. These observations are interpreted in a paradigm in which complex traits are driven by an accumulation of weak regulatory effects on the large genes and regulatory pathways [63-65], i.e. 'infinitesimal model'. This model motivated us to aggregate hits to identify key pathways and processes. In particular, the feeding behaviour traits exhibited high heritability-few QTL effect profiles. We combined related genes obtained from the top 100 loci from the GWAS of the six feed intake traits. Gene-set enrichment analysis based on the obtained 281 genes showed that neural development or neural activity related functions, such as astrocyte differentiation (P = 8.61e^{-5}), cognition (P = 0.002), learning (P = 0.002), and glial cell differentiation (P = 0.003), were significantly enriched (Fig. S7 and Supplementary Table S11). The KEGG pathway analysis also showed that the nervous system processes were significantly enriched (Fig. S8 and Supplementary Table S12), including the neurotrophin signaling pathway (P = 0.015) (Fig. S9) and GABAergic synapse (P = 0.021). This finding suggests that pig-feeding behaviour involves complex traits that are affected by the regulation of the nervous system, leading to the stimulation of appetite. The current breeding schedule of this commercial population has been successful, especially in terms of improving growth traits. The next stage should focus on the use of genomic selection strategies for 'infinitesimal traits' with high heritability but no major QTL, such as feeding behaviour traits.

In conclusion, we developed a Tn5-based, highly accurate, cost- and time-efficient LCS method to obtain whole genome SNP markers in a large Duroc population. GWAS results for 21 important agricultural traits identified tens of important QTLs/genes and showed their various genetic architectures, providing promising guidance for further genetic improvement harnessing genomic feature.

Potential Implications

The present work advances our understanding of the genetic architecture of quantitative traits and suggests a direction for future application of genomic information in pig breeding. We expect that our method could be applied to large-scale genome studies for any species without a good reference panel, especially for agricultural species that have important economic value. The rapid accumulation of data will significantly improve many bottlenecks in the current genome research, and will combine multi-omics information and artificial intelligence algorithms to contribute to decipher the genetic and regulatory mechanisms behind complex traits.

Methods

Animals, phenotyping, and DNA Extraction

The Duroc boars used for this study were born from September 2011 to September 2013. All boars were managed on a single nucleus farm in a commercial company, which enduring strong artificial selection for many years. The associated phenotype data used in this study included back fat thickness at 100 kg (BF), loin muscle area at 100 kg (LMA), loin muscle depth at 100 kg (LMD), lean meat percentage at 100 kg (LMP), average daily gain (0-30 kg and 30-100 kg) (ADG30 and ADG100), age to 30 kg and 100 kg daily weight (AGE30 and AGE100), body length (BL), body height (BH), circumference of cannon bone (CC), feed conversion ratio (FCR), average daily feed intake (ADFI), number of visits to feeder per day (NVD), time spent to eat per day (TPD), time spent to eat per visit (TPV), feed intake per visit (FPV), feed intake rate (FR), left teat number (LTN), right teat number (RTN), and total teat number (TTN). The phenotype TTN data were acquired from Tan's study [31]. In detail, the number of left and right teats of each pig were recorded within 48 h after birth, and only normal teats were counted. The total teat number in this study was the sum of normal left and right teats. Body weights were recorded at birth and at the beginning (30 ± 5 Kg) and the end (100 ± 5 Kg) of the experiment. The ADG was calculated as the total weight

gain over this time, divided by the number of days. The ages at which the pig reached 30 Kg and 100 Kg were recorded as AGE30 and AGE100 respectively. BF, LMD, LMA, and LMP were measured over the last three to four ribs using b-ultrasound-scan equipment when the weight of pigs reached 100 ± 5 Kg (Aloka SSD-500). Feeding behaviors including the time taken, duration, feed consumption, and weight of each pig were recorded at every visit by the Osborne FIRE Pig Performance Testing System (Kansas, American). The ADFI of each animal was obtained by dividing the total feed intake during the test by the number of days of the test period. The following feeding behavior and eating efficiency traits were defined and calculated for each boar: ADFI (Kg/day), TPD (min), NVD, TPV (= TPD/NVD, %), FPV (Kg), FR (= DFI/TPD, g/min), and FCR (=ADFI/ADG). The phenotypic values nearly all followed a normal distribution (Fig. S1).

Genomic DNA was extracted from the ear tissue using a DNeasy Blood & Tissue Kit (Qiagen 69506), assessed using a NanoDrop, and checked in 1% agarose gel. All samples were quantified using a Qubit 2.0 Fluorometer and then diluted to 40 ng/ml in 96-well plates.

Tn5 Library generation and sequencing

Equal amounts of Tn5ME-A/Tn5MErev and Tn5ME-B/Tn5MErev were incubated at 72 ℃ for 2 minutes and then placed on ice immediately. Tn5 (Karolinska Institute, Sweden) was loaded with Tn5ME-A+rev and Tn5ME-B+rev in 2× Tn5 dialysis buffer at 25 ℃ for 2 h. All linker oligonucleotides were the same as in a previous report [66].

Tagmentation were carried out at 55 ℃ for 10 minutes by mixing 4 μl 5×TAPS-$MgCl_2$, 2 μl of dimethylformamide (DMF) (Sigma Aldrich), 1 μl of the Tn5 pre-diluted to 16.5 ng/μl, 50 ng of DNA, and nuclease-free water. The total volume of the reaction was 20 μl. Then, 3.5 μl of 0.2% SDS was added, and Tn5 was inactivated for another 10 min at 55 ℃.

KAPA HiFi HotStart ReadyMix (Roche) was used for PCR amplification. The primers were designed for MGI sequencers, with the reverse primers containing 96 different index adaptors to distinguish individual libraries. The PCR program was as

follows: 9 min at 72 ℃, 30 sec at 98 ℃, and then 9 cycles of 30 sec at 98 ℃, 30 sec at 63 ℃, followed by 3 min at 72 ℃. The products were quantified by Qubit Fluorometric Quantitation (Invitrogen). The groups of 96 indexed samples were pooled with equal amounts (Supplementary Table S13).

Size selection was performed using AMPure XP beads (Beckmann), with a left side size selection ratio of 0.55× and a right side size selection ratio of 0.1×. The final libraries were sequenced on 2 lanes of MGISEQ-2000 to generate 2×100 bp paired-end reads or on 1 lane of BGISEQ-500 to generate 2×100 bp paired-end reads.

Genotype data obtained using high depth sequencing and SNP chip

We sequenced 37 out of the total 2,869 pigs using the Hiseq X Ten system at a high depth of 15.15×. GTX by the Genetalks company, a commercially available FPGA-based hardware accelerator platform, was used in this study for both mapping clean reads to the Sscrofa11.1 reference genome (ftp://ftp.ensembl.org/pub/release-99/fasta/sus_scrofa/dna/) and variant calling. The alignment process was accelerated by FPGA implementation of a parallel seed-and-extend approach based on the Smith–Waterman algorithm, while the variant calling process was accelerated by FPGA implementation of GATK HaplotypeCaller (PairHMM) [67]. GATK multi-sample best practice was used to call and genotype SNPs for the 37 pigs, and the SNPs were hard filtered with a relatively strict option “QD < 10.0 || ReadPosRankSum < -8.0 || FS > 10.0 || MQ<40.0”.

We also selected 42 individuals who were included in the LCS dataset and genotyped using the GeneSeek Genomic Profiler Porcine 80K SNP Array and obtained 68,528 SNPs across the whole genome. The genotypes of the sex chromosomes were excluded from this study, and after quality control (genotype call rate > 0.95), 47,946 SNPs remained. We retained 45,308 SNPs that overlapped with the LCS dataset to evaluate the genotypes from the LCS strategy.

Low coverage sequencing data analyses

Sequencing reads from the low coverage samples were mapped to the Sscrofa11.1 reference genome using GTX-align, which includes a step that involves marking PCR duplicates. The indel realignment and base quality recalibration modules in GATK were applied to realign the reads around indel candidate loci and to recalibrate the base quality. The average running time from a fastq file to a bam file was about 3 min for each sample in this study. Variant calling was done using the BaseVar and hard filtered with EAF >= 0.01 and a depth greater than or equal to 1.5 times the interquartile range. The detailed BaseVar algorithm that was used to call SNP variants and estimate allele frequency was described in a previous report [33]. We used STITCH to impute genotype probabilities for all individuals. The key parameter K (number of ancestral haplotypes) was decided based on the tests in SSC18. Results were filtered with an imputation info score > 0.4 and a Hardy Weinberg Equilibrium (HWE) *P* value > $1e^{-6}$. After quality control, 2,797 individuals with genotype data were obtained. Two validation actions were taken to calculate the accuracy of imputation. The first parameter was genotypic concordance (GC), which was calculated as the number of correctly-imputed genotypes divided by the total number of sites. Another parameter was the allele dosage R^2, which was described in a previous report [34]. The SNPEff program [68] was used to annotate the variants.

Population genetics analysis

A subset of 258,662 SNPs that tagged all other SNPs with MAF > 1% at LD $r^2 < 0.98$ and a call rate of >95% were retained for downstream analysis. PCA clustering analyses were performed using the GCTA software [69]. The average heterozygosity rate and MAF were obtained using the vcftools program [70]. Tajima's D [71] and diversity Pi were implemented to analyze selective sweep regions simultaneously with the window size set to 1 Mb, and only windows with an interquartile range for Tajima's D and diversity Pi of 1.5-fold in the whole genome were regarded as putative selection regions. The Gene Ontology (GO) terms were downloaded from the Ensembl website using the BioMart tool (http://asia.ensembl.org/biomart/martview/), and the Kyoto Encyclopedia of Genes and Genomes (KEGG) pathway was obtained according to the NCBI gene

accession number, and both GO and KEGG terms were organism specific (*S. scrofa*). Finally, annotations of 335,522 GO terms and 6,139 KEGG pathways were retained for enrichment analyses. Both enrichment analyses were performed using the OmicShare tools (http://www.omicshare.com/tools), and the significance was determined by the *P* value according to the hypergeometric test ($P < 0.05$).

Genome-wide association and Heritability estimation

A mixed linear model (MLM) approach was used for the genome-wide association analyses based on tagging SNPs, as implemented in the GCTA package [69]. The statistical model included the year and month as discrete covariates. For BF, LMA, LMD, and LMP, the year and season were included as discrete covariates, and the weights at the beginning and end of the test were used as quantitative covariates. To correct for multiple testing across the genome, the FDR correction obtained using FDRtool R package [72] was applied to determine the genome-wide significance threshold (FDR < 0.05). Once a QTL had been mapped using the tagging SNPs and exceeded the FDR threshold, association was re-calculated with all imputed SNPs (from the 11.3 M set) in a 20 Mb window around the peak using the same mixed model. The definition of a fine-mapped candidate QTL interval was based on the linkage disequilibrium (LD) level between the most significant SNP and all flanking sites, where the boundary was verified when the LD was no more than 0.8. The SNP effect was estimated using the GREML_CE program in the GVCBLUP package [73], where the result was absoluted and normalized.

Heritability was estimated using a mixed model as follows:

$$\mathbf{y} = \mathbf{X_b b} + \mathbf{Za} + \mathbf{e}$$

with Var($\mathbf{y}$) = $\mathbf{ZA_aZ'}\sigma_a^2 + \mathbf{I}\sigma_e^2$, where Z is an incidence matrix allocating phenotypic observations to each animal; $\mathbf{b}$ is the vector of the fixed year-month effects for BF, LMA, LMD, and LMP that also includes the weights at the beginning and end of the test as covariance; $\mathbf{X_b}$ is the incidence matrix for $\mathbf{b}$; $\mathbf{a}$ is the vector of additive values based on the genotype data; $\mathbf{A_a}$ is a genomic additive relationship matrix; σ_a^2 is the additive variance; and σ_e^2 is the residual variance. Variance components were estimated

by genomic restricted maximum likelihood estimation (GREML) using the GREML_CE program in the GVCBLUP package. The additive heritability was defined as: $h_a^2 = \sigma_a^2 / (\sigma_a^2 + \sigma_e^2)$. SNP effects were defined by the GREML_CE program and then normalized using R script.

The heritability of the detected QTL was estimated as follows:

$$\mathbf{y = X'_b b' + Za + e}$$

with Var(**y**) = $\mathbf{ZA_aZ'}\sigma_a^2 + \mathbf{I}\sigma_e^2$, where Z is an incidence matrix allocating phenotypic observations to each animal; **b'** is the vector of the fixed year-month effects and significant SNPs identified in the QTL region using GWAS analysis for BF, LMA, LMD and LMP; **b** also includes the weights at the beginning and end of the test as covariance; $\mathbf{X'_b}$ is the incidence matrix for **b**; **a** is the vector of additive values based on the genotype data; $\mathbf{A_a}$ is a genomic additive relationship matrix; σ_a^2 is the additive variance; and σ_e^2 is the residual variance. The QTL heritability was defined as $h_{qtl}^2 = h_a^2 - \sigma_a^2 / (\sigma_a^2 + \sigma_e^2)$.

Functional Consequence of the Missense Mutations associated with TN

The effect of the missense SNPs associated with TN on the stability of pig ABCD4, PROX2, and DLST proteins was assessed using I-Mutant adaptation 2.0 [74]. A potential surge or reduction in the DDG was predicted, along with a reliability index (RI), where the lowest and highest reliability levels were 0 and 10, respectively.

Direct genotyping by Fluidigm IFC technology

Sixteen loci on SSC7 were selected based on the GWAS results, three of which were related to BF, and the others were related to TN. Primers for genotyping were designed and ordered on the Fluidigm D3 assay design website (Supplementary Table S13), and 191 out of the total 2,869 pigs were genotyped for each SNP using Fluidigm Dynamic array IFC (Integrated Fluidic Circuit).

Data availability

All of the sequencing raw data in this study have been deposited into NCBI with accession number PRJNA681437 and PRJNA712489. Scripts, VCF files, phenotype information for seven traits, and other supporting data are available via the GigaScience repository, GigaDB [75]. The individual index information of the LCS dataset is listed in Supplementary Table S13.

List of Abbreviations

LCS: Low coverage sequencing method; GC: genotypic concordance; TTN: Total teat number; LTN: Left teat number; RTN: Right teat number; BF: Back fat thickness at 100 Kg; LMD: Loin muscle depth at 100 Kg; LMA: Loin muscle area at 100 Kg; LMP: Lean meat percentage at 100 Kg; TPD: Time spent to eat per day; ADFI: Average daily feed intake; NVD: Number of visits to feeder per day; TPV: Time spent to eat per visit; FR: Feed intake rate; FPV: Feed intake per visit; FCR: Feed conversion rate; ADG30: Average daily gain (0-30 Kg); AGE30: Age to 30 kg live weight; ADG100: Average daily gain (30-100 Kg); AGE100: Age to 100 kg live weight; BL: Body length; BH: Body height; CC: Circumference of cannon bone.

Competing interests

The authors declare that they have no competing interests.

Funding

This study is supported by the financial support of the National Transgenic Grand Project [2016ZX08009003-006], the 948 Program of the Ministry of Agriculture of China (2012-G1(4)), the Science and Technology Innovation Strategy Projects of Guangdong Province [2019B020203002], and the Guangdong Academician Workstation [2011A090700016].

Authors' contributions

XH, NL: conceptualization. XH, NL, YW, and ZW: project administration, and supervision. XH, YW, DZ, XG, JR, ZH, CB and RY: methodology, investigation, and formal analysis. RY, DZ, XG and YW: data curation and validation. ZW, GC, DL and CT: resources. XH and ZW: funding acquisition. YW and RY: visualization and original draft preparation. YW, XH, YZ, GC and DL: review and editing.

Acknowledgements

The authors thank Zhuo Song, Chungen Yi, and Wenjuan Wei for providing the services of FPGA-based hardware accelerator platform and the Batch Compute system in Aliyun cloud. The authors also thank Siyang Liu and Xun Xu for their valuable suggestions on data analyses, and Li Ma and Zhaoliang Liu for improving the manuscript. Part of the analysis was performed on the high-performance computing platform of the State Key Laboratory of Agrobiotechnology.

References

1. Visscher PM, Brown MA, McCarthy MI and Yang J. Five years of GWAS discovery. Am J Hum Genet. 2012;90 1:7-24. doi:10.1016/j.ajhg.2011.11.029.
2. Huang X, Wei X, Sang T, Zhao Q, Feng Q, Zhao Y, et al. Genome-wide association studies of 14 agronomic traits in rice landraces. Nat Genet. 2010;42 11:961-7. doi:10.1038/ng.695.
3. Marchini J and Howie B. Genotype imputation for genome-wide association studies. Nat Rev Genet. 2010;11 7:499-511. doi:10.1038/nrg2796.
4. Marchini J, Howie B, Myers S, McVean G and Donnelly P. A new multipoint method for genome-wide association studies by imputation of genotypes. Nat Genet. 2007;39 7:906-13. doi:10.1038/ng2088.
5. Howie BN, Donnelly P and Marchini J. A flexible and accurate genotype imputation method for the next generation of genome-wide association studies. PLoS Genet. 2009;5 6:e1000529. doi:10.1371/journal.pgen.1000529.
6. Howie B, Fuchsberger C, Stephens M, Marchini J and Abecasis GR. Fast and accurate genotype imputation in genome-wide association studies through pre-phasing. Nat Genet. 2012;44 8:955-+. doi:10.1038/ng.2354.
7. Yan G, Qiao R, Zhang F, Xin W, Xiao S, Huang T, et al. Imputation-Based Whole-Genome Sequence Association Study Rediscovered the Missing QTL for Lumbar Number in Sutai Pigs. Sci Rep. 2017;7 1:615. doi:10.1038/s41598-017-00729-0.
8. Van Binsbergen R, Bink MC, Calus MP, van Eeuwijk FA, Hayes BJ, Hulsegge I, et al. Accuracy of imputation to whole-genome sequence data in Holstein Friesian cattle. Genet Sel Evol. 2014;46:41. doi:10.1186/1297-9686-46-41.

9. Van den Berg S, Vandenplas J, van Eeuwijk FA, Bouwman AC, Lopes MS and Veerkamp RF. Imputation to whole-genome sequence using multiple pig populations and its use in genome-wide association studies. Genet Sel Evol. 2019;51 doi:10.1186/s12711-019-0445-y.
10. Swarts K, Li HH, Navarro JAR, An D, Romay MC, Hearne S, et al. Novel Methods to Optimize Genotypic Imputation for Low-Coverage, Next-Generation Sequence Data in Crop Plants. Plant Genome-Us. 2014;7 3 doi:10.3835/plantgenome2014.05.0023.
11. Buerkle CA and Gompert Z. Population genomics based on low coverage sequencing: how low should we go? Mol Ecol. 2013;22 11:3028-35. doi:10.1111/mec.12105.
12. Huang L, Wang B, Chen RT, Bercovici S and Batzoglou S. Reveel: large-scale population genotyping using low-coverage sequencing data. Bioinformatics. 2016;32 11:1686-96. doi:10.1093/bioinformatics/btv530.
13. Li Y, Sidore C, Kang HM, Boehnke M and Abecasis GR. Low-coverage sequencing: implications for design of complex trait association studies. Genome Res. 2011;21 6:940-51. doi:10.1101/gr.117259.110.
14. Gilly A, Southam L, Suveges D, Kuchenbaecker K, Moore R, Melloni GEM, et al. Very low-depth whole-genome sequencing in complex trait association studies. Bioinformatics. 2019;35 15:2555-61. doi:10.1093/bioinformatics/bty1032.
15. Davies RW, Flint J, Myers S and Mott R. Rapid genotype imputation from sequence without reference panels. Nat Genet. 2016;48 8:965-+. doi:10.1038/ng.3594.
16. Ros-Freixedes R, Gonen S, Gorjanc G and Hickey JM. A method for allocating low-coverage sequencing resources by targeting haplotypes rather than individuals. Genet Sel Evol. 2017;49 doi:10.1186/s12711-017-0353-y.
17. Fragoso CA, Heffelfinger C, Zhao HY and Dellaporta SL. Imputing Genotypes in Biallelic Populations from Low-Coverage Sequence Data. Genetics. 2016;202 2:487-+. doi:10.1534/genetics.115.182071.
18. Bickhart DM, Hutchison JL, Null DJ, VanRaden PM and Cole JB. Reducing animal sequencing redundancy by preferentially selecting animals with low-frequency haplotypes. J Dairy Sci. 2016;99 7:5526-34. doi:10.3168/jds.2015-10347.
19. Zan Y, Payen T, Lillie M, Honaker CF, Siegel PB and Carlborg O. Genotyping by low-coverage whole-genome sequencing in intercross pedigrees from outbred founders: a cost-efficient approach. Genet Sel Evol. 2019;51 1:44. doi:10.1186/s12711-019-0487-1.
20. Nicod J, Davies RW, Cai N, Hassett C, Goodstadt L, Cosgrove C, et al. Genome-wide association of multiple complex traits in outbred mice by ultra-low-coverage sequencing. Nat Genet. 2016;48 8:912-8. doi:10.1038/ng.3595.
21. Genomes Project C, Abecasis GR, Altshuler D, Auton A, Brooks LD, Durbin RM, et al. A map of human genome variation from population-scale sequencing. Nature. 2010;467 7319:1061-73. doi:10.1038/nature09534.
22. GenomeAsia KC. The GenomeAsia 100K Project enables genetic discoveries across Asia. Nature. 2019;576 7785:106-11. doi:10.1038/s41586-019-1793-z.
23. Wang Q, Pierce-Hoffman E, Cummings BB, Alfoldi J, Francioli LC, Gauthier LD, et al. Landscape of multi-nucleotide variants in 125,748 human exomes and 15,708 genomes. Nat Commun. 2020;11 1:2539. doi:10.1038/s41467-019-12438-5.
24. Genome of the Netherlands C. Whole-genome sequence variation, population structure and demographic history of the Dutch population. Nat Genet. 2014;46 8:818-25.

doi:10.1038/ng.3021.
25. Gudbjartsson DF, Sulem P, Helgason H, Gylfason A, Gudjonsson SA, Zink F, et al. Sequence variants from whole genome sequencing a large group of Icelanders. Sci Data. 2015;2:150011. doi:10.1038/sdata.2015.11.
26. Lam HM, Xu X, Liu X, Chen W, Yang G, Wong FL, et al. Resequencing of 31 wild and cultivated soybean genomes identifies patterns of genetic diversity and selection. Nat Genet. 2010;42 12:1053-9. doi:10.1038/ng.715.
27. Daetwyler HD, Capitan A, Pausch H, Stothard P, van Binsbergen R, Brondum RF, et al. Whole-genome sequencing of 234 bulls facilitates mapping of monogenic and complex traits in cattle. Nat Genet. 2014;46 8:858-65. doi:10.1038/ng.3034.
28. Hayes BJ and Daetwyler HD. 1000 Bull Genomes Project to Map Simple and Complex Genetic Traits in Cattle: Applications and Outcomes. Annu Rev Anim Biosci. 2019;7:89-102. doi:10.1146/annurev-animal-020518-115024.
29. Yang J, Benyamin B, McEvoy BP, Gordon S, Henders AK, Nyholt DR, et al. Common SNPs explain a large proportion of the heritability for human height. Nat Genet. 2010;42 7:565-9. doi:10.1038/ng.608.
30. Lango Allen H, Estrada K, Lettre G, Berndt SI, Weedon MN, Rivadeneira F, et al. Hundreds of variants clustered in genomic loci and biological pathways affect human height. Nature. 2010;467 7317:832-8. doi:10.1038/nature09410.
31. Tan C, Wu ZF, Ren JL, Huang ZL, Liu DW, He XY, et al. Genome-wide association study and accuracy of genomic prediction for teat number in Duroc pigs using genotyping-by-sequencing. Genet Sel Evol. 2017;49 doi:10.1186/s12711-017-0311-8.
32. Ros-Freixedes R, Battagin M, Johnsson M, Gorjanc G, Mileham AJ, Rounsley SD, et al. Impact of index hopping and bias towards the reference allele on accuracy of genotype calls from low-coverage sequencing. Genet Sel Evol. 2018;50 1:64. doi:10.1186/s12711-018-0436-4.
33. Liu S, Huang S, Chen F, Zhao L, Yuan Y, Francis SS, et al. Genomic Analyses from Non-invasive Prenatal Testing Reveal Genetic Associations, Patterns of Viral Infections, and Chinese Population History. Cell. 2018;175 2:347-59 e14. doi:10.1016/j.cell.2018.08.016.
34. Browning BL and Browning SR. A unified approach to genotype imputation and haplotype-phase inference for large data sets of trios and unrelated individuals. Am J Hum Genet. 2009;84 2:210-23. doi:10.1016/j.ajhg.2009.01.005.
35. Paudel Y, Madsen O, Megens HJ, Frantz LA, Bosse M, Crooijmans RP, et al. Copy number variation in the speciation of pigs: a possible prominent role for olfactory receptors. BMC Genomics. 2015;16:330. doi:10.1186/s12864-015-1449-9.
36. Zhuang Z, Ding R, Peng L, Wu J, Ye Y, Zhou S, et al. Genome-wide association analyses identify known and novel loci for teat number in Duroc pigs using single-locus and multi-locus models. BMC Genomics. 2020;21 1:344. doi:10.1186/s12864-020-6742-6.
37. Van Son M, Lopes MS, Martell HJ, Derks MFL, Gangsei LE, Kongsro J, et al. A QTL for Number of Teats Shows Breed Specific Effects on Number of Vertebrae in Pigs: Bridging the Gap Between Molecular and Quantitative Genetics. Front Genet. 2019;10:272. doi:10.3389/fgene.2019.00272.
38. Moscatelli G, Dall'Olio S, Bovo S, Schiavo G, Kazemi H, Ribani A, et al. Genome-wide association studies for the number of teats and teat asymmetry patterns in Large White pigs. Anim Genet. 2020;51 4:595-600. doi:10.1111/age.12947.

39. Pistocchi A, Bartesaghi S, Cotelli F and Del Giacco L. Identification and expression pattern of zebrafish prox2 during embryonic development. Dev Dyn. 2008;237 12:3916-20. doi:10.1002/dvdy.21798.
40. Ren DR, Ren J, Ruan GF, Guo YM, Wu LH, Yang GC, et al. Mapping and fine mapping of quantitative trait loci for the number of vertebrae in a White Duroc x Chinese Erhualian intercross resource population. Anim Genet. 2012;43 5:545-51. doi:10.1111/j.1365-2052.2011.02313.x.
41. Gong H, Xiao S, Li W, Huang T, Huang X, Yan G, et al. Unravelling the genetic loci for growth and carcass traits in Chinese Bamaxiang pigs based on a 1.4 million SNP array. J Anim Breed Genet. 2019;136 1:3-14. doi:10.1111/jbg.12365.
42. Liu X, Wang LG, Liang J, Yan H, Zhao KB, Li N, et al. Genome-Wide Association Study for Certain Carcass Traits and Organ Weights in a Large WhitexMinzhu Intercross Porcine Population. J Integr Agr. 2014;13 12:2721-30. doi:10.1016/S2095-3119(14)60787-5.
43. Arce-Cerezo A, Garcia M, Rodriguez-Nuevo A, Crosa-Bonell M, Enguix N, Pero A, et al. HMGA1 overexpression in adipose tissue impairs adipogenesis and prevents diet-induced obesity and insulin resistance. Sci Rep. 2015;5:14487. doi:10.1038/srep14487.
44. Wang LG, Zhang LC, Yan H, Liu X, Li N, Liang J, et al. Genome-Wide Association Studies Identify the Loci for 5 Exterior Traits in a Large White x Minzhu Pig Population. Plos One. 2014;9 8 doi:10.1371/journal.pone.0103766.
45. Ji JX, Yan GR, Chen D, Xiao SJ, Gao J and Zhang ZY. An association study using imputed whole-genome sequence data identifies novel significant loci for growth-related traits in a Duroc x Erhualian F-2 population. J Anim Breed Genet. 2019;136 3:217-28. doi:10.1111/jbg.12389.
46. Tang Z, Xu J, Yin L, Yin D, Zhu M, Yu M, et al. Genome-Wide Association Study Reveals Candidate Genes for Growth Relevant Traits in Pigs. Front Genet. 2019;10:302. doi:10.3389/fgene.2019.00302.
47. Hoque MA, Kadowaki H, Shibata T, Oikawa T and Suzuki K. Genetic parameters for measures of residual feed intake and growth traits in seven generations of Duroc pigs. Livestock Science. 2009;121 1:45-9. doi:https://doi.org/10.1016/j.livsci.2008.05.016.
48. Fontanesi L, Schiavo G, Galimberti G, Calo DG and Russo V. A genomewide association study for average daily gain in Italian Large White pigs. J Anim Sci. 2014;92 4:1385-94. doi:10.2527/jas.2013-7059.
49. Silva EF, Lopes MS, Lopes PS and Gasparino E. A genome-wide association study for feed efficiency-related traits in a crossbred pig population. Animal. 2019;13 11:2447-56. doi:10.1017/S1751731119000910.
50. Qiao R, Gao J, Zhang Z, Li L, Xie X, Fan Y, et al. Genome-wide association analyses reveal significant loci and strong candidate genes for growth and fatness traits in two pig populations. Genet Sel Evol. 2015;47:17. doi:10.1186/s12711-015-0089-5.
51. Ding R, Yang M, Wang X, Quan J, Zhuang Z, Zhou S, et al. Genetic Architecture of Feeding Behavior and Feed Efficiency in a Duroc Pig Population. Front Genet. 2018;9:220. doi:10.3389/fgene.2018.00220.
52. Meuwissen T and Goddard M. Accurate prediction of genetic values for complex traits by whole-genome resequencing. Genetics. 2010;185 2:623-31. doi:10.1534/genetics.110.116590.
53. Zhang C, Kemp RA, Stothard P, Wang Z, Boddicker N, Krivushin K, et al. Genomic evaluation

of feed efficiency component traits in Duroc pigs using 80K, 650K and whole-genome sequence variants. Genet Sel Evol. 2018;50 1:14. doi:10.1186/s12711-018-0387-9.

54. Yan G, Guo T, Xiao S, Zhang F, Xin W, Huang T, et al. Imputation-Based Whole-Genome Sequence Association Study Reveals Constant and Novel Loci for Hematological Traits in a Large-Scale Swine F2 Resource Population. Front Genet. 2018;9:401. doi:10.3389/fgene.2018.00401.

55. Edwards SM, Sorensen IF, Sarup P, Mackay TF and Sorensen P. Genomic Prediction for Quantitative Traits Is Improved by Mapping Variants to Gene Ontology Categories in Drosophila melanogaster. Genetics. 2016;203 4:1871-83. doi:10.1534/genetics.116.187161.

56. Xiang R, Berg IVD, MacLeod IM, Hayes BJ, Prowse-Wilkins CP, Wang M, et al. Quantifying the contribution of sequence variants with regulatory and evolutionary significance to 34 bovine complex traits. Proc Natl Acad Sci U S A. 2019;116 39:19398-408. doi:10.1073/pnas.1904159116.

57. Xing Y, Li G, Wang Z, Feng B, Song Z and Wu C. GTZ: a fast compression and cloud transmission tool optimized for FASTQ files. BMC Bioinformatics. 2017;18 Suppl 16:549. doi:10.1186/s12859-017-1973-5.

58. Bosse M, Megens HJ, Frantz LA, Madsen O, Larson G, Paudel Y, et al. Genomic analysis reveals selection for Asian genes in European pigs following human-mediated introgression. Nat Commun. 2014;5:4392. doi:10.1038/ncomms5392.

59. Bosse M, Lopes MS, Madsen O, Megens HJ, Crooijmans RP, Frantz LA, et al. Artificial selection on introduced Asian haplotypes shaped the genetic architecture in European commercial pigs. Proc Biol Sci. 2015;282 1821:20152019. doi:10.1098/rspb.2015.2019.

60. Groenen MA, Archibald AL, Uenishi H, Tuggle CK, Takeuchi Y, Rothschild MF, et al. Analyses of pig genomes provide insight into porcine demography and evolution. Nature. 2012;491 7424:393-8. doi:10.1038/nature11622.

61. Hoover KC. Smell with inspiration: the evolutionary significance of olfaction. Am J Phys Anthropol. 2010;143 Suppl 51:63-74. doi:10.1002/ajpa.21441.

62. Carneiro M, Rubin CJ, Di Palma F, Albert FW, Alfoldi J, Martinez Barrio A, et al. Rabbit genome analysis reveals a polygenic basis for phenotypic change during domestication. Science. 2014;345 6200:1074-9. doi:10.1126/science.1253714.

63. Boyle EA, Li YI and Pritchard JK. An Expanded View of Complex Traits: From Polygenic to Omnigenic. Cell. 2017;169 7:1177-86. doi:10.1016/j.cell.2017.05.038.

64. Wang Y, Cao X, Luo C, Sheng Z, Zhang C, Bian C, et al. Multiple ancestral haplotypes harboring regulatory mutations cumulatively contribute to a QTL affecting chicken growth traits. Commun Biol. 2020;3 1:472. doi:10.1038/s42003-020-01199-3.

65. Chakravarti A and Turner TN. Revealing rate-limiting steps in complex disease biology: The crucial importance of studying rare, extreme-phenotype families. Bioessays. 2016;38 6:578-86. doi:10.1002/bies.201500203.

66. Picelli S, Bjorklund AK, Reinius B, Sagasser S, Winberg G and Sandberg R. Tn5 transposase and tagmentation procedures for massively scaled sequencing projects. Genome Res. 2014;24 12:2033-40. doi:10.1101/gr.177881.114.

67. McKenna A, Hanna M, Banks E, Sivachenko A, Cibulskis K, Kernytsky A, et al. The Genome Analysis Toolkit: a MapReduce framework for analyzing next-generation DNA sequencing data. Genome Res. 2010;20 9:1297-303. doi:10.1101/gr.107524.110.

68. Cingolani P, Platts A, Wang le L, Coon M, Nguyen T, Wang L, et al. A program for annotating and predicting the effects of single nucleotide polymorphisms, SnpEff: SNPs in the genome of Drosophila melanogaster strain w1118; iso-2; iso-3. Fly (Austin). 2012;6 2:80-92. doi:10.4161/fly.19695.
69. Yang J, Lee SH, Goddard ME and Visscher PM. GCTA: a tool for genome-wide complex trait analysis. Am J Hum Genet. 2011;88 1:76-82. doi:10.1016/j.ajhg.2010.11.011.
70. Danecek P, Auton A, Abecasis G, Albers CA, Banks E, DePristo MA, et al. The variant call format and VCFtools. Bioinformatics. 2011;27 15:2156-8. doi:10.1093/bioinformatics/btr330.
71. Tajima F. Statistical method for testing the neutral mutation hypothesis by DNA polymorphism. Genetics. 1989;123 3:585-95.
72. Strimmer K. fdrtool: a versatile R package for estimating local and tail area-based false discovery rates. Bioinformatics. 2008;24 12:1461-2. doi:10.1093/bioinformatics/btn209.
73. Wang C, Prakapenka D, Wang S, Pulugurta S, Runesha HB and Da Y. GVCBLUP: a computer package for genomic prediction and variance component estimation of additive and dominance effects. BMC Bioinformatics. 2014;15:270. doi:10.1186/1471-2105-15-270.
74. Capriotti E, Calabrese R and Casadio R. Predicting the insurgence of human genetic diseases associated to single point protein mutations with support vector machines and evolutionary information. Bioinformatics. 2006;22 22:2729-34. doi:10.1093/bioinformatics/btl423.
75. Yang R, Guo X, Zhu D, Tan C, Bian C, Ren J, et al. Supporting data for "Accelerated Deciphering of the Genetic Architecture of Agricultural Economic Traits in Pigs Using the Low Coverage Whole-genome Sequencing Strategy". GigaScience Database. 2021; doi: . http://dx.doi.org/10.5524/100894

Figure legends

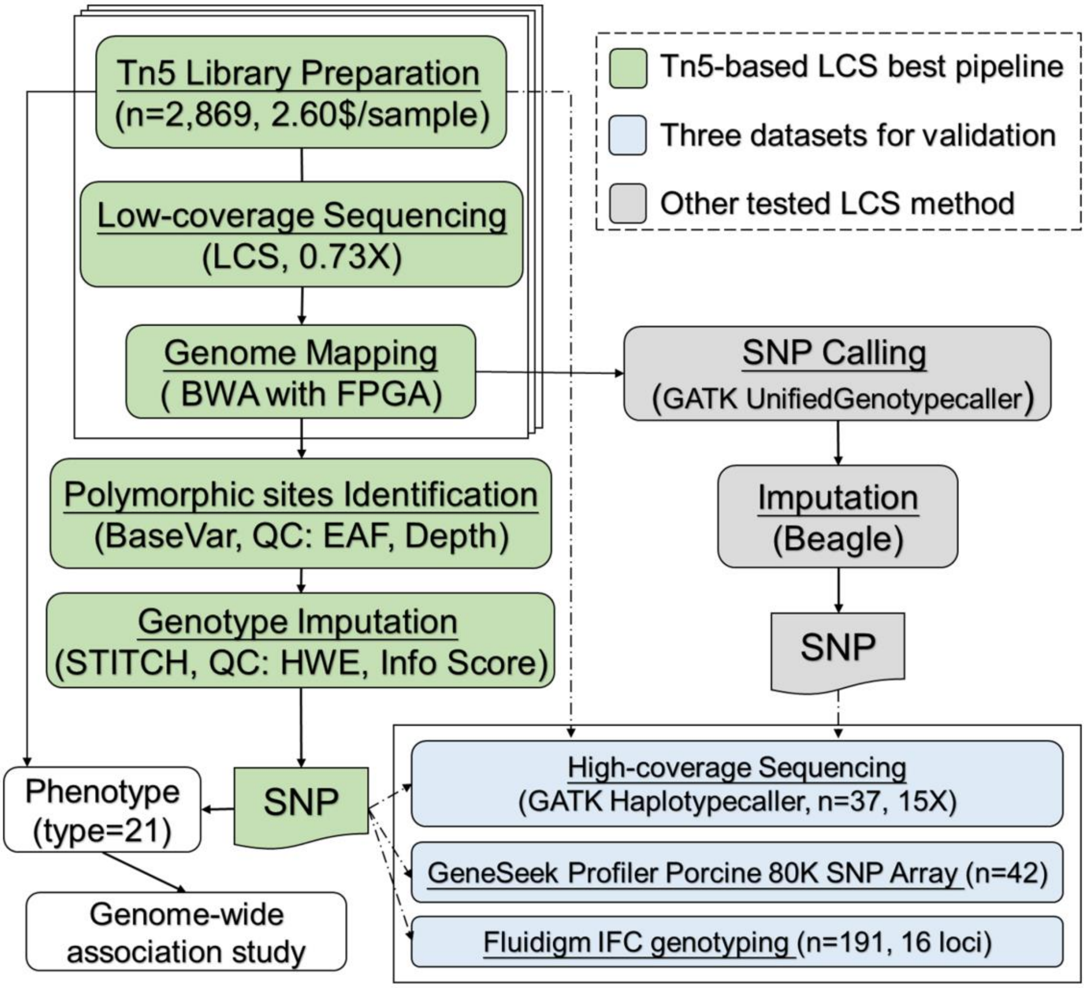

Figure 1 Low coverage sequencing (LCS) study design

The flow chart summarizes the steps used to identify and impute polymorphic sites, where the green block represents the highly accurate pipeline used for Tn5-based LCS analysis (BaseVar-STITCH). We also generated SNP results using the GATK-Beagle pipeline (grey) and compared them with those obtained using the BaseVar-STITCH method. Three datasets (blue) were used to assess the accuracy of the results. The BaseVar-STITCH pipeline was used in the GWAS presented in this study.

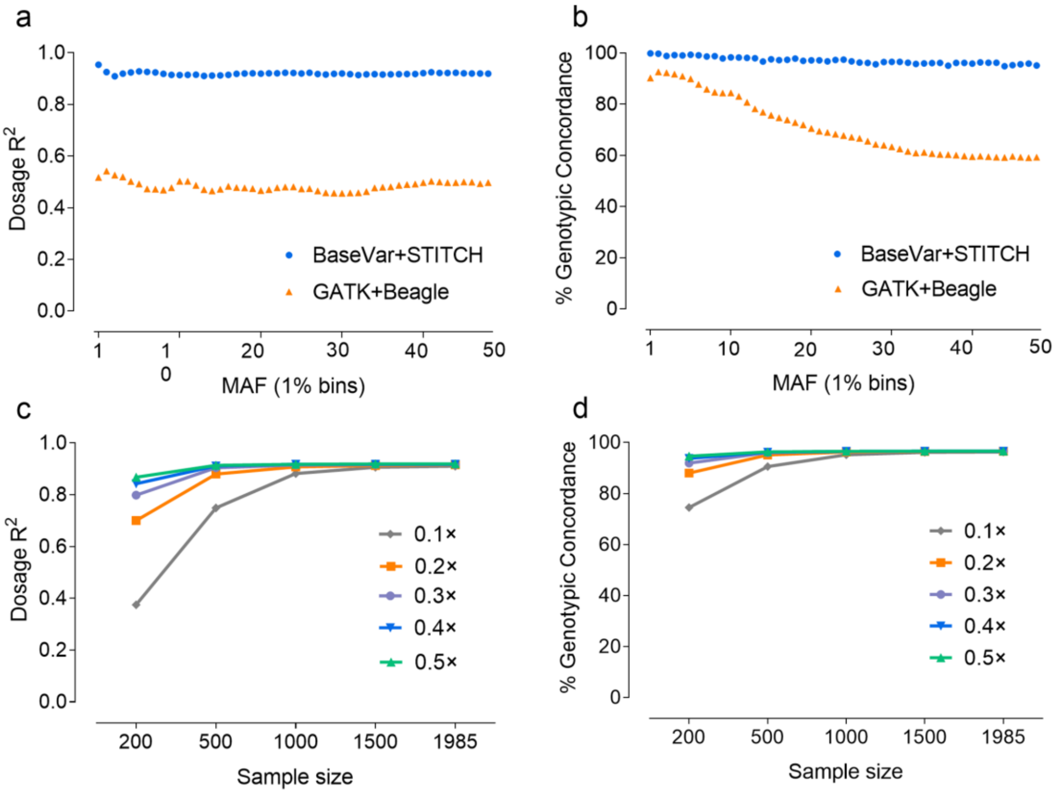

Figure 2 Performance of BaseVar-STITCH on different minor allele frequencies (MAFs) and sample sizes

The validation dataset is the high-coverage sequencing results of 37 individuals genotyped by GATK best practices (HaplotypeCaller model). **(a)** and **(b)** show a comparison of the dosage R^2 and genotypic concordance values (%) between the BaseVar-STITCH for low-coverage sequencing (LCS) (blue) and the GATK-Beagle (orange) pipelines, and **(c)** and **(d)** show the comparison of the dosage R^2 and genotypic concordance values (%) among different sequencing depths.

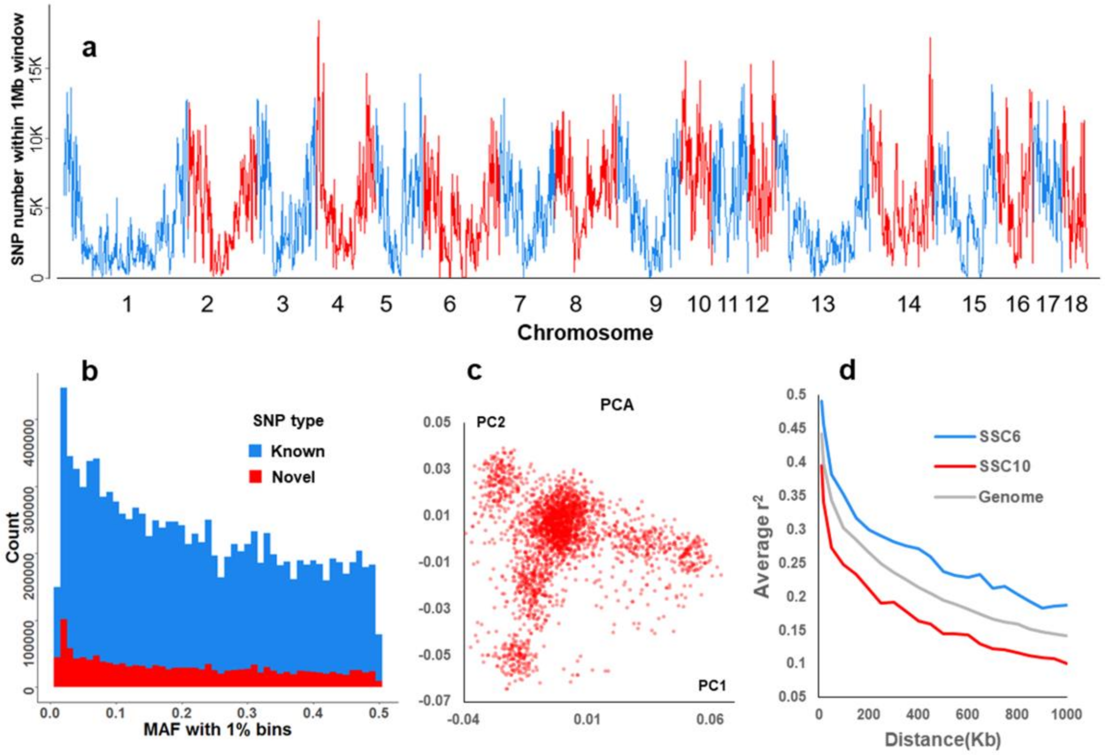

Figure 3 Genetic diversity of the Duroc population

(a) The distribution of SNPs in 1 Mb windows across the genome. **(b)** Histogram of allele counts by each 1% MAF bin. Novel (red) and known SNP sets (blue) were defined by comparing them to the pig dbSNP database. **(c)** Principal component 1 and 2 distribution in the Duroc population. **(d)** The extent of linkage disequilibrium (LD), in which the LD on chromosomes 6 (SSC6) and 10 (SSC10) represent the highest and lowest levels across the whole genome, respectively.

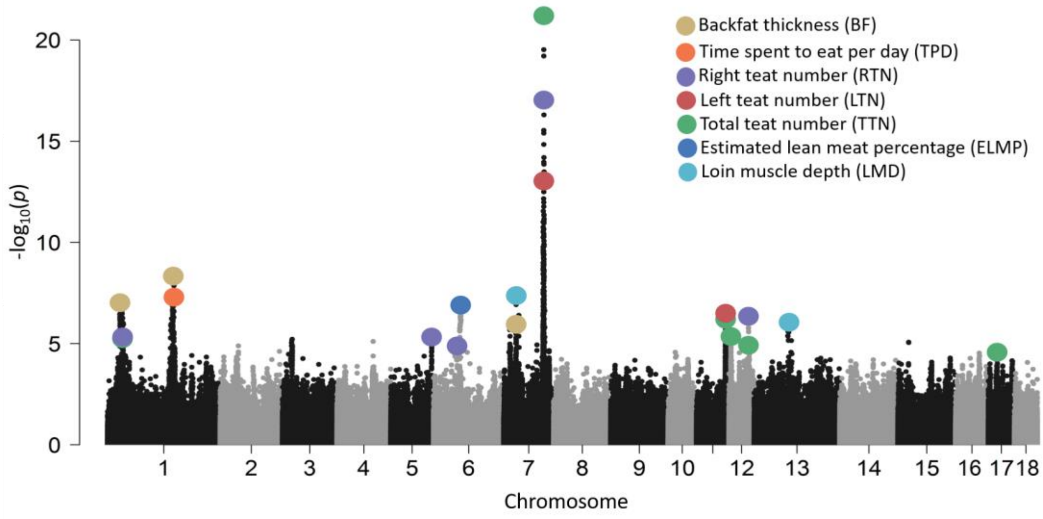

Figure 4 Summary Manhattan plot of seven phenotypes with significant SNPs

Genome-wide representation of all quantitative trait loci (QTLs) identified in this study. Light and dark grey dots show associations from the seven measures where at least one QTL was detected at the tagging SNP positions (n = 258,662). The most significant SNP positions at each QTL are marked with a colour dot.

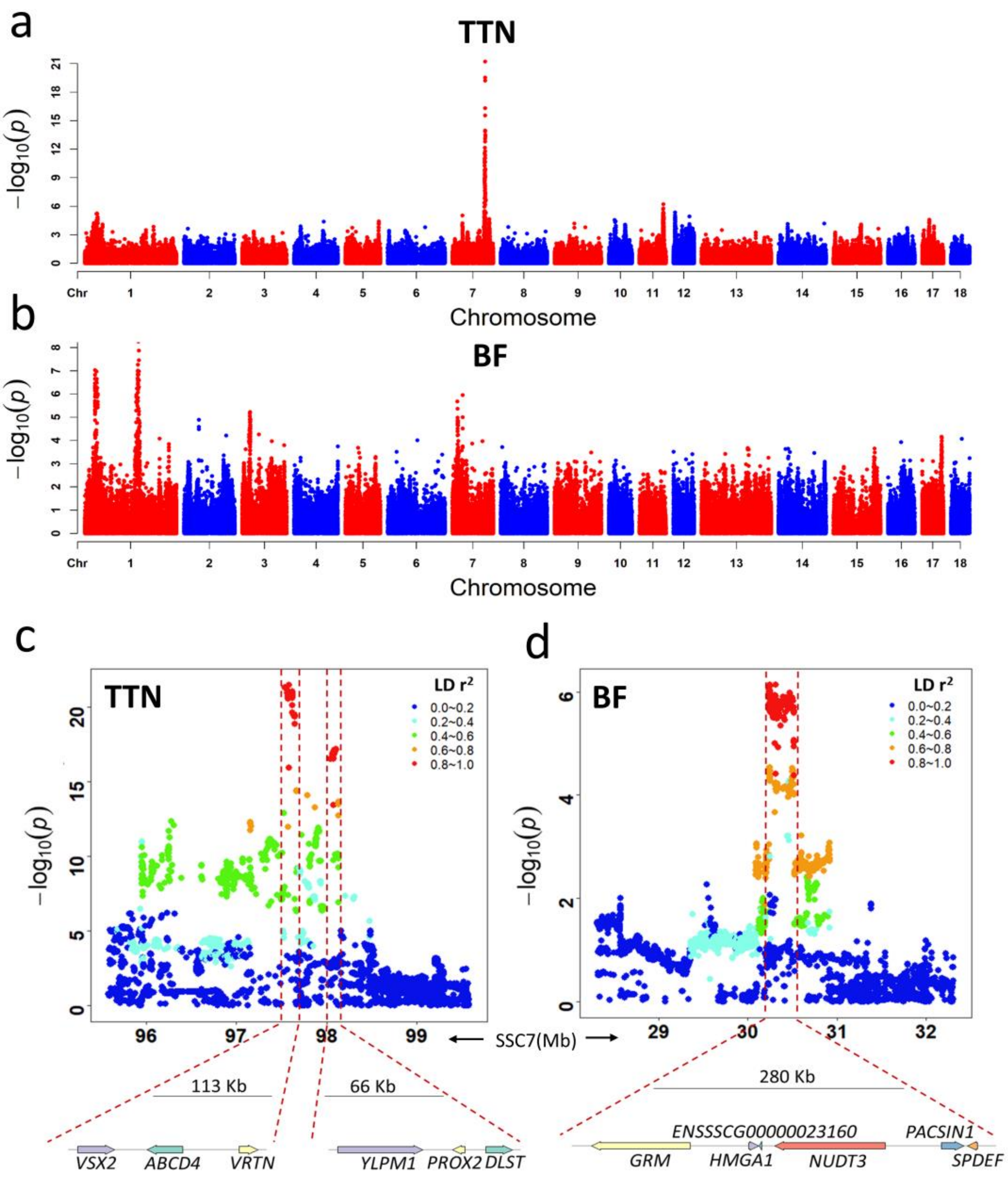

Figure 5 Manhattan plots and fine-mapping of the total teat number (TTN) and back fat thickness (BF)

(a) and (**b)** Depict the TTN and BF association signals on the whole genome. **(c)** Fine-mapping of the TTN using the entire set of SNPs, in which two isolated regions on chromosome 7 with lengths of 113 and 66 Kb were detected as QTLs. **(d)** Fine-mapping of BF using the entire set of SNPs. A narrow QTL with a length of 280 Kb was detected on chromosome 7. The association genes within QTLs are displayed below.

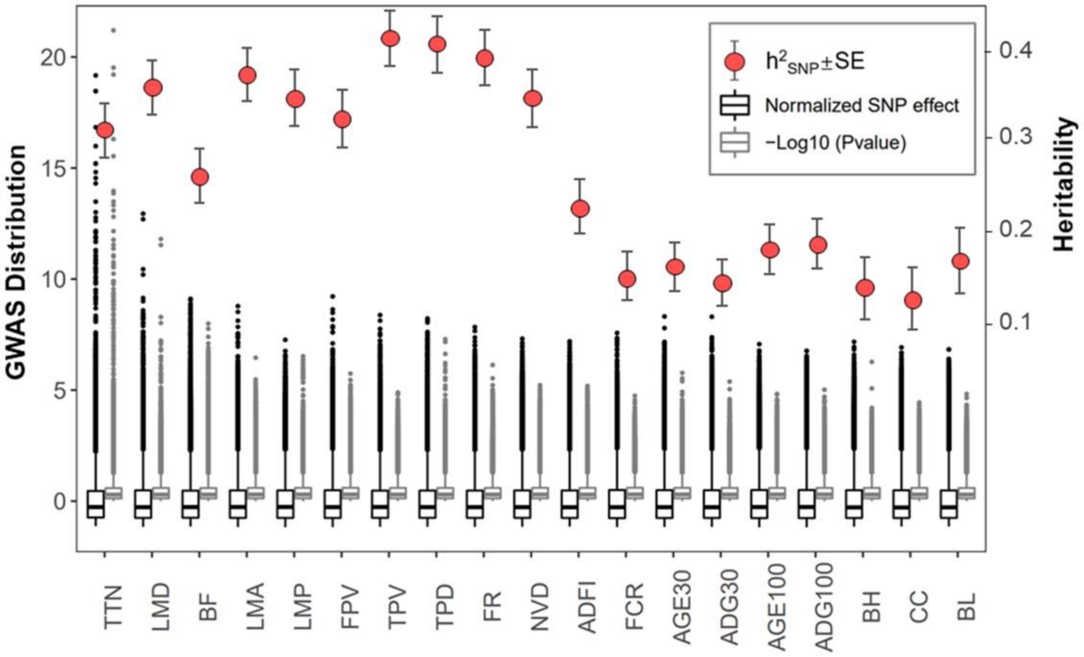

Figure 6 Heritability and SNP significance and normalized effect of 21 traits

The SNP effect was estimated and normalized and is displayed in the black boxplot. The grey boxplot represents the distribution of -$\log_{10} P$ values for all SNPs. Red dots represrent heritability estimates, while black lines represent standard deviations.

Table 1. QTLs mapping and contribution to heritability

Phenotype	Number	Mean ± standard deviation	Significant threshold[a]	QTL number	Variance explained(%)[b]	Gene number[c]
Total teat number (TTN)	2797	10.73 ± 1.07	4.55	6	8.86	52
Left teat number (LTN)	2797	5.35 ± 0.66	4.81	2	3.16	14
Right teat number (RTN)	2797	5.38 ± 0.64	4.79	5	6.03	56
Back fat thickness at 100 Kg (BF, mm)	2796	10.99 ± 2.66	4.67	4	2.40	55
Loin muscle depth at 100 Kg (LMD, mm)	2796	46.15 ± 3.93	5.36	2	1.27	15
Loin muscle area at 100 Kg (LMA, mm^2)	2795	36.25 ± 3.60	-	0	0	0
Lean meat percentage at 100 Kg (LMP, %)	2795	54.02 ± 1.58	5.50	1	1.19	48
Time spent to eat per day (TPD, min)	2602	63.02 ± 9.85	6.10	1	1.08	28
Average daily feed intake (ADFI, Kg)	2602	2.00 ± 0.20	-	0	0	0
Number of visits to feeder per day (NVD)	2602	7.30 ± 1.83	-	0	0	0
Time spent to eat per visit (TPV, min)	2602	10.06 ± 2.79	-	0	0	0
Feed intake rate (FR, g/min)	2602	32.37 ± 5.19	-	0	0	0
Feed intake per visit (FPV, Kg)	2602	290.6 ± 75.87	-	0	0	0
Feed conversion rate (FCR)	2691	2.19 ± 0.19	-	0	0	0
Average daily gain (0-30 Kg) (ADG30, g)	2795	354.8 ± 38.72	-	0	0	0
Age to 30 kg live weight (AGE30, day)	2796	80.49 ± 8.57	-	0	0	0
Average daily gain (30-100 Kg) (ADG100, g)	2795	633.8 ± 37.12	-	0	0	0
Age to 100 kg live weight (AGE100, day)	2796	155.5 ± 9.20	-	0	0	0
Body length (BL, cm)	1844	117.60 ± 2.91	-	0	0	0
Body height (BH, cm)	1844	62.19 ± 1.55	-	0	0	0
Circumference of cannon bone (CC, cm)	1844	17.81 ± 0.54	-	0	0	0

a. $-\text{Log}_{10}(p)$ value when FDR < 0.05; b. total phenotypic variance explained by QTLs; c. Total gene number included in QTLs.

Additional Files

Supplementary Figure 1 Phenotypic distribution of 21 traits

Supplementary Figure 2 Dosage R^2 and cost time (minute) among different K values

Accuracy and cost time of genotyping from K = 5 to K = 25, where the blue and black lines represent the dosage R^2 and cost time (minute) respectively.

Supplementary Figure 3 Purifying selection regions in the whole genome

Purifying selection signals were detected on SSC2, SSC3, SSC6, SSC7, SSC9 and SSC15, where blue and red lines represent $-\text{Log}_{10}$ Pi and Tajima's D respectively, and the grey regions depict the purifying selected regions.

Supplementary Figure 4 Manhattan plots of phenotypes with no significant SNPs

Manhattan plots of ADFT, NVD, TPV, FPV, FR, FCR, BH, BL, CC, ADG100, AGE100, ADG30, AGE30 and LMA, where no significant SNPs were detected in these traits.

Supplementary Figure 5 QQ plot of 21 phenotypes

Supplementary Figure 6 Summary plots of fine mapping

Supplementary Figure 7 Distribution of top 100 SNPs based on *P* value using GWAS analysis

Supplementary Figure 8 GO and KEGG enrichment of genes identified to be associated with feeding behavior traits

Supplementary Figure 9 Neurotrophin signaling pathway enrichment

The red tangles represent detected pathways in this study, which including Bcl-2, NT3, TrkB and p75NTF.

Supplementary Table S1 LCS data set

Supplementary Table S2 Resequencing Duroc samples list

Supplementary Table S3 Genotypic concordance between BaseVar-STITCH method and direct genotyping by Fluidigm IFC technology

Supplementary Table S4 Number and density of SNPs imputed by STITCH and Tag SNP

Supplementary Table S5 GO enrichment of genes located in the selected regions

Supplementary Table S6 Genetic and phenotypic coefficient of 21 traits

Supplementary Table S7 Summary of detected QTLs

Supplementary Table S8 Summary table of markers identified significantly associated with ADG, AGE or FCR in previous studies

Supplementary Table S9 Missense SNPs in the narrowed QTL region of TN

Supplementary Table S10 Gathered information of candidate genes

Supplementary Table S11 GO enrichment of genes located in the selected regions

Supplementary Table S12 KEGG enrichment of genes located in the selected regions

Supplementary Table S13 Index sequence for the all LCS samples

Supplementary Table S14 Primers used for Fluidigm IFC genotyping

 Click here to access/download;Table;Table 1.docx

Table 1 QTLs mapping and contribution to heritability

Phenotype	Number	Mean ± standard deviation	Significant threshold[a]	QTL number	Variance explained(%)[b]	Gene number[c]
Total teat number (TTN)	2797	10.73 ± 1.07	4.55	6	8.86	52
Left teat number (LTN)	2797	5.35 ± 0.66	4.81	2	3.16	14
Right teat number (RTN)	2797	5.38 ± 0.64	4.79	5	6.03	56
Back fat thickness at 100 Kg (BF, mm)	2796	10.99 ± 2.66	4.67	4	2.40	55
Loin muscle depth at 100 Kg (LMD, mm)	2796	46.15 ± 3.93	5.36	2	1.27	15
Loin muscle area at 100 Kg (LMA, mm^2)	2795	36.25 ± 3.60	-	0	0	0
Lean meat percentage at 100 Kg (LMP, %)	2795	54.02 ± 1.58	5.50	1	1.19	48
Time spent to eat per day (TPD, min)	2602	63.02 ± 9.85	6.10	1	1.08	28
Average daily feed intake (ADFI, Kg)	2602	2.00 ± 0.20	-	0	0	0
Number of visits to feeder per day (NVD)	2602	7.30 ± 1.83	-	0	0	0
Time spent to eat per visit (TPV, min)	2602	10.06 ± 2.79	-	0	0	0
Feed intake rate (FR, g/min)	2602	32.37 ± 5.19	-	0	0	0
Feed intake per visit (FPV, Kg)	2602	290.6 ± 75.87	-	0	0	0
Feed conversion rate (FCR)	2691	2.19 ± 0.19	-	0	0	0
Average daily gain (0-30 Kg) (ADG30, g)	2795	354.8 ± 38.72	-	0	0	0
Age to 30 kg live weight (AGE30, day)	2796	80.49 ± 8.57	-	0	0	0
Average daily gain (30-100 Kg) (ADG100, g)	2795	633.8 ± 37.12	-	0	0	0
Age to 100 kg live weight (AGE100, day)	2796	155.5 ± 9.20	-	0	0	0
Body length (BL, cm)	1844	117.60 ± 2.91	-	0	0	0
Body height (BH, cm)	1844	62.19 ± 1.55	-	0	0	0
Circumference of cannon bone (CC, cm)	1844	17.81 ± 0.54	-	0	0	0

Note: a. $-\text{Log}_{10}$ *P* value when FDR < 0.05; b. total phenotypic variance explained by QTLs; c. Total gene number included in QTLs.

Figure 1

Click here to access/download;Figure;Figure 1.tif

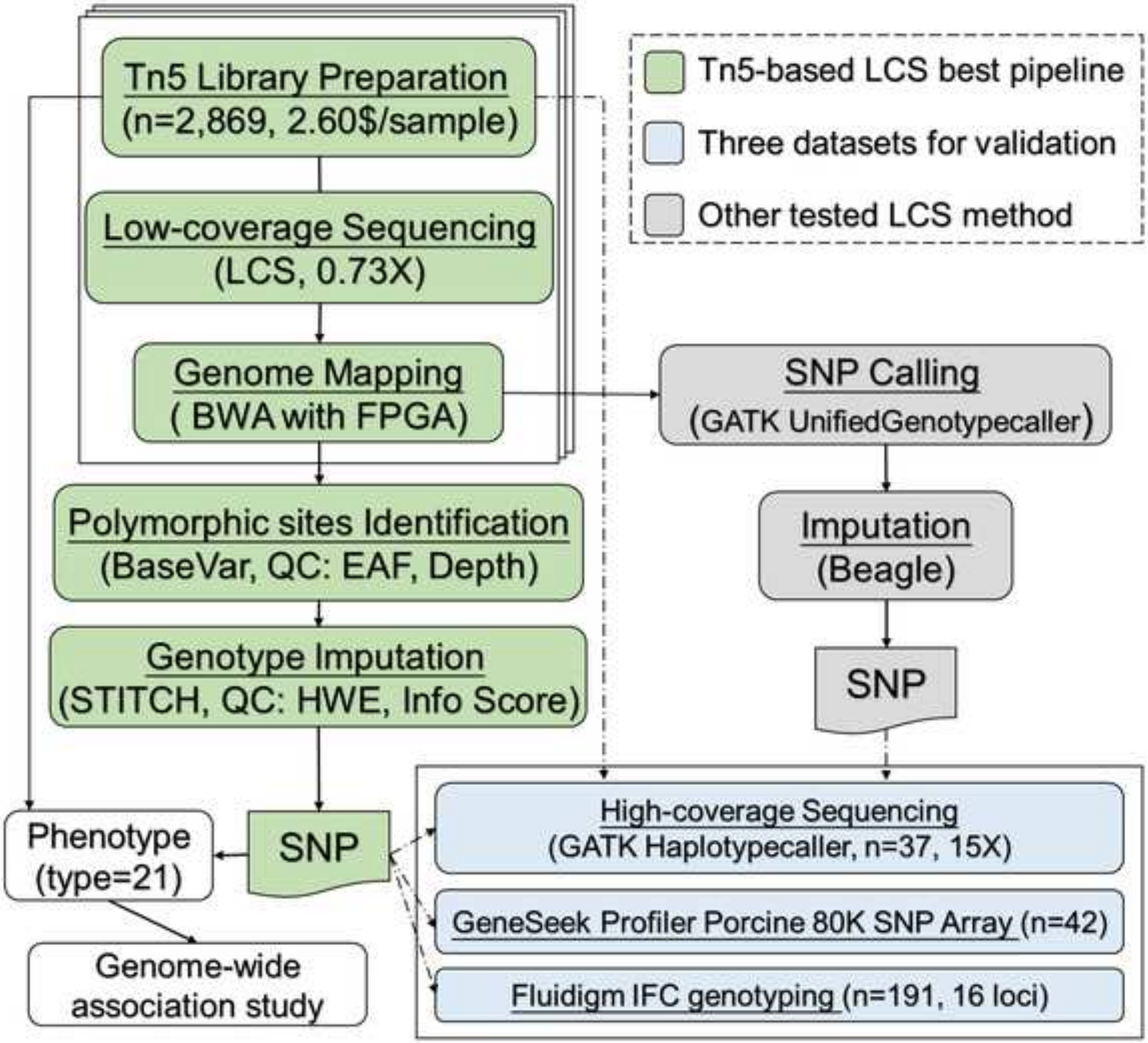

Figure 2

Click here to access/download;Figure;Figure 2.tif

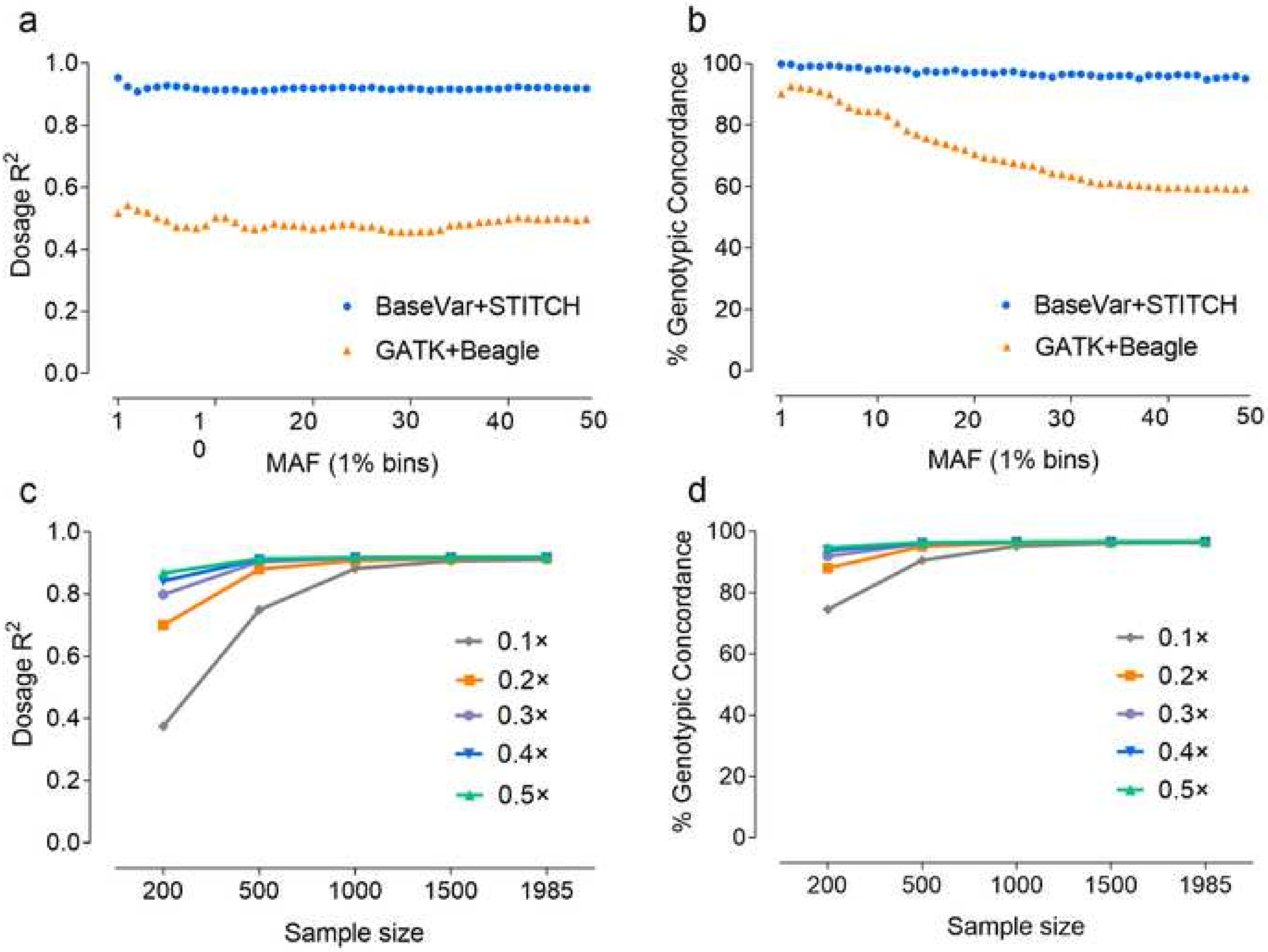

Figure 3

Click here to access/download;Figure;Figure 3.tif

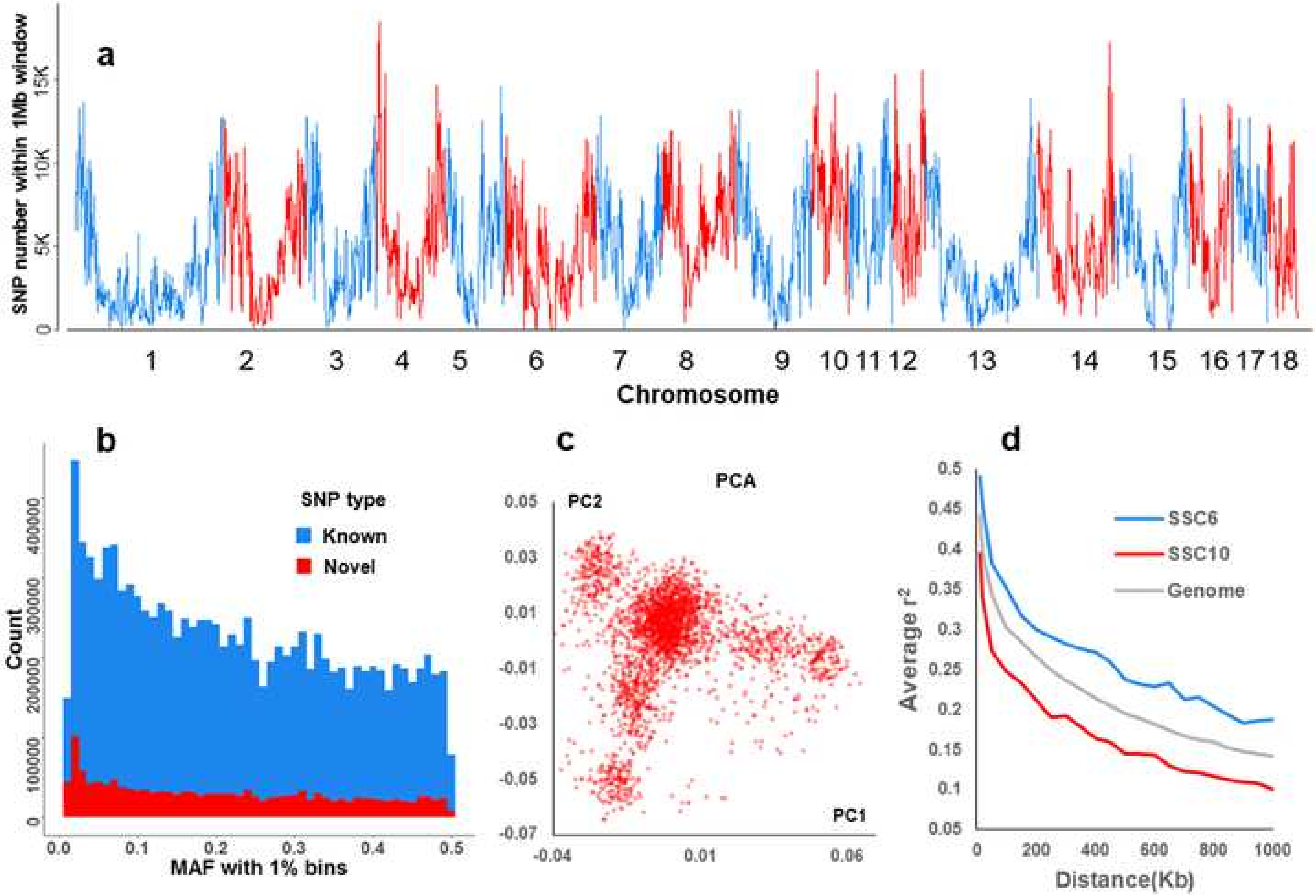

Figure 4

Click here to access/download;Figure;Figure 4.tif

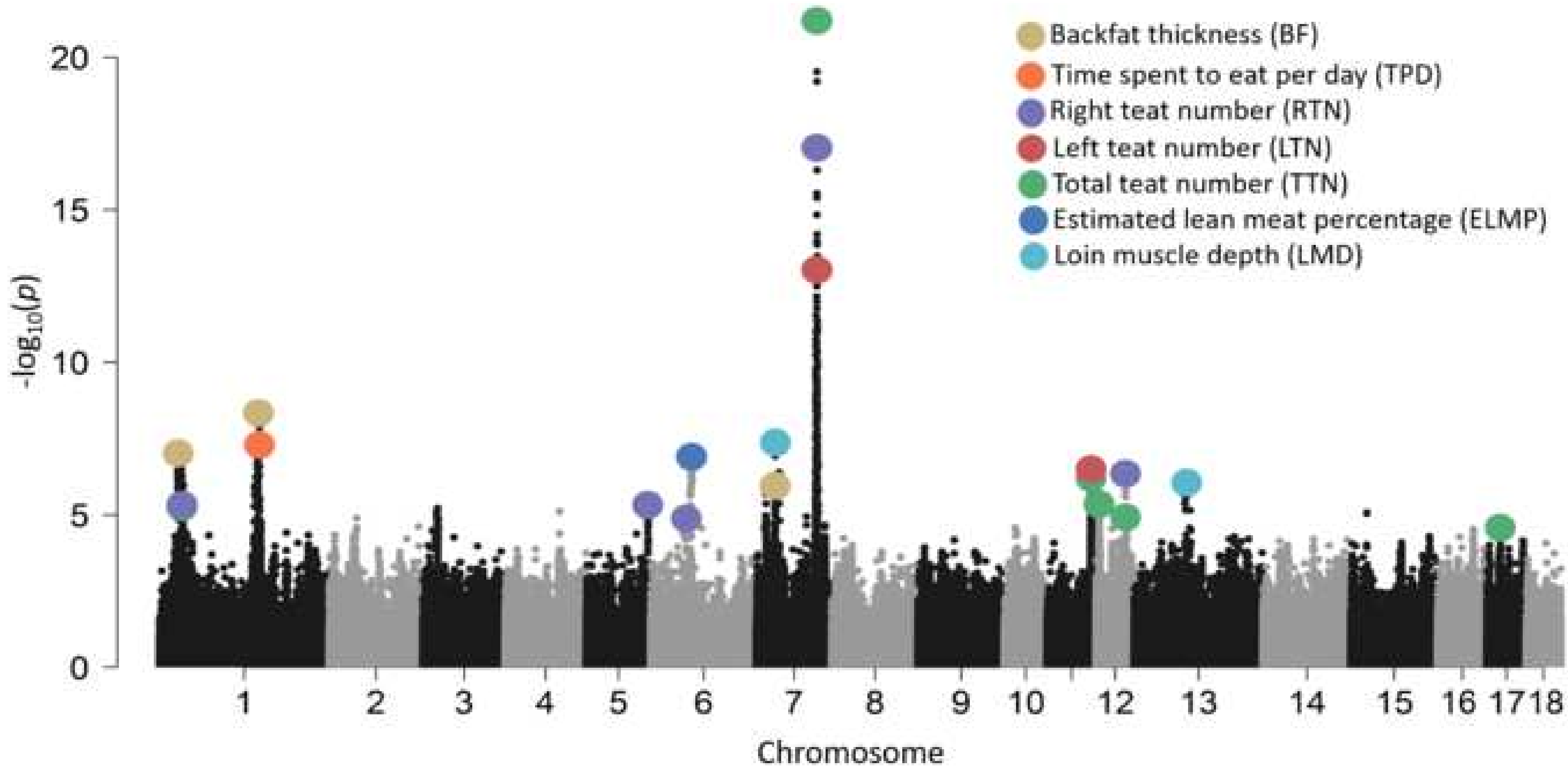

Figure 5

Click here to access/download;Figure;Figure 5.tif

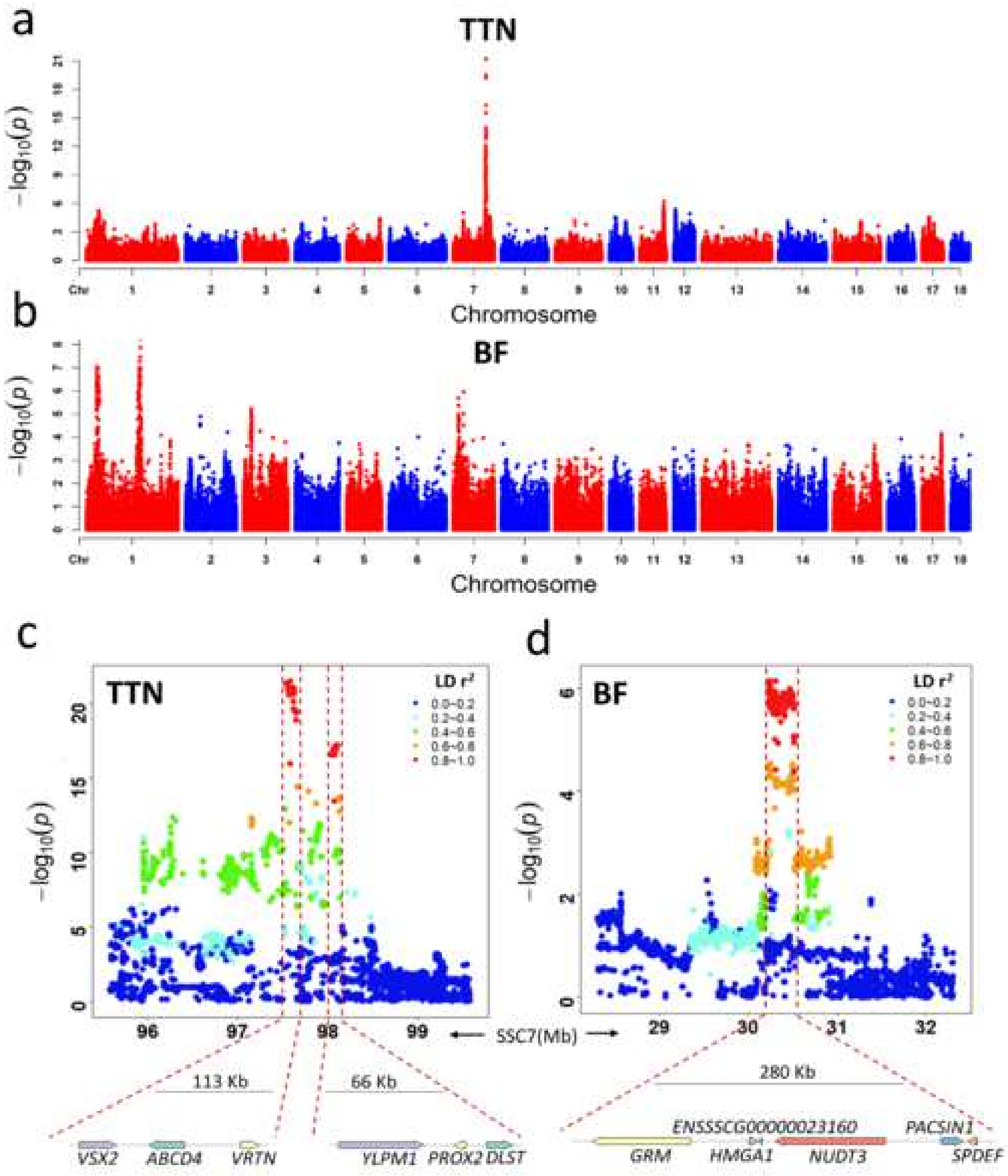

Figure 6

Click here to access/download;Figure;Figure 6.tif

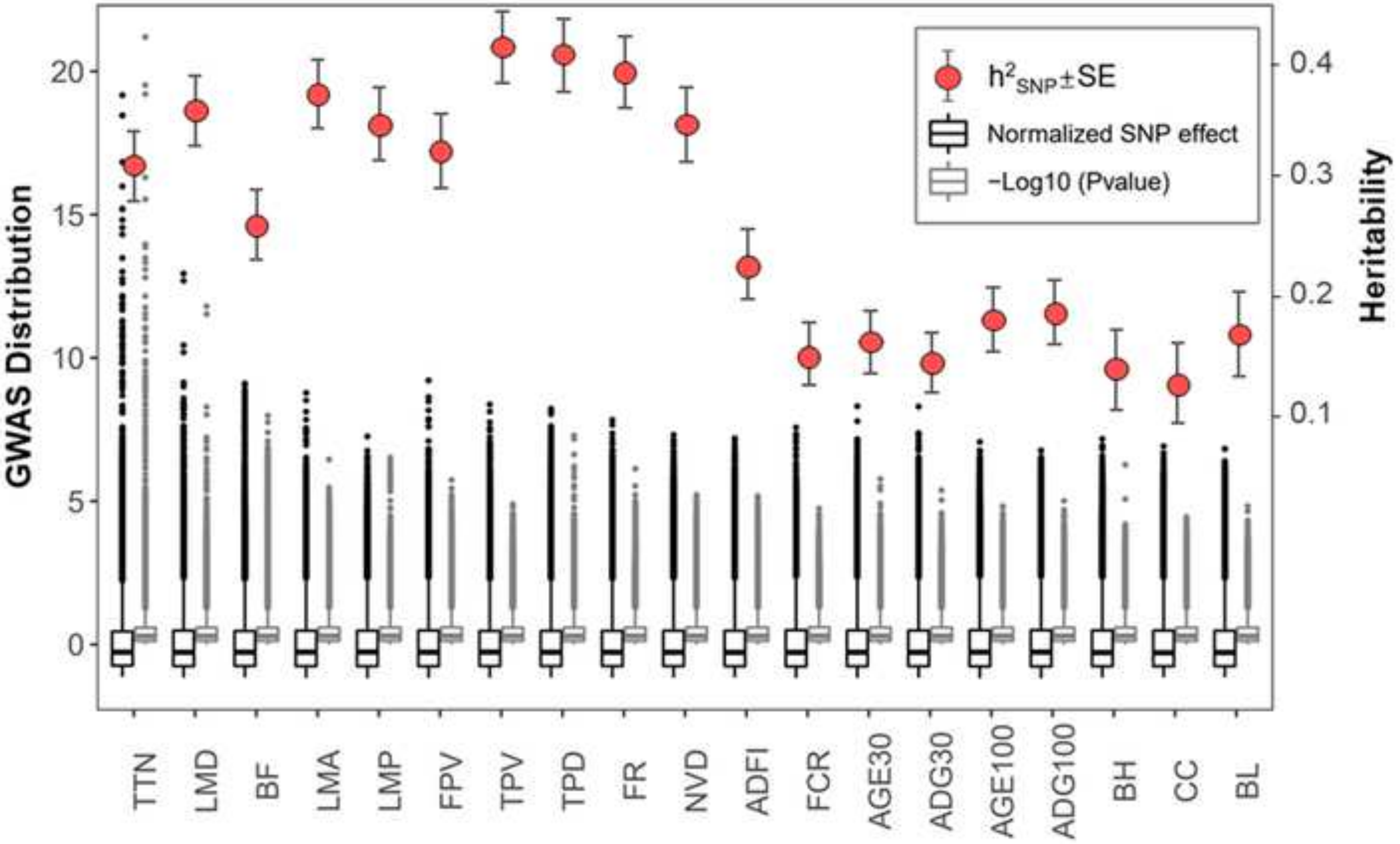

Supplementary Figure 1

Click here to access/download
Supplementary Material
Supplementary Figure 1.tif

Supplementary Figure 2

Click here to access/download
Supplementary Material
Supplementary Figure 2.tif

Supplementary Figure 3

Click here to access/download
Supplementary Material
Supplementary Figure 3.tif

Supplementary Figure 4

Click here to access/download
Supplementary Material
Supplementary Figure 4.tif

Supplementary Figure 5

Click here to access/download
Supplementary Material
Supplementary Figure 5.tif

Supplementary Figure 6

Click here to access/download
Supplementary Material
Supplementary Figure 6.tif

Supplementary Figure 7

Click here to access/download
Supplementary Material
Supplementary Figure 7.tif

Supplementary Figure 8

Click here to access/download
Supplementary Material
Supplementary Figure 8.tif

Supplementary Figure 9

Click here to access/download
Supplementary Material
Supplementary Figure 9.png

Supplementary Table S1

Click here to access/download
Supplementary Material
Supplementary Table S1.xlsx

Supplementary Table S2

Click here to access/download
Supplementary Material
Supplementary Table S2.xlsx

Supplementary Table S3

Click here to access/download
Supplementary Material
Supplementary Table S3.xlsx

Supplementary Table S4

Click here to access/download
Supplementary Material
Supplementary Table S4.xlsx

Supplementary Table S5

Click here to access/download
Supplementary Material
Supplementary Table S5.xlsx

Supplementary Table S6

Click here to access/download
Supplementary Material
Supplementary Table S6.xlsx

Supplementary Table S7

Click here to access/download
Supplementary Material
Supplementary Table S7.xlsx

Supplementary Table S8

Click here to access/download
Supplementary Material
Supplementary Table S8.xlsx

Supplementary Table S9

Click here to access/download
Supplementary Material
Supplementary Table S9.xlsx

Click here to access/download
Supplementary Material
Supplementary Table S10.xlsx

Supplementary Table S11

Click here to access/download
Supplementary Material
Supplementary Table S11.xlsx

Click here to access/download
Supplementary Material
Supplementary Table S12.xlsx

Click here to access/download
Supplementary Material
Supplementary Table S13.xlsx

Supplementary Table S14

Click here to access/download
Supplementary Material
Supplementary Table S14.xlsx

Personal Cover Click here to access/download;Personal Cover;covering_letter-0519.docx

May 19, 2021
Hans Zauner, PhD, Editor
GigaScience

Dear Editor:

We would like to sincerely thank the reviewers for the many helpful comments that have significantly improved the manuscript. We have addressed all comments of editor and reviewer2. We hereby re-submit a revised version of our manuscript entitled “Accelerated deciphering of the genetic architecture of agricultural economic traits in pigs using the low coverage whole-genome sequencing strategy” for publication in *GigaScience*.

Uncovering the genetic architecture of economic traits in pigs is important for agricultural breeding. However, whole genome sequencing of large cohorts would be too expensive, and accurate genotype imputation requires high-density haplotype reference panels that are unavailable in most agricultural populations due to their large size. Here, we report a Tn5-based, highly accurate, cost and time-efficient, low coverage sequencing (LCS) approach to perform sequencing on 2869 Duroc boars at an average depth of 0.73×, which identify 11.3 M SNPs throughout the genome. Base on the whole genome sequencing strategy, the high-resolution genome-wide association study (GWAS) detected 14 candidate quantitative trait loci (QTLs) in seven of 21 important traits and provided a lot of worth points for further investigation. We also showed that the artificial selection alters genomes that affect important growth traits. Moreover, we explored the different traits with varies genetic architecture in depth, providing guidance for subsequent genetic improvement by genomic selection. The LCS strategy, together with the unprecedented capacity of NGS allows the cost-effective and large-scale genome analysis with industrial-scale efficiency, and we are also confident that it will be a universal strategy to meet the needs for the genomic study and breeding of both animals and plants.

Thank you for your consideration. I look forward to hearing from you.

Sincerely,
Xiaoxiang Hu, Professor, Ph.D.
College of Biological Sciences
China Agricultural University
No.2 Yuanmingyuan West Road, Beijing 100193, P. R. China
Phone: +86-10-62733394
E-mail: huxx@cau.edu.cn
