# Supplementary figures and images for: Accelerated deciphering of the genetic architecture of agricultural economic traits in pigs using a low-coverage whole-genome sequencing strategy
Source: Gigascience. 2021 Jul 20;10(7):giab048. doi: 10.1093/gigascience/giab048 (PMC8290195; doi:10.1093/gigascience/giab048)

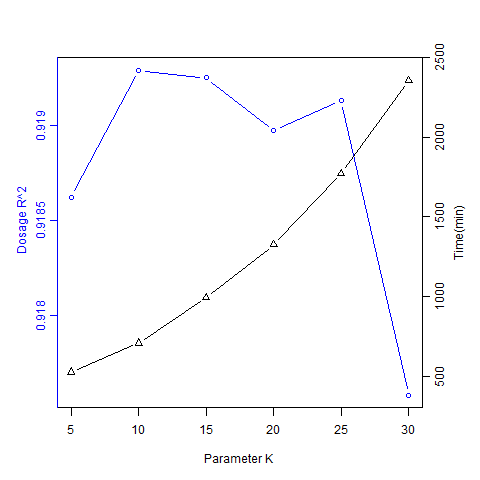

Supplement: giab048_Supplemental_Figures_and_Tables [file giab048_supplemental_figures_and_tables.zip › Supplementary Figure 2.tif]

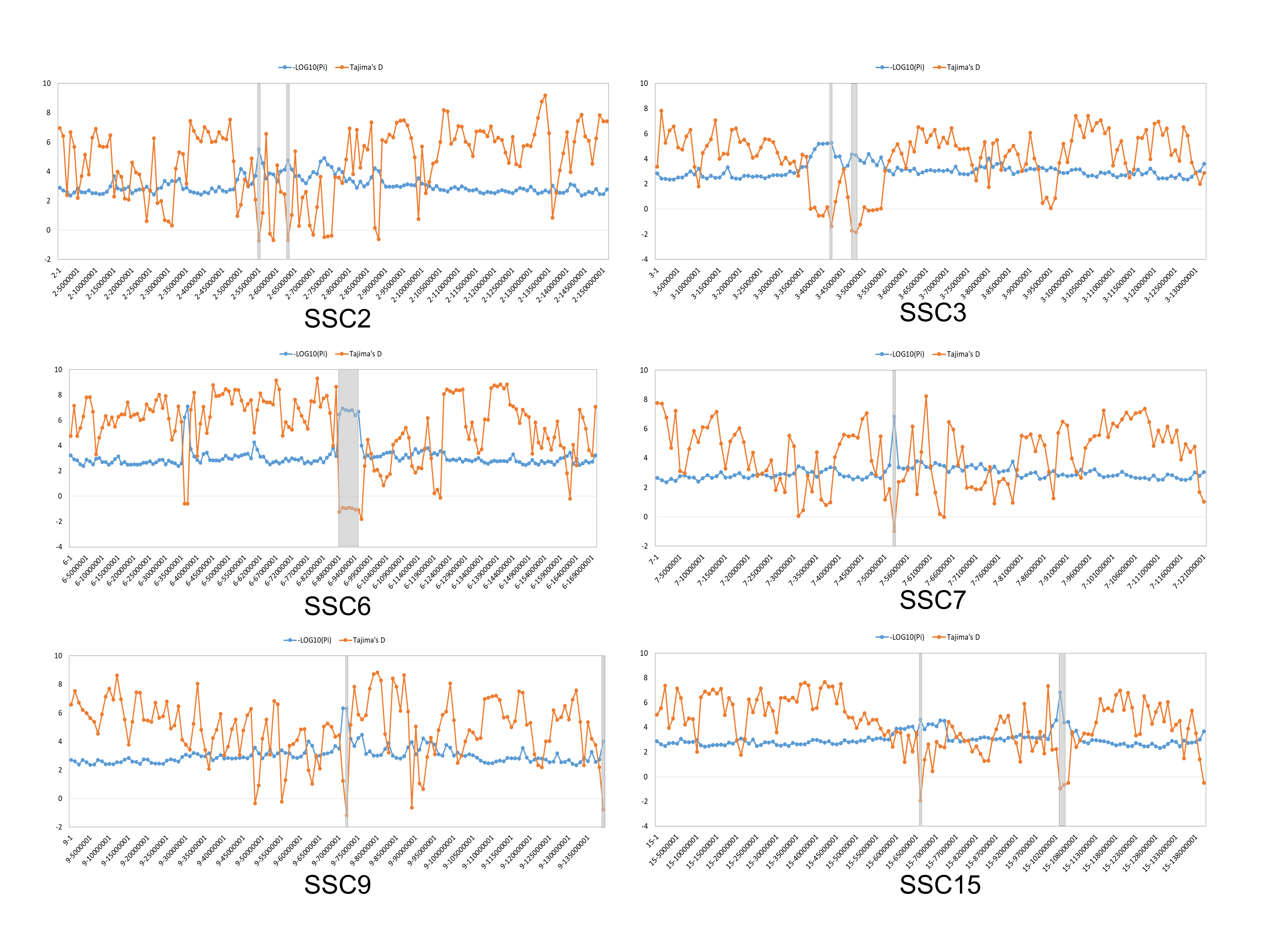

Supplement: giab048_Supplemental_Figures_and_Tables [file giab048_supplemental_figures_and_tables.zip › Supplementary Figure 3.tif]

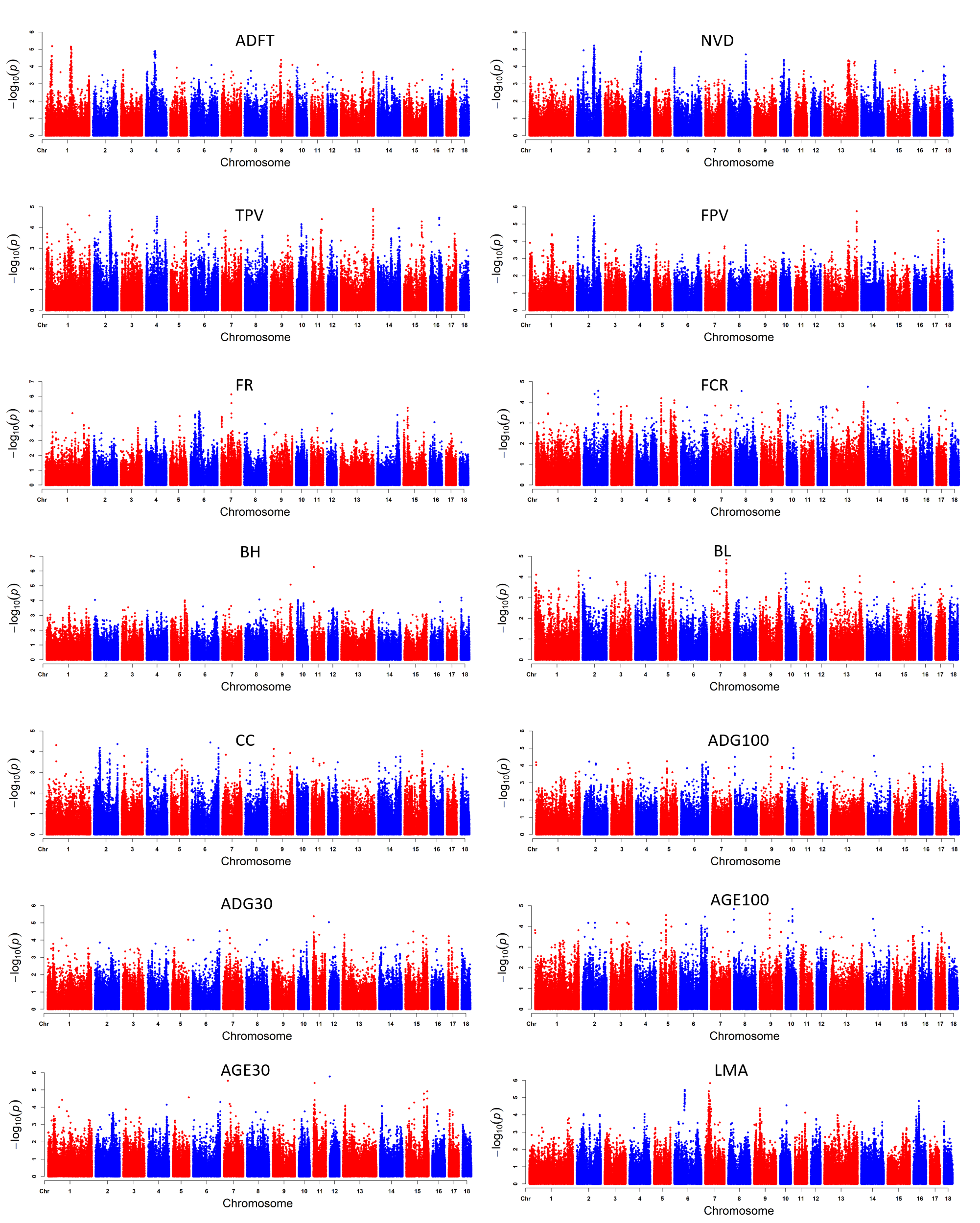

Supplement: giab048_Supplemental_Figures_and_Tables [file giab048_supplemental_figures_and_tables.zip › Supplementary Figure 4.tif]

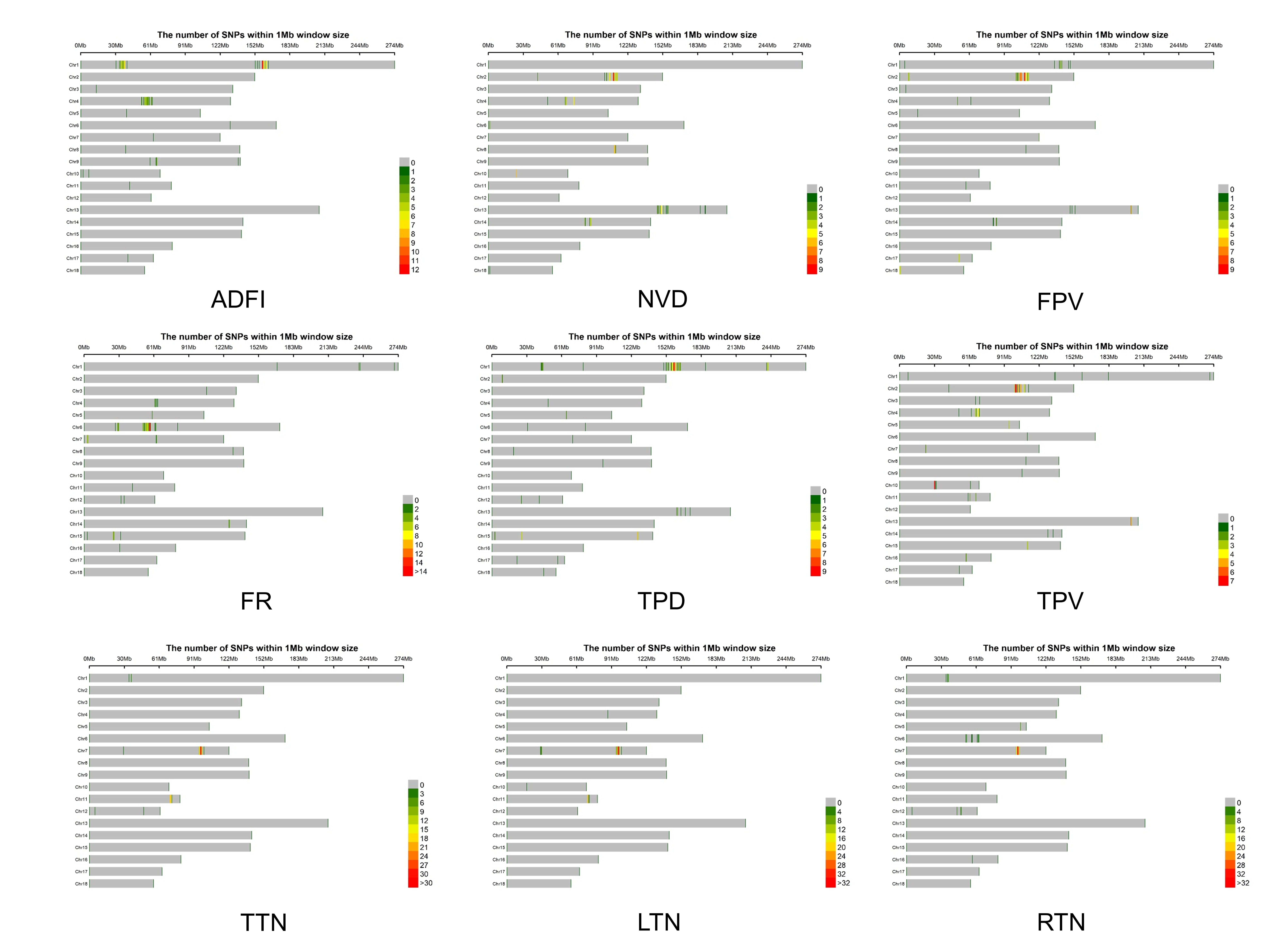

Supplement: giab048_Supplemental_Figures_and_Tables [file giab048_supplemental_figures_and_tables.zip › Supplementary Figure 7.tif]

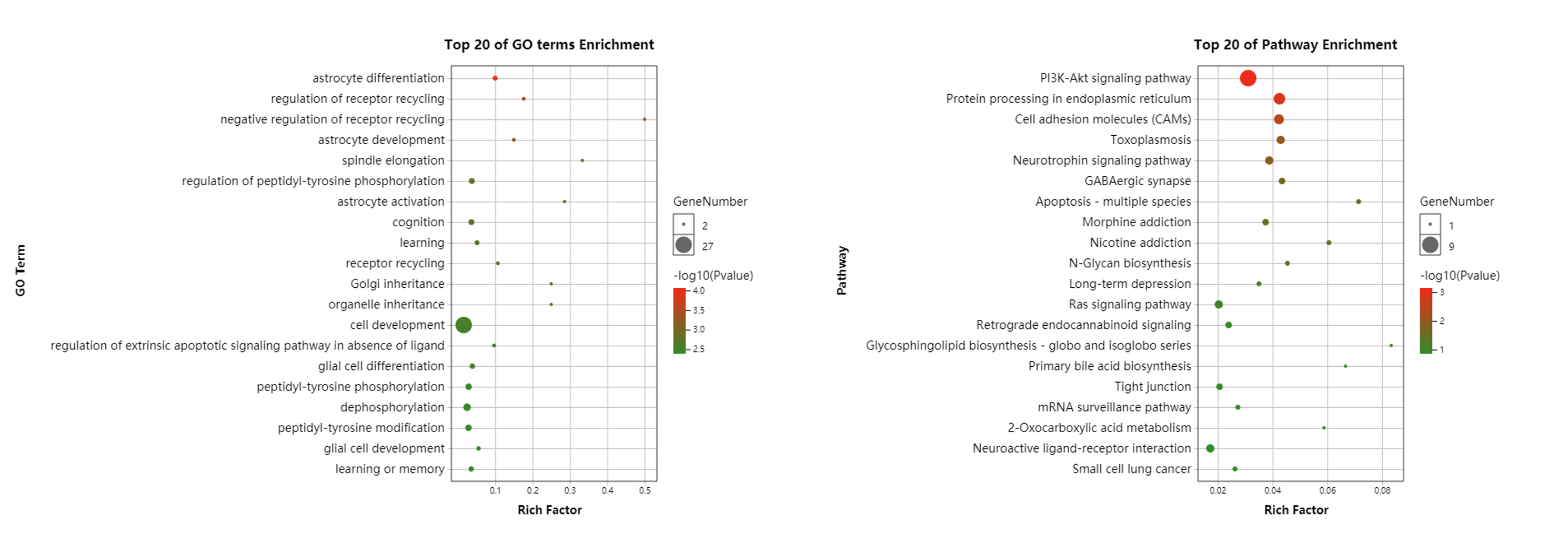

Supplement: giab048_Supplemental_Figures_and_Tables [file giab048_supplemental_figures_and_tables.zip › Supplementary Figure 8.tif]

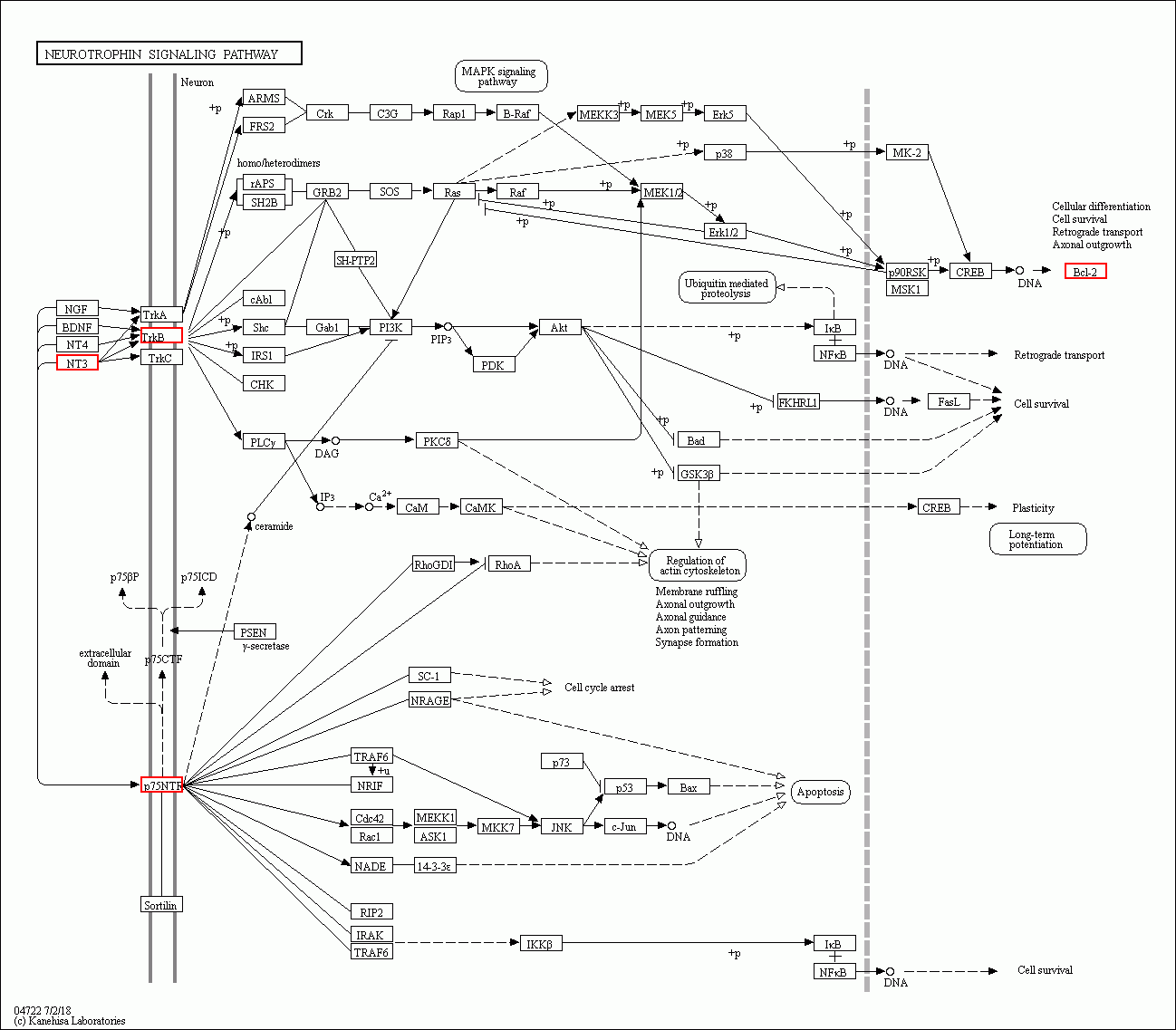

Supplement: giab048_Supplemental_Figures_and_Tables [file giab048_supplemental_figures_and_tables.zip › Supplementary Figure 9.png]
