# Supplementary material for: Accelerated deciphering of the genetic architecture of agricultural economic traits in pigs using a low-coverage whole-genome sequencing strategy
Source: Gigascience. 2021 Jul 20;10(7):giab048. doi: 10.1093/gigascience/giab048 (PMC8290195; doi:10.1093/gigascience/giab048)
Supplement: giab048_Response_to_Reviewer_Comments_Original_Submission [file giab048_response_to_reviewer_comments_original_submission.pdf]

Author's Response To Reviewer Comments

Close

Dear editor and reviewers,

We would like to sincerely thank the reviewers for the many helpful comments that have significantly improved the manuscript. We hereby re-submit a revised version of our manuscript entitled “Accelerated deciphering of the genetic architecture of agricultural economic traits in pigs using the low coverage whole-genome sequencing strategy” for publication in GigaScience.

For clarity, we have answered the questions from the reviewers point by point. We have formatted our manuscript according to the requirements of GigaScience (such as the “Result” section has changed to” Data Description” and ”Analyses”, and added “Potential Implications” section), and make sure that all raw sequencing data (from all 2869 pigs) is submitted to NCBI database. Please check it. Thank you!

If you have any questions, please just let me know. I am greatly looking forward to your response.

Sincerely,
Xiaoxiang Hu, Professor, Ph.D.
College of Biological Sciences
China Agricultural University
No.2 Yuanmingyuan West Road, Beijing 100193, P. R. China
Phone: +86-10-62733394
E-mail: huxx@cau.edu.cn

The response to comments from Reviewer 1

Reviewer #1: The manuscript by Yang and colleagues describes a protocol/approach for obtaining genomic markers from low coverage sequencing based on Tn5 transposase. The authors carried out WGS sequencing of 2,869 Duroc boars, obtaining an average depth of 0.73×/animal for a total of about 11.3 Million detected variants. For the detection of variants and imputation of the genotype, the authors compared two approaches: the first one base on GATK-Beagle and a second one base on BaseVar-STITCH, the latter resulting more suitable and appropriate when dealing with low coverage sequencing. After the detection of variants, the authors carried out GWAS analyses on more than 20 production and reproductive traits. Analyses included also estimation of heritability and functional annotation (gene enrichment analysis and functional impact evaluation).
Overall, the dataset can be described as large-scale, it is well described and provides a clear idea of its use. The manuscript provides a proper introduction, describing the problems in the filed and a possible way about how to deal with and counteract them. The authors addressed the data analysis in a proper way. They compared also different pipelines in order to identify the most appropriate one. Pipelines are clearly described. The obtained results have been properly interpreted and discussed.
Response: Thank you for your comments.

I have just some minor comments:

1. I suggest to carry out a direct genotyping of at least one SNP on SSC7 (related to the no. of teats), in order to confirm the goodness on imputation and to strengthen the obtained results.

Response: Thank you for your suggestion. Sixteen sites on SSC7 were selected based on the GWAS results, 3 of which were related to the BF and the others were related to the TN. Primers for genotyping were designed and ordered on the Fludigm D3 assay design website (new Supplementary Table S14), and 191 out of the total 2869 pigs were genotyped for each SNP using Fludigm Dynamic array IFC (Integrated Fluidic Circuit). Compared with the LC results, the average consistency GC = 0.991 (as shown in new Supplementary Table S3), which confirms the accuracy of the imputation obtained in LC study.
The result has been added as:

“Moreover, high depth resequencing (n=37, selected from the 2,869 boars, average 15.15×/sample), SNP Array (n=42, GeneSeek Genomic Profiler Porcine 80K SNP Array, GGP-80) genotyping and Fluidigm IFC direct genotyping (n=191 for 16 SNP loci) were performed on the selected Duroc core boars…”.
“Furthermore, direct genotyping (16 loci, 191 individuals) was carried out using the Fludigm dynamic array IFC. The average GC was 0.991 compared with the BaseVar-STITCH data (Supplementary Table S3), which is as high as the aforementioned results.”
We also modified Figure 1 to improve the analysis process.
The method “Direct genotyping by Fluidigm IFC technology” has been added as:
“Sixteen loci on SSC7 were selected based on the GWAS results, three of which were related to BF, and the others were related to TN. Primers for genotyping were designed and ordered on the Fludigm D3 assay design website (Supplementary Table S13), and 191 out of the total 2,869 pigs were genotyped for each SNP using Fludigm Dynamic array IFC (Integrated Fluidic Circuit).”

2. The dataset PRJNA681437 is linked to 58 different Duroc animals (the manuscript states 37 animals sequenced ad high-depth). What about the WGS of all the 2,869 pigs? They should be deposited as well. Moreover, the "doi" identifier of the *.vcf file deposited in GIGADB should be provided. At the moment, I can not verify what have been publicly released by the authors. The deposited VCF reports also the imputed genotypes?

Response: Thank you for your question. These 58 datasets are the raw sequencing data for low coverage sequencing using the BGI platform. Each data set contains about 96 samples. We have added the individual index information of each dataset to new Supplementary Table S13. We previously missed the raw sequencing data of 2 lanes using the Illumina platform, and now we have added these data to the NCBI PRJNA712489. The 37 high-depth resequencing data also has been uploaded to the PRJNA712489. We have submitted the VCF files to GigaDB. The deposited VCF reports the imputed genotypes. The editor's reply tells us that he will send the review access of VCF file to you. The respecting contents had been added on “Data availability” section as:
“All of the sequencing raw data in this study have been deposited into NCBI with accession number PRJNA681437, PRJNA712489 and the variance data as VCF file will be available in the GigaScience database. The individual index information of LCS dataset was listed on Supplementary Table S13.”

3. Details about gene enrichment (ORA or GSEA) should be provided, included the used statistics, the no. of analyzed terms (how many Biological processes? how many KEGG pathways?), the source of those terms (are they organism specific? Did you use GO and KEGG for S. scrofa?) and the usage of a FDR/Bonferroni correction procedure (including the alpha level).

Response: Thank you for your questions. In our study, the GO terms were downloaded from Ensembl website using BioMart tool, and KEGG pathway of each gene was corresponding to the NCBI website using OmicShare tools. The source GO and KEGG were pig specific. We did not use FDR/Bonferroni correction procedure for testing, for the procedure was too stringent and many enrichment pathways could be ignored. The respecting contents had been added as:
“The Gene Ontology (GO) terms were downloaded from the Ensembl website using the BioMart tool (http://asia.ensembl.org/biomart/martview/), and the Kyoto Encyclopedia of Genes and Genomes (KEGG) pathway was obtained according to the NCBI gene accession number, and both GO and KEGG terms were organism specific (S.scrofa). Finally, annotations of 335,522 GO terms and 6,139 KEGG pathways were retained for enrichment analyses. Both enrichment analyses were performed using the OmicShare tools (http://www.omicshare.com/tools), and the significance was determined by the P value according to the hypergeometric test ($P < 0.05$).”

4. Line 177. Reference paper is missing

Response: Thank you for your comments. We had added the reference paper: Paudel Y, et al. Copy number variation in the speciation of pigs: a possible prominent role for olfactory receptors. BMC Genomics. 2015;16:330. doi:10.1186/s12864-015-1449-9.

5. Line 257. Reference paper is missing.

Response: Thank you for your comments. We had added 5 associated reference papers:
(1) Tang Z, et al. Genome-Wide Association Study Reveals Candidate Genes for Growth Relevant Traits in Pigs. Front Genet. 2019;10:302. doi:10.3389/fgene.2019.00302.
(2) Fontanesi L, et al. A genome wide association study for average daily gain in Italian Large White pigs. J Anim Sci. 2014;92 4:1385-94. doi:10.2527/jas.2013-7059.

(3) Silva EF, et al. A genome-wide association study for feed efficiency-related traits in a crossbred pig population. Animal. 2019;13 11:2447-56. doi:10.1017/S1751731119000910.
(4) Qiao R, et al. Genome-wide association analyses reveal significant loci and strong candidate genes for growth and fatness traits in two pig populations. Genet Sel Evol. 2015;47:17. doi:10.1186/s12711-015-0089-5.
(5) Ding R, et al. Genetic Architecture of Feeding Behavior and Feed Efficiency in a Duroc Pig Population. Front Genet. 2018;9:220. doi:10.3389/fgene.2018.00220.

The response to comments from Reviewer 2

Reviewer #2: The authors have performed an extensive QTL analysis based on a large number of SNPs in a large Duroc population. The results presented show the power and cost-effectiveness of a low coverage sequencing strategy and increase our insight in the molecular mechanisms behind quantitative traits.

Response: Thank you for your comments.

1. Unfortunately, the paper is not written very well and at many places tends towards story telling. The authors point towards a large number of potential candidate genes, many of which have already been identified in previous studies to affect the traits studied in the current study. There is nothing wrong with that, but very often this results in an extensive discussion without any direct evidence that helps to further identify the causal variant responsible for the observed QTL. The discussion therefore could be much shortened which greatly would benefit the readability of the paper. The same is true for the results section, which for over 50% is already discussion rather than presenting the results. E.g. see the discussion about the ABCD4 gene in the results. Furthermore, the involvement of the ABCD4 gene on teat number has been extensively been discussed in several previously published studies.

Response: Thank you for your comments. We have checked the logical presentation of ideas and the structure of the paper, and drastically revised the discussion and results section of the manuscript: reduced the discussion related to gene function, and deleted the repeated discussion with result section as much as possible. We have also condensed the core ideas of some discussion paragraphs to make the article more readable. The analysis of the infinitesimal model was transferred to the discussion section to ensure the objectivity of the results. Moreover, this manuscript has been edited to ensure that the language is clear and free of errors. Please check it in the revised manuscript.

2. The authors often fail to provide proper references, and where they do the references mentioned do not always provide evidence for the claims that are made. Some examples: Lines 175-177: Refers to a previous study but no reference is shown.

Response: Thank you for your comments. We had added the reference paper: Paudel Y, et al. Copy number variation in the speciation of pigs: a possible prominent role for olfactory receptors. BMC Genomics. 2015;16:330. doi:10.1186/s12864-015-1449-9.
We also examined other similar issues and made sure that all relevant references had been added in this manuscript.

3. Line 210: Refers to a former study reporting PROX2 could be the causal gene. But again, the reference of this study is not provided.

Response: Thank you for your comments. We had added 2 associated reference papers:
(1) Tan C, et al. Genome-wide association study and accuracy of genomic prediction for teat number in Duroc pigs using genotyping-by-sequencing. Genetics Selection Evolution. 2017;49 doi:ARTN 35 10.1186/s12711-017-0311-8
(2) Ren DR, et al. Mapping and fine mapping of quantitative trait loci for the number of vertebrae in a White Duroc x Chinese Erhualian intercross resource population. Anim Genet. 2012;43 5:545-51. doi:10.1111/j.1365-2052.2011.02313.x.

4. Line 57 states recently developed methods, yet the references are for papers up to 10 years old. I wouldn't call that "recent".

Response: Thank you for your comments. The concept of LCS method had been proposed for years, we therefore removed the impertinent statement “recently developed methods”.

5. Line 71: Reference 21 is rather old to be used in this context.

Response: Thank you for your comments. We had added several references relating to genome sequencing project in human:
(1) GenomeAsia KC. The GenomeAsia 100K Project enables genetic discoveries across Asia. Nature. 2019; 576 7785:106-11. doi:10.1038/s41586-019-1793-z.
(2) Wang Q, et al. Landscape of multi-nucleotide variants in 125,748 human exomes and 15,708 genomes. Nat Commun. 2020; 11 1:2539. doi:10.1038/s41467-019-12438-5.
(3) Genome of the Netherlands C. Whole-genome sequence variation, population structure and demographic history of the Dutch population. Nat Genet. 2014;46 8:818-25. doi:10.1038/ng.3021.
(4) Gudbjartsson DF, et al. Sequence variants from whole genome sequencing a large group of Icelanders. Sci Data. 2015; 2:150011. doi:10.1038/sdata.2015.11.

6. Line 329: References 40 and 41 are not good references for the statement made in lines 326-329.

Response: Thank you for your comments. Preselecting SNPs contribute to phenotype can improve the genomic predictive ability using optimized prediction methods, such as the genomic-feature BLUP model (GFBLUP) which had been proposed to improve GBLUP calculations. Here, we therefore modified the respecting contents as “Third, significantly improved GS results were observed when SNPs were preselected from the sequenced data with prior information and an optimized genomic prediction method considering genomic features (e.g. GFBLUP [55, 56])” and added related references:
[55] Edwards SM, et al. Genomic Prediction for Quantitative Traits Is Improved by Mapping Variants to Gene Ontology Categories in Drosophila melanogaster. Genetics. 2016; 203 4:1871-83. doi:10.1534/genetics.116.187161.
[56] Xiang R, et al. Quantifying the contribution of sequence variants with regulatory and evolutionary significance to 34 bovine complex traits. Proc Natl Acad Sci U S A. 2019;116 39:19398-408. doi:10.1073/pnas.1904159116.

7. For the evaluation of the SNP calling procedure based on BaseVar-STITCH (lines 108-137) it is unclear exactly what data sets are used and how reliably individual genotypes are for animals that have only be sequenced at a very low coverage. This paragraph needs to be clarified.

Response: Thank you for your question. We sequenced 37 out of the total 2,869 pigs at a high depth(~15×/individual). We also selected 42 individuals who were included in the LCS dataset and genotyped using the GeneSeek Genomic Profiler Porcine 80K SNP Array. These two datasets (high-depth sequencing and SNP chip result on chromosome 18) were used as the two gold standards for accuracy evaluation of LCS data. The reported GC and R2 value refer to the result of comparing LCS data with high-depth sequencing (or SNP chip) of 37 (or 42) samples. The respecting contents had been revised as:
“in this study, we mainly applied the BaseVar algorithm [33] to identify polymorphic sites and infer allele frequencies, and STITCH [15] to impute SNPs.” ... “The high-depth sequencing data and SNP chip (GGP-80) results on SSC18 were used as the gold standard for accuracy evaluation (Fig. 1 and Supplementary Table S2).”
We believe that these 37 (or 42) samples are representative for other samples because the variance of accuracy is very small. In additional, as the response to question 1 from reviewer 1, 16 loci on another chromosome (SSC7) were random selected. 191 out of the total 2869 pigs were directly genotyped for each SNP using Fludigm Dynamic array IFC (Integrated Fluidic Circuit). Compared with the LC results, the average consistency GC is more than 0.99 (new Supplementary Table 3), which confirms the accuracy of the imputation obtained in LC study. The respecting description had been added as: “Furthermore, direct genotyping (16 loci, 191 individuals) was carried out using the Fludigm dynamic array IFC. The average GC was 0.991 compared with the BaseVar-STITCH data (Supplementary Table S3), which is as high as the aforementioned results. Taken together, these results suggest that BaseVar-STITCH pipeline is a suitable variant discovery and imputation method for the LCS strategy (Fig. 1).”
We also modified new Figure 1 to improve the analysis process.

8. Lines 397-398: The comment "delivers fewer loci for fewer phenotypes" is rather odd. Fewer than what? And why would that be fewer? Is this statement based on other studies, on the estimated heritabilities?

Response: Sorry for the unclear description. In our study, we found some traits with very few QTL with significant SNPs, we summarized possible reasons of these results including phenotypes were under

long-term artificial selection (has been discussed in the previous paragraph) or with the infinitesimal model for high heritability but the lack of major QTL.
On this paragraph, the respecting contents had been revised as “Fewer QTLs with significant SNPs were detected in feeding behaviour traits and body size measurements than in teat number and carcass traits. These observations are interpreted in a paradigm in which complex traits are driven by an accumulation of weak regulatory effects on the large genes and regulatory pathways [63-65], i.e. ‘infinitesimal model’.”

9. The authors studies 21 different phenotypes. However, many of these are highly correlated and this should be stated more clearly.

Response: Thank you for your question. The genetic and phenotypic correlation coefficient of the 21 phenotypes has been reported in new Supplementary Table S6. The respecting contents had been added as ”There was high correlation between traits of the same type (such as LMD, LMA and LMP; BH, BL and CC, Supplementary Table S6).”

Minor comments:

10. Line 19: "populations"

Response: This has been corrected.

11. Line 18-21: This is not a good English sentence

Response: This has been modified as “high-density haplotype reference panels are unavailable in most agricultural species, limiting accurate genotype imputation in large populations. Moreover, the infinitesimal model of quantitative traits implies that weak association signals tend to be spread across most of the genome, further complicating the genetic analysis”.

12. Line 22: Replace "discovered" by "describe"

Response: This has been implemented.

13. Lines 22-25: This reads like the authors have performed LCS on all animals and then in addition have also done whole genome sequencing of all individuals.

Response: This has been modified as “We described a Tn5-based highly accurate, cost- and time-efficient, low coverage sequencing (LCS) method to obtain 11.3 M whole genome SNPs in 2,869 Duroc boars at an average depth of 0.73×.”.

14. Line 26: replace "in" by "for"

Response: This has been implemented.

15. Line 36: insert "can be" between "and widely".

Response: This has been implemented.

16. Line 45: "relies"

Response: This has been corrected.

17. Line 45: Strange sentence "which perceive linkage"

Response: This has been modified as “The mapping resolution relies on the density of genetic markers that can reveal linkage disequilibrium (LD) patterns in sufficiently large populations.

18. Line 74: "describes"

Response: This has been corrected.

19. Line 74-75: The infinitesimal model is not specific for "human quantitative traits". Change sentence.

Response: The “human quantitative traits” has been changed to “quantitative traits”.

20. Line 79: Replace second "process" by "produce"

Response: This has been implemented.

Close
