# Supplementary material for: Accelerated deciphering of the genetic architecture of agricultural economic traits in pigs using a low-coverage whole-genome sequencing strategy
Source: Gigascience. 2021 Jul 20;10(7):giab048. doi: 10.1093/gigascience/giab048 (PMC8290195; doi:10.1093/gigascience/giab048)
Supplement: giab048_Response_to_Reviewer_Comments_Revision_1 [file giab048_response_to_reviewer_comments_revision_1.pdf]

Author's Response To Reviewer Comments

Close

Dear editor and reviewers,

We would like to sincerely thank your helpful comments. We hereby re-submit a revised version of our manuscript entitled “Accelerated deciphering of the genetic architecture of agricultural economic traits in pigs using the low coverage whole-genome sequencing strategy” for publication in GigaScience.

We have addressed all comments of editor and reviewer2. For clarity, here we answered the questions point by point. If you have any questions, please just let me know. I am greatly looking forward to your response.

Sincerely,
Xiaoxiang Hu, Professor, Ph.D.
College of Biological Sciences
China Agricultural University
No.2 Yuanmingyuan West Road, Beijing 100193, P. R. China
Phone: +86-10-62733394
E-mail: huxx@cau.edu.cn

The response to comments from Editor
1.Please note that you can indicate a maximum of two "corresponding authors" (but you can also indicate a role for a "senior author", if you wish).
Response: The two corresponding authors are Dr. Yuzhe Wang and Dr. Zhenfang Wu. Professor Xiaoxiang Hu as the senior author of this article.

2. Our data curators will contact you shortly to prepare the supporting data for release via GigaDB. Please include a citation to your upcoming GigaDB dataset (including the DOI link) to your reference list, and cite this in the data availability section and elsewhere in the manuscript, where appropriate.
Response: This GigaDB dataset has been cited as “An archival copy of the code and supporting data is available via the GigaScience repository, GigaDB [75].”

The response to comments from Reviewer 2

Page 2, line 8: Change "We described a" to "We describe a"
Response: This has been corrected.

Page 2, lines 10-13: I suggest changing this long sentence to the following two sentences: "Based on these SNPs, a genome-wide association study (GWAS) was performed resulting in 14 quantitative trait loci (QTLs) for seven of 21 important agricultural traits in pigs. These QTLs harbour genes, such as ABCD4 for total teat number and HMGA1 for back fat thickness and provided a starting point for further investigation."
Response: This has been corrected.

Page 4, line 28: I suggest changing this sentence to: "The results generated by the BGI platform had a smaller number of PCR duplicates (2.23%), a higher number of index reads (97.10%),……."
Response: This has been corrected.

Page 5, line 3: Insert "the" between "and" and "majority"
Response: This has been corrected.

Page 6, line 12: "Fluidigm" instead of "Fludigm"
Response: This has been corrected.

Page 7, line 2; Replace "in all" by "for all" or by "using all"
Response: This has been corrected.

Page 7, line 17-18: "We further studied the high level of LD, and found that it could be a consequence of long-term strong natural or artificial selection." I don't agree with this statement. The high LD is the result of the small effective population size used for these breeding lines.
Response: We have deleted this sentence.

Page 8, line 13: Replace "each" by "individual"
Response: This has been corrected.

Page 8, line 15: "We first focused on QTLs that could be narrowed". The authors do not show how this is done. Based on what information could these QTL be narrowed?
Response: We have added detailed analysis methods and parameters in the method-"Genome-wide association and Heritability estimation" section: "Once a QTL had been mapped using the tagging SNPs and exceeded the FDR threshold, association was re-calculated with all imputed SNPs (from the 11.3 M set) in a 20 Mb window around the peak using the same mixed model. The definition of a fine-mapped candidate QTL interval was based on the linkage disequilibrium (LD) level between the most significant SNP and all flanking sites, where the boundary was verified when the LD was no more than 0.8".

Page 10, line 3: Change "which in turn led to the result of no significant QTL." To "which in turn resulted in no significant QTL."
Response: This has been corrected.

Close
