# Supplementary material for: Accelerated deciphering of the genetic architecture of agricultural economic traits in pigs using a low-coverage whole-genome sequencing strategy
Source: Gigascience. 2021 Jul 20;10(7):giab048. doi: 10.1093/gigascience/giab048 (PMC8290195; doi:10.1093/gigascience/giab048)
Supplement: giab048_Reviewer_1_Report_Original_Submission — Samuele Bovo, Ph.D. -- 1/7/2021 Reviewed [file giab048_reviewer_1_report_original_submission.pdf]

Reviewer Report

Title: Accelerated deciphering of the genetic architecture of agricultural economic traits in pigs using the low coverage whole-genome sequencing strategy

Version: Original Submission **Date:** 1/7/2021

Reviewer name: Samuele Bovo, Ph.D.

Reviewer Comments to Author:

The manuscript by Yang and colleagues describes a protocol/approach for obtaining genomic markers from low coverage sequencing based on Tn5 transposase. The authors carried out WGS sequencing of 2,869 Duroc boars, obtaining an average depth of 0.73Ã—/animal for a total of about 11.3 Million detected variants. For the detection of variants and imputation of the genotype, the authors compared two approaches: the first one base on GATK-Beagle and a second one base on BaseVar-STITCH, the latter resulting more suitable and appropriate when dealing with low coverage sequencing. After the detection of variants, the authors carried out GWAS analyses on more than 20 production and reproductive traits. Analyses included also estimation of heritability and functional annotation (gene enrichment analysis and functional impact evaluation).

Overall, the dataset can be described as large-scale, it is well described and provides a clear idea of its use. The manuscript provides a proper introduction, describing the problems in the filed and a possible way about how to deal with and counteract them. The authors addressed the data analysis in a proper way. They compared also different pipelines in order to identify the most appropriate one. Pipelines are clearly described. The obtained results have been properly interpreted and discussed.

I have just some minor comments:

1. I suggest to carry out a direct genotyping of at least one SNP on SSC7 (related to the no. of teats), in order to confirm the goodness on imputation and to strengthen the obtained results.
2. The dataset PRJNA681437 is linked to 58 different Duroc animals (the manuscript states 37 animals sequenced ad high-depth). What about the WGS of all the 2,869 pigs? They should be deposited as well. Moreover, the "doi" identifier of the *.vcf file deposited in GIGADB should be provided. At the moment, I can not verify what have been publicly released by the authors. The deposited VCF reports also the imputed genotypes?
3. Details about gene enrichment (ORA or GSEA) should be provided, included the used statistics, the no. of analyzed terms (how many Biological processes? how many KEGG pathways?), the source of those terms (are they organism specific? Did you use GO and KEGG for S. scrofa?) and the usage of a FDR/Bonferroni correction procedure (including the alpha level).
4. Line 177. Reference paper is missing
5. Line 257. Reference paper is missing.

Methods

Are the methods appropriate to the aims of the study, are they well described, and are necessary controls included? Choose an item.

Conclusions

Are the conclusions adequately supported by the data shown? Choose an item.

Reporting Standards

Does the manuscript adhere to the journal's guidelines on minimum standards of reporting? Choose an item.

Choose an item.

Statistics

Are you able to assess all statistics in the manuscript, including the appropriateness of statistical tests used? Choose an item.

Quality of Written English

Please indicate the quality of language in the manuscript: Choose an item.

Declaration of Competing Interests

Please complete a declaration of competing interests, considering the following questions:

- Have you in the past five years received reimbursements, fees, funding, or salary from an organisation that may in any way gain or lose financially from the publication of this manuscript, either now or in the future?
- Do you hold any stocks or shares in an organisation that may in any way gain or lose financially from the publication of this manuscript, either now or in the future?
- Do you hold or are you currently applying for any patents relating to the content of the manuscript?
- Have you received reimbursements, fees, funding, or salary from an organization that holds or has applied for patents relating to the content of the manuscript?
- Do you have any other financial competing interests?
- Do you have any non-financial competing interests in relation to this paper?

If you can answer no to all of the above, write 'I declare that I have no competing interests' below. If your reply is yes to any, please give details below.

I declare that I have no competing interests

I agree to the open peer review policy of the journal. I understand that my name will be included on my report to the authors and, if the manuscript is accepted for publication, my named report including any attachments I upload will be posted on the website along with the authors' responses. I agree for my

report to be made available under an Open Access Creative Commons CC-BY license (http://creativecommons.org/licenses/by/4.0/). I understand that any comments which I do not wish to be included in my named report can be included as confidential comments to the editors, which will not be published.

Choose an item.

To further support our reviewers, we have joined with Publons, where you can gain additional credit to further highlight your hard work (see: https://publons.com/journal/530/gigascience). On publication of this paper, your review will be automatically added to Publons, you can then choose whether or not to claim your Publons credit. I understand this statement.

Yes Choose an item.
