# Supplementary material for: Accelerated deciphering of the genetic architecture of agricultural economic traits in pigs using a low-coverage whole-genome sequencing strategy
Source: Gigascience. 2021 Jul 20;10(7):giab048. doi: 10.1093/gigascience/giab048 (PMC8290195; doi:10.1093/gigascience/giab048)
Supplement: giab048_Reviewer_2_Report_Original_Submission — Martien Groenen -- 1/18/2021 Reviewed [file giab048_reviewer_2_report_original_submission.pdf]

Reviewer Report

Title: Accelerated deciphering of the genetic architecture of agricultural economic traits in pigs using the low coverage whole-genome sequencing strategy

Version: Original Submission **Date:** 1/18/2021

Reviewer name: Martien Groenen

Reviewer Comments to Author:

The authors have performed an extensive QTL analysis based on a large number of SNPs in a large Duroc population. The results presented show the power and cost-effectiveness of a low coverage sequencing strategy and increase our insight in the molecular mechanisms behind quantitative traits. Unfortunately, the paper is not written very well and at many places tends towards story telling. The authors point towards a large number of potential candidate genes, many of which have already been identified in previous studies to affect the traits studied in the current study. There is nothing wrong with that, but very often this results in an extensive discussion without any direct evidence that helps to further identify the causal variant responsible for the observed QTL. The discussion therefore could be much shortened which greatly would benefit the readability of the paper. The same is true for the results section, which for over 50% is already discussion rather than presenting the results. E.g. see the discussion about the ABCD4 gene in the results. Furthermore, the involvement of the ABCD4 gene on teat number has been extensively been discussed in several previously published studies.
The authors often fail to provide proper references, and where they do the references mentioned do not always provide evidence for the claims that are made. Some examples:
Lines 175-177: Refers to a previous study but no reference is shown.
Line 210: Refers to a former study reporting PROX2 could be the causal gene. But again, the reference of this study is not provided.
Line 57 states recently developed methods, yet the references are for papers up to 10 years old. I wouldn't call that "recent".
Line 71: Reference 21 is rather old to be used in this context.
Line 329: References 40 and 41 are not good references for the statement made in lines 326-329.
For the evaluation of the SNP calling procedure based on BaseVar-STITCH (lines 108-137) it is unclear exactly what data sets are used and how reliably individual genotypes are for animals that have only be sequenced at a very low coverage. This paragraph needs to be clarified.
Lines 397-398: The comment "delivers fewer loci for fewer phenotypes" is rather odd. Fewer than what? And why would that be fewer? Is this statement based on other studies, on the estimated heritabilities?
The authors studies 21 different phenotypes. However, many of these are highly correlated and this should be stated more clearly.
Minor comments:
Line 19: "populations"
Line 18-21: This is not a good English sentence
Line 22: Replace "discovered" by "describe"

Lines 22-25: This reads like the authors have performed LCS on all animals and then in addition have also done whole genome sequencing of all individuals.
Line 26: replace "in" by "for"
Line 36: insert "can be" between "and widely".
Line 45: "relies"
Line 45: Strange sentence "which perceive linkage"
Line 74: "describes"
Line 74-75: The infinitesimal model is not specific for "human quantitative traits". Change sentence.
Line 79: Replace second "process" by "produce"

Methods

Are the methods appropriate to the aims of the study, are they well described, and are necessary controls included? Choose an item.

Conclusions

Are the conclusions adequately supported by the data shown? Choose an item.

Reporting Standards

Does the manuscript adhere to the journal's guidelines on minimum standards of reporting? Choose an item.

Choose an item.

Statistics

Are you able to assess all statistics in the manuscript, including the appropriateness of statistical tests used? Choose an item.

Quality of Written English

Please indicate the quality of language in the manuscript: Choose an item.

Declaration of Competing Interests

Please complete a declaration of competing interests, considering the following questions:

- Have you in the past five years received reimbursements, fees, funding, or salary from an organisation that may in any way gain or lose financially from the publication of this manuscript, either now or in the future?
- Do you hold any stocks or shares in an organisation that may in any way gain or lose financially from the publication of this manuscript, either now or in the future?

- Do you hold or are you currently applying for any patents relating to the content of the manuscript?
- Have you received reimbursements, fees, funding, or salary from an organization that holds or has applied for patents relating to the content of the manuscript?
- Do you have any other financial competing interests?
- Do you have any non-financial competing interests in relation to this paper?

If you can answer no to all of the above, write 'I declare that I have no competing interests' below. If your reply is yes to any, please give details below.

I declare that I have no competing interests

I agree to the open peer review policy of the journal. I understand that my name will be included on my report to the authors and, if the manuscript is accepted for publication, my named report including any attachments I upload will be posted on the website along with the authors' responses. I agree for my report to be made available under an Open Access Creative Commons CC-BY license (http://creativecommons.org/licenses/by/4.0/). I understand that any comments which I do not wish to be included in my named report can be included as confidential comments to the editors, which will not be published.

Choose an item.

To further support our reviewers, we have joined with Publons, where you can gain additional credit to further highlight your hard work (see: https://publons.com/journal/530/gigascience). On publication of this paper, your review will be automatically added to Publons, you can then choose whether or not to claim your Publons credit. I understand this statement.

Yes Choose an item.
