# Supplementary material for: Accelerated deciphering of the genetic architecture of agricultural economic traits in pigs using a low-coverage whole-genome sequencing strategy
Source: Gigascience. 2021 Jul 20;10(7):giab048. doi: 10.1093/gigascience/giab048 (PMC8290195; doi:10.1093/gigascience/giab048)
Supplement: giab048_Reviewer_2_Report_Revision_1 — Martien Groenen -- 4/16/2021 Reviewed [file giab048_reviewer_2_report_revision_1.pdf]

Reviewer Report

Title: Accelerated deciphering of the genetic architecture of agricultural economic traits in pigs using the low coverage whole-genome sequencing strategy

Version: Revision 1 **Date:** 4/16/2021

Reviewer name: Martien Groenen

Reviewer Comments to Author:

The manuscript has been greatly improved and the authors have addressed my comments made on the original version of the manuscript. This has enormously improved the readability of the current manuscript. I only have a few minor comments, most of which concern some further small suggestions to improve the English.
Page 2, line 8: Change "We described a" to "We describe a"
Page 2, lines 10-13: I suggest changing this long sentence to the following two sentences: "Based on these SNPs, a genome-wide association study (GWAS) was performed resulting in 14 quantitative trait loci (QTLs) for seven of 21 important agricultural traits in pigs. These QTLs harbour genes, such as ABCD4 for total teat number and HMGA1 for back fat thickness and provided a starting point for further investigation."
Page 4, line 28: I suggest changing this sentence to: "The results generated by the BGI platform had a smaller number of PCR duplicates (2.23%), a higher number of index reads (97.10%),……."
Page 5, line 3: Insert "the" between "and" and "majority"
Page 6, line 12: "Fluidigm" instead of "Fludigm"
Page 7, line 2; Replace "in all" by "for all" or by "using all"
Page 7, line 17-18: "We further studied the high level of LD, and found that it could be a consequence of long-term strong natural or artificial selection." I don't agree with this statement. The high LD is the result of the small effective population size used for these breeding lines.
Page 8, line 13: Replace "each" by "individual"
Page 8, line 15: "We first focused on QTLs that could be narrowed". The authors do not show how this is done. Based on what information could these QTL be narrowed?
Page 10, line 3: Change "which in turn led to the result of no significant QTL." To "which in turn resulted in no significant QTL."

Methods

Are the methods appropriate to the aims of the study, are they well described, and are necessary controls included? Choose an item.

Conclusions

Are the conclusions adequately supported by the data shown? Choose an item.

Reporting Standards

Does the manuscript adhere to the journal's guidelines on [minimum standards of reporting?]() Choose an item.

Choose an item.

Statistics

Are you able to assess all statistics in the manuscript, including the appropriateness of statistical tests used? Choose an item.

Quality of Written English

Please indicate the quality of language in the manuscript: Choose an item.

Declaration of Competing Interests

Please complete a declaration of competing interests, considering the following questions:

- Have you in the past five years received reimbursements, fees, funding, or salary from an organisation that may in any way gain or lose financially from the publication of this manuscript, either now or in the future?
- Do you hold any stocks or shares in an organisation that may in any way gain or lose financially from the publication of this manuscript, either now or in the future?
- Do you hold or are you currently applying for any patents relating to the content of the manuscript?
- Have you received reimbursements, fees, funding, or salary from an organization that holds or has applied for patents relating to the content of the manuscript?
- Do you have any other financial competing interests?
- Do you have any non-financial competing interests in relation to this paper?

If you can answer no to all of the above, write 'I declare that I have no competing interests' below. If your reply is yes to any, please give details below.

I declare that I have no competing interests

I agree to the open peer review policy of the journal. I understand that my name will be included on my report to the authors and, if the manuscript is accepted for publication, my named report including any attachments I upload will be posted on the website along with the authors' responses. I agree for my report to be made available under an Open Access Creative Commons CC-BY license (http://creativecommons.org/licenses/by/4.0/). I understand that any comments which I do not wish to be included in my named report can be included as confidential comments to the editors, which will not be published.

Choose an item.

To further support our reviewers, we have joined with Publons, where you can gain additional credit to further highlight your hard work (see: https://publons.com/journal/530/gigascience). On publication of this paper, your review will be automatically added to Publons, you can then choose whether or not to claim your Publons credit. I understand this statement.

Yes Choose an item.
